# Supplementary figures and images for: Exploration of schizophrenia-associated gene modules using graph theory, co-expression networks, and dimensionality reduction
Source: PLoS One. 2026 Apr 15;21(4):e0346663. doi: 10.1371/journal.pone.0346663 (PMC13082716; doi:10.1371/journal.pone.0346663)

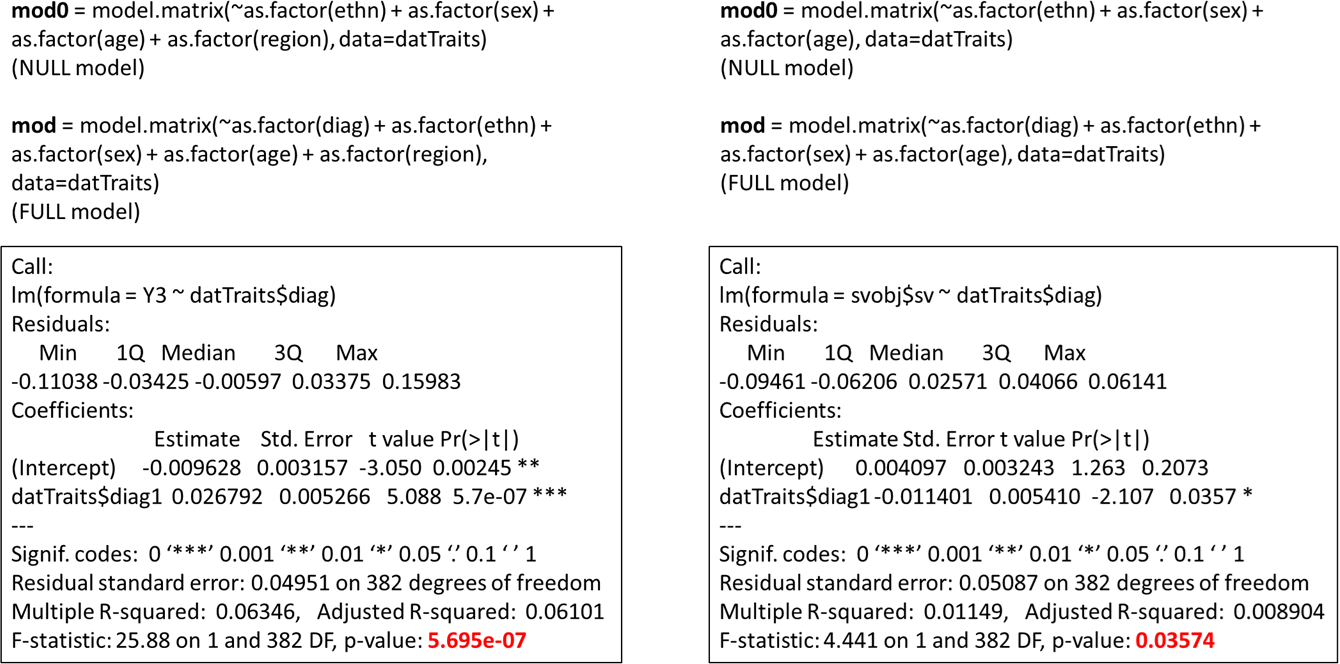

Supplement: S1 Fig — Full code and model matrices are available in the Supplementary R scripts. (TIF) [file pone.0346663.s001.tif]

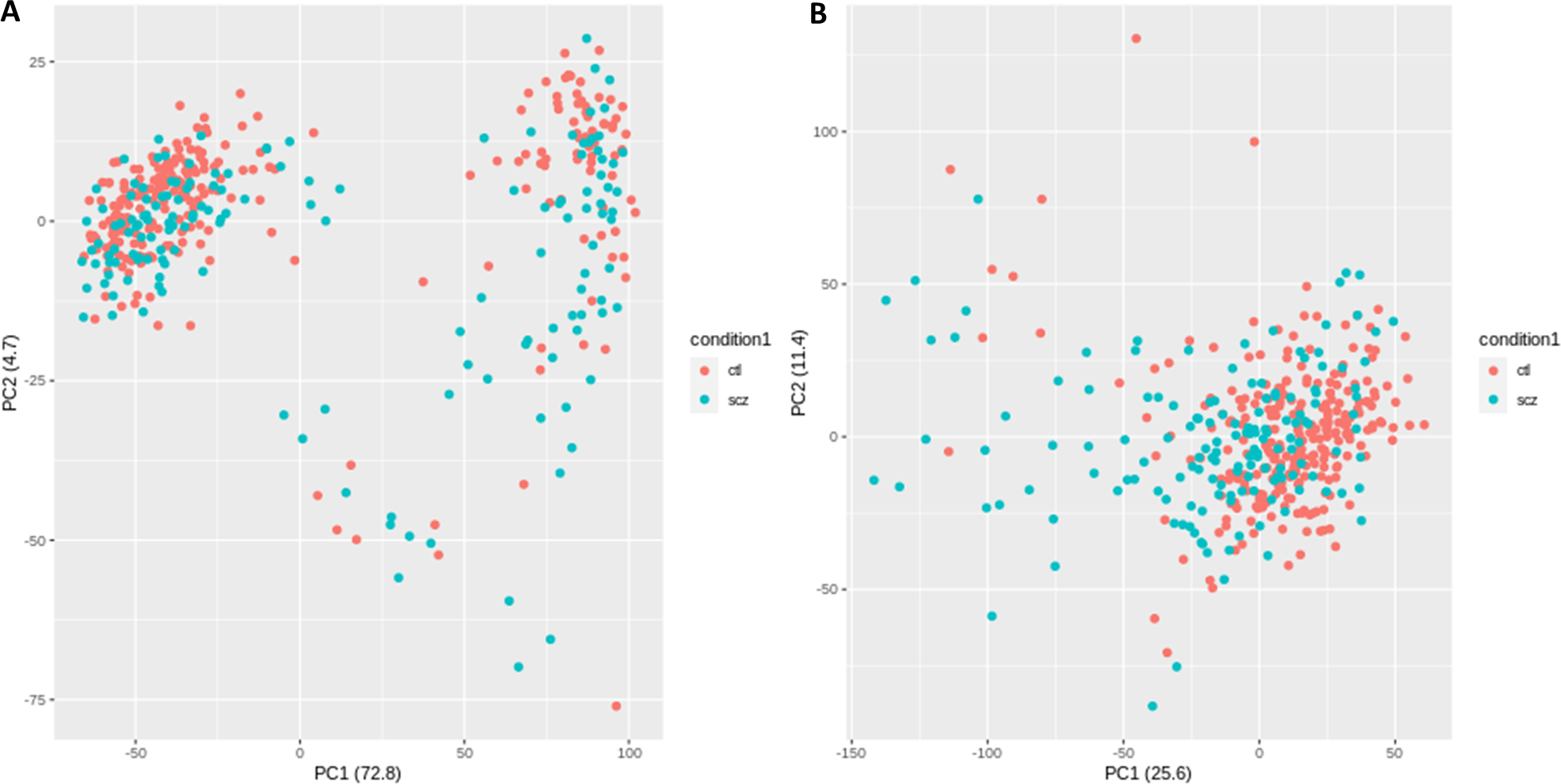

Supplement: S2 Fig — (TIF) [file pone.0346663.s002.tif]

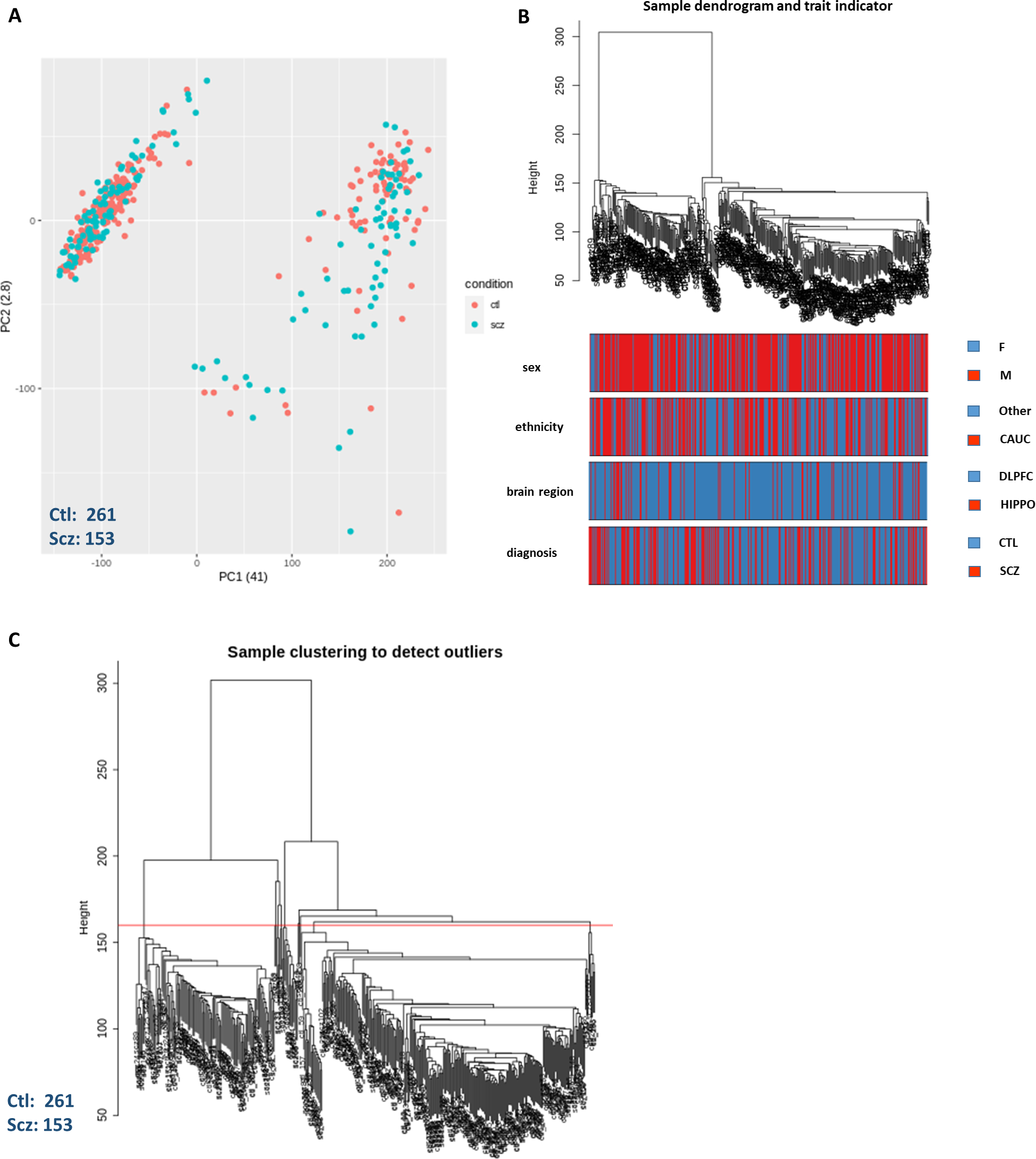

Supplement: S3 Fig — (TIF) [file pone.0346663.s003.tif]

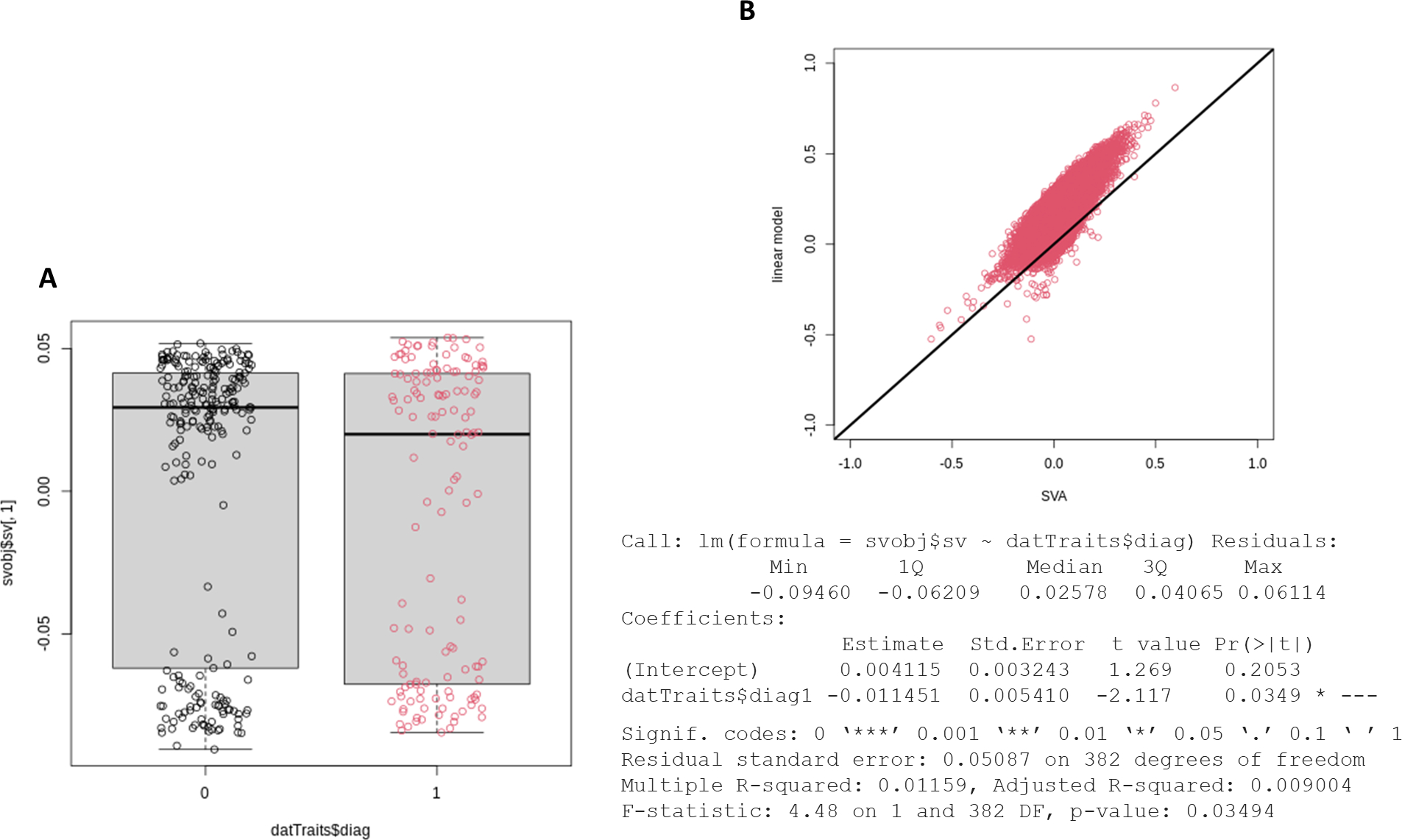

Supplement: S4 Fig — (TIF) [file pone.0346663.s004.tif]

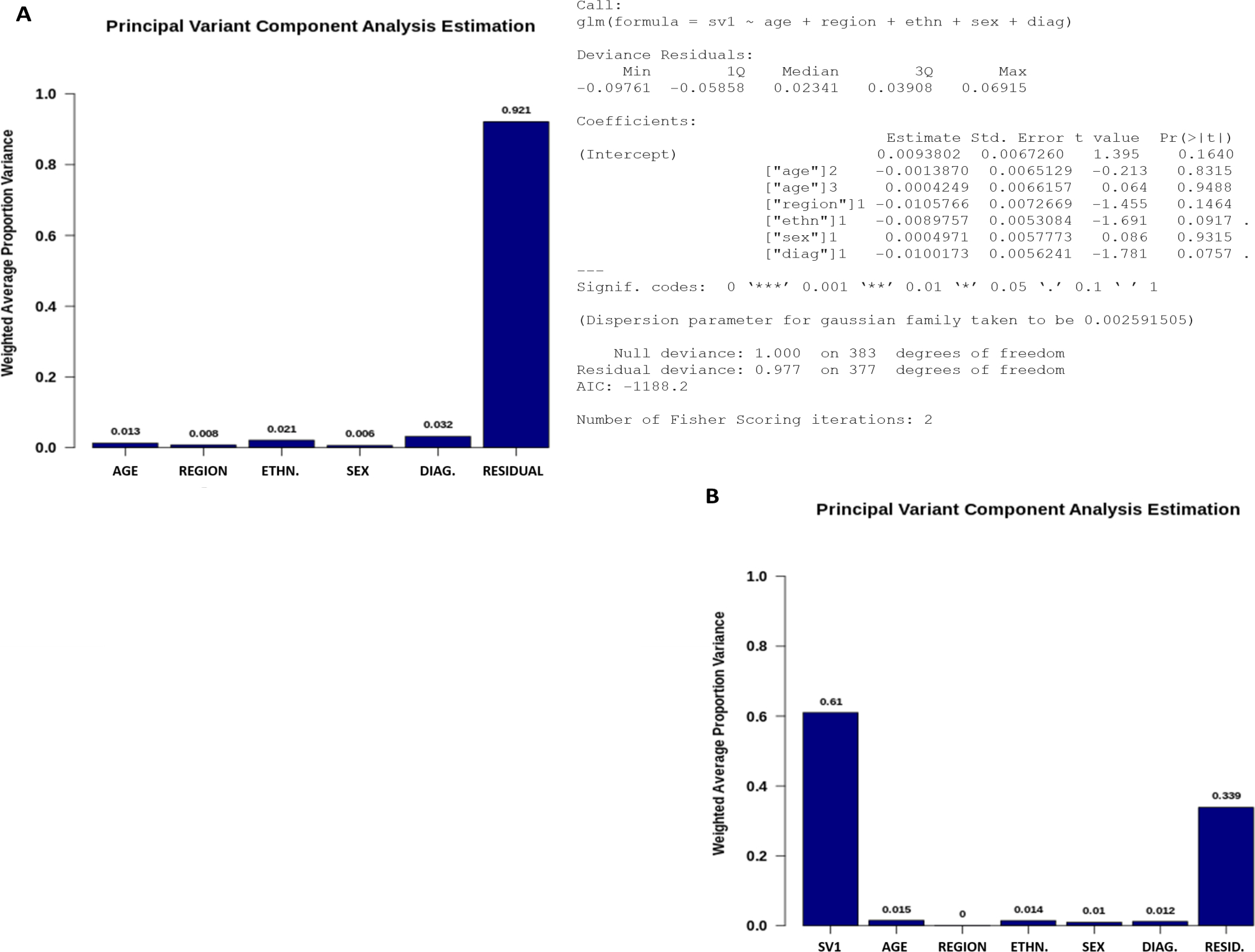

Supplement: S5 Fig — (TIF) [file pone.0346663.s005.tif]

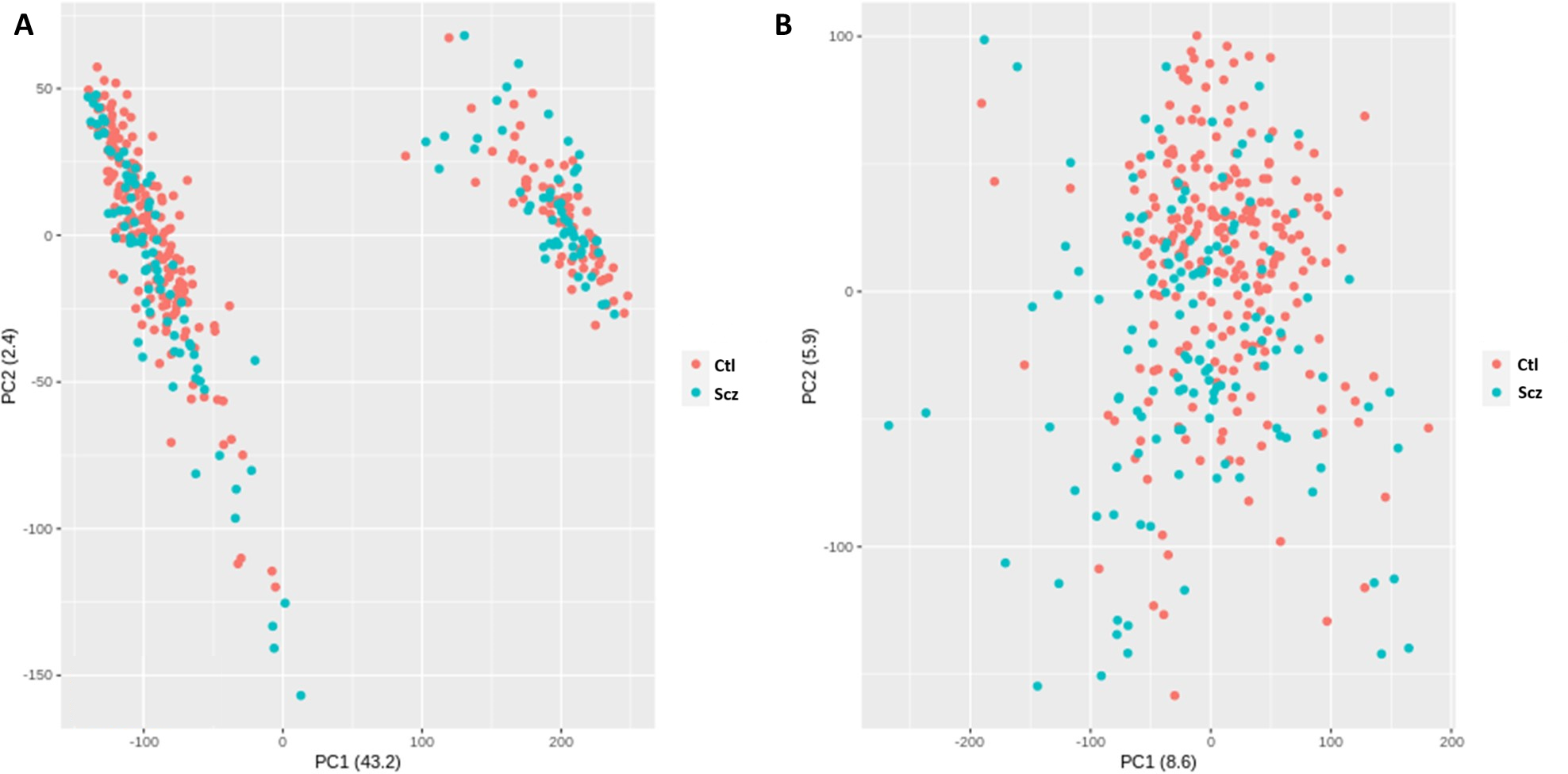

Supplement: S6 Fig — (TIF) [file pone.0346663.s006.tif]

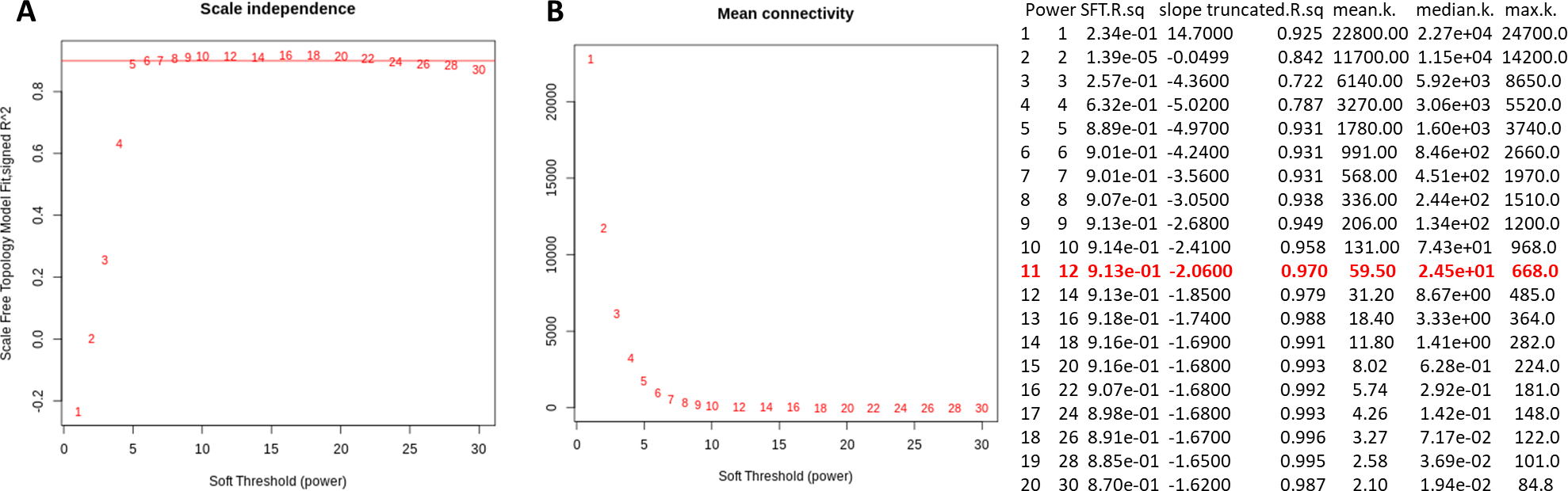

Supplement: S7 Fig — (TIF) [file pone.0346663.s007.tif]
