# Supplementary material for: Exploration of schizophrenia-associated gene modules using graph theory, co-expression networks, and dimensionality reduction
Source: PLoS One. 2026 Apr 15;21(4):e0346663. doi: 10.1371/journal.pone.0346663 (PMC13082716; doi:10.1371/journal.pone.0346663)
Supplement: S1 Code — This archive contains four distinct Jupyter Notebooks used for the analysis: SVA_Diagnostics_and_PCA_Loadings.ipynb, PCA_Variance_Driven_Reactome_Enrichment.ipynb, WGCNA_Module_Functional_Enrichment.ipynb, and igraph_MST_Topological_Centrality.ipynb. (ZIP) [file pone.0346663.s008.zip › WGCNA_Module_Functional_Enrichment.ipynb - Colab.pdf]

```
'''
Summary:
This notebook implements the core Weighted Gene Co-expression Network Analysis (WGCNA) pipeline.
After preprocessing the RNA-seq data with Surrogate Variable Analysis (SVA) and hierarchical clustering
for outlier removal, it identifies co-expression modules and correlates their eigengenes with clinical
traits (such as schizophrenia diagnosis). The defining feature of this specific notebook is its comprehensive
downstream functional annotation: it extracts the hub genes from the top WGCNA modules and uses clusterProfiler
to run comparative enrichment analyses (Gene Ontology, KEGG, and Reactome) across the different modules simultaneously.
'''
```

```
#_#_#_#_#_#_#_#_#_#_#_#_#_#_#_#_#_#_#_#_#_#_#_#_#_#_#_#_#_#_#_#_#_#_#_#_#_#_#_#_#_#_#_#_#_#_#_#_#_#_#_#_#_#_#_#_#_#_#_#_#_#_

#________________________________________________________________ANALYSIS PART A.______________________________________________

#_#_#_#_#_#_#_#_#_#_#_#_#_#_#_#_#_#_#_#_#_#_#_#_#_#_#_#_#_#_#_#_#_#_#_#_#_#_#_#_#_#_#_#_#_#_#_#_#_#_#_#_#_#_#_#_#_#_#_#_#_#_
```

```
%load_ext rpy2.ipython
from google.colab import drive
drive.mount('/content/drive')
```

```
%%R -o DER_01 -o DER_03a -o DER_03b -o DER_tmp -o capstone

if (!requireNamespace("BiocManager", quietly = TRUE))
    install.packages("BiocManager")

BiocManager::install("WGCNA")
library(WGCNA)
#BiocManager::install("zFPKM")
#library(zFPKM)
#BiocManager::install("CancerSubtypes")
#library(CancerSubtypes)
#BiocManager::install("DESeq2")
#library(DESeq2)

install.packages("tidyverse")
library(tidyverse)

capstone_tmp <- read.csv("/content/drive/My Drive/datasets/PEC_capstone_data_map_clinical.csv")
capstone <- capstone_tmp[, c(1, 2, 6, 7, 8)]

#DER_tmp <- read.delim("/content/drive/My Drive/datasets/Controls_only_PEC_Gene_expression_matrix_normalized.txt")
DER_tmp <- read.delim("/content/drive/My Drive/datasets/DER-02_PEC_Gene_expression_matrix_TPM.txt")
#DER_03_tmp <- DER_tmp[,1]
#sql_peaks_tmp <- gsub("\\.\\d+", "", DER_03_tmp)
#DER_tmp[,1] <- gsub("c(", "", sql_peaks_tmp, fixed="TRUE")

DER_01 <- read.delim("/content/drive/My Drive/datasets/DER-02_PEC_Gene_expression_matrix_TPM.txt")
#DER_01 <- read.delim("/content/drive/My Drive/datasets/Controls_only_PEC_Gene_expression_matrix_normalized.txt")
DER_03 <- DER_01[,1]
sql_peaks <- gsub("\\.\\d+", "", DER_03)
DER_01[,1] <- gsub("c(", "", sql_peaks, fixed="TRUE")
DER_03a <- DER_01[,1]    #___ gene names
DER_03b <- colnames(DER_01)[-1] #___ sample names
DER_01 <- t(scale(t(DER_01[,-1])))   #_____scaling the data(otherwise all of the highly expressed genes
                                         #will cluster together even if they have different patterns
                                         #among the samples)

```

```
import numpy                        #___________________ Divide tissues and subjects to brain/peripheral
import numpy as np                                    # and CTL/SCZ, respectively.
import pandas as pd
from sklearn.preprocessing import MinMaxScaler
from sklearn.feature_selection import VarianceThreshold
from sklearn.preprocessing import StandardScaler
from scipy.stats import gaussian_kde

%R library(data.table)
%R BrainSeq <- fread('https://raw.githubusercontent.com/LieberInstitute/brainseq_phase2/master/BrainSeq_Phase2_phenotype_c
%R head(BrainSeq)
%R E <- readRDS('/content/drive/My Drive/datasets/expression.rds')
%R gdt <- readRDS('/content/drive/My Drive/datasets/gdt.rds')
%R samp <- fread('https://storage.googleapis.com/gtex_analysis_v7/annotations/GTEx_v7_Annotations_SampleAttributesDS.txt')
```

```
%R subj <- fread('https://storage.googleapis.com/gtex_analysis_v7/annotations/GTEx_v7_Annotations_SubjectPhenotypesDS.txt'
%R samp[, SUBJID := gsub('([^-]*)-([^-]*)-.*', '\\1-\\2', SAMPID)]
%R sdt <- merge(samp, subj, by='SUBJID')
%R sdt <- merge(samp, subj, by='SUBJID')[SAMPID %in% rownames(E)]
#______________________________________________________________________________________________________________

%R -o BrainSeq
%R -o DER_01
                                                       #________________________ Check for bias due to Tissue/Sex/Race.
#1.____BrainSeq[~BrainSeq.Region.str.contains("HIPPO")]      #___DLPFC/HIPPO   # Replace each line to the line below "3.__
#2.____BrainSeq[~BrainSeq.Race.str.contains("AA")]    #___CAUC/AA
#3.____BrainSeq[~BrainSeq.Sex.str.contains("F")]    #___M/F
BrSeq = pd.DataFrame(BrainSeq)
capstone = pd.DataFrame(capstone)

DER01 = pd.DataFrame(DER_01)
DER01.columns =  DER_03b
DER01 = np.transpose(DER01)
DER01.columns =  DER_03a
DER01 = np.transpose(DER01)

#_____________________________________________________________________________I. BrainSeq DATASET(from Lieber Institute)

arr20 = np.array([])
arr20 = BrSeq['BrNum']
arr21 = np.array([])
arr21 = DER01.columns
xy21, x21_ind, y21_ind = np.intersect1d(arr20, arr21,
                                     return_indices=True)
data1 = []
for i in x21_ind:
  data1.append([BrSeq['BrNum'][i], BrSeq['Region'][i], BrSeq['Dx'][i],
                BrSeq['Sex'][i], BrSeq['Race'][i], BrSeq['RIN'][i], BrSeq['Age'][i]])
DER01_BrSeq = pd.DataFrame(data1, columns=['A', 'B', 'C', 'D', 'E', 'F', 'G'])
#DER01_BrSeq = DER01_BrSeq[~DER01_BrSeq.B.str.contains("HIPPO")]
#______________________
%R -o sdt
GTx = pd.DataFrame(sdt)

arr22 = np.array([])
arr22 = GTx['SUBJID']
xy22, x22_ind, y22_ind = np.intersect1d(arr22, arr21,
                                     return_indices=True)
data2 = []
for i in x22_ind:
  data2.append([GTx['SUBJID'][i], GTx['SMTSD'][i]])
df_GTx = pd.DataFrame(data2, columns=['A', 'B'])
#____________________________________________________
#FER01 = DER01.columns.to_series().str.contains('CMC')
#y23_ind = np.array([i for i, x in enumerate(FER01) if x])
#FER02 = DER01.columns.to_series().str.contains('Br')
#y24_ind = np.array([i for i, x in enumerate(FER02) if x])
#gtx_br = [*y23_ind, *y24_ind]

#gtx_br = [*y21_ind, *y23_ind, *y22_ind]
gtx_br = [*y21_ind, *y22_ind]

n13_train_scz = pd.DataFrame(DER01)
n14_train_scz = n13_train_scz.values[:,]
n15_train_scz = n14_train_scz[:,gtx_br]                                  #______ Filter samples

#____________________________________________________________________________II. CAPSTONE DATASET(from Kelsey)
'''
arr19a = np.array([])
arr19a = capstone['Synapse..individualID']
arr19b = np.array([])
arr19b = capstone['resource.psychencode.org..individualID']
arr21 = np.array([])
arr21 = DER01.columns
xy22, x22_ind, y22_ind = np.intersect1d(arr19b, arr21,
                                     return_indices=True)
gtx_cr = [*y22_ind]

data2 = []
for i in x22_ind:
  data2.append([capstone['resource.psychencode.org..individualID'][i],
                capstone['diagnosis'][i], capstone['sex'][i], capstone['ethnicity'][i]])
```

```
capstone_BrSeq2 = pd.DataFrame(data2, columns=['A', 'B', 'C', 'D'])

BipD = np.where(capstone_BrSeq2.B.str.contains("Bipolar Disorder"))
AutD = np.where(capstone_BrSeq2.B.str.contains("Autism Spectrum Disorder"))
AffD = np.where(capstone_BrSeq2.B.str.contains("Affective Disorder"))
BPD = np.where(capstone_BrSeq2.B.str.contains("BP"))

Dis_ind = [*BipD, *AutD, *AffD, *BPD]
Dis_ind_flat = [item for sublist in Dis_ind for item in sublist]

capstone_BrSeq2 = capstone_BrSeq2[~capstone_BrSeq2.B.str.contains("Bipolar Disorder")]
capstone_BrSeq2 = capstone_BrSeq2[~capstone_BrSeq2.B.str.contains("Autism Spectrum Disorder")]
capstone_BrSeq2 = capstone_BrSeq2[~capstone_BrSeq2.B.str.contains("Affective Disorder")]
capstone_BrSeq2 = capstone_BrSeq2[~capstone_BrSeq2.B.str.contains("BP")]

gj = []
for i in range(len(capstone_BrSeq2)):
  if len(list(capstone_BrSeq2['A'])[i])==0 :
    gj.append(i)
  if len(list(capstone_BrSeq2['B'])[i])==0 :
    gj.append(i)
  if len(list(capstone_BrSeq2['C'])[i])==0 :
    gj.append(i)
  if len(list(capstone_BrSeq2['D'])[i])==0 :
    gj.append(i)

res = []
[res.append(x) for x in gj if x not in res];

mask = np.ones(len(capstone_BrSeq2), bool)
mask[res] = False
capstone_BrSeq2_new = capstone_BrSeq2.iloc[mask]

len(capstone_BrSeq2_new)

n13_train_scz = pd.DataFrame(DER01)
n14_train_scz = n13_train_scz.values[:,]
n15_train_scz = n14_train_scz[:,gtx_cr]                                  #______ Filter samples
Dis_mask = np.ones(n15_train_scz.shape[1], bool)
Dis_mask[Dis_ind_flat] = False
n15_train_scz = n15_train_scz[:,Dis_mask]
n15_train_scz = n15_train_scz[:,mask]
'''
#________________________________________________________________________________

#n13_train_scz_min = n15_train_scz.astype(float)                          #______1. No transformation

###medianValue = median(logData);                                        #______2. Median centering data before log2-trans
###medianCtrData = logData-medianValue

n13_train_scz_min = np.log(n15_train_scz.astype(float)+1)               #______3. Log2 transformation

#n13_train_scz_min <- zFPKM(n15_train_scz.astype(float))                 #______4. Z-scale transformation (in R, from zFPK

n13_train_scz_min = np.transpose(n13_train_scz_min)                      #______ Shape the array as (samples, genes)

n13_train_scz_min[np.isnan(n13_train_scz_min)] = 0   #(57820, 414)       #______ Replace NAs with zeroes
pd.DataFrame(n13_train_scz_min).isnull().sum()

print(n13_train_scz_min.shape)
jdx = np.argwhere(np.all(n13_train_scz_min[..., :] == 0, axis=0))        #______ Remove zero columns
n13_train_scz_min = np.delete(n13_train_scz_min, jdx, axis=1)
print(n13_train_scz_min.shape)


f = VarianceThreshold().fit(n13_train_scz_min)
n13_train_scz_min = n13_train_scz_min[:, f.variances_ > 0.0]                                  #.____________________
print(n13_train_scz_min.shape)


kdx = jdx.tolist()                                                       #______ Final gene list after zero column removal
ldx = [item for sublist in kdx for item in sublist]
ER_TMM = []
DER_tmp.rename(columns = {'gene_id':'GeneName'}, inplace = True)
for i in range(len(DER_tmp['GeneName'][ldx])):
  ER_TMM.append(DER_tmp['GeneName'][ldx][i])
crr1 = np.array([])
```

```
crr1 = DER_tmp['GeneName']
crr2 = np.array([])
crr2 = ER_TMM
c22, c1_ind, c2_ind = np.intersect1d(crr1, crr2,
                     return_indices=True)
crr1[c1_ind] = 0
crr1 = [i for i in crr1 if i != 0]

crr1 = pd.DataFrame(crr1).iloc[f.variances_ > 0.0]                                               #.____________________ 2
#print(pd.DataFrame(crr1).iloc[transform.get_support(indices=True)].shape)  #____ Gene Names(columns)


'''
for i in range(n13_train_scz_min.shape[0]):                                   #______ Z-FPKM(log2) transformation (in Python)
  n13_train_tmp = n13_train_scz_min[:,i]
  kernel = gaussian_kde(n13_train_tmp)
  xi = np.linspace(n13_train_tmp.min(), n13_train_tmp.max(), 100)
  yi = kernel.evaluate(xi)
  mu = xi[np.argmax(yi)]
  U = n13_train_scz_min[n13_train_scz_min > mu].mean()
  sigma = (U - mu) * np.sqrt(np.pi / 2)
  n13_train_scz_min[:,i] = (n13_train_tmp - mu) / sigma
'''

%R -i crr1
%R -i n13_train_scz_min                                                      #______ Remove zero columns
#%R n13_train_scz_min <- n13_train_scz_min[, colSums(n13_train_scz_min != 0) > 0]
#%R print(dim(n13_train_scz_min))

#%R -i n13_train_scz_min                                                     #______ z-transformation (in R)
#zscore<- function(x){
#    z<- (x - mean(x)) / sd(x)
#    return(z)
#}
#print(dim(n13_train_scz_min))
#n13_train_scz_min <- zscore(n13_train_scz_min)
#n13_train_scz_min <- n13_train_scz_min[, colSums(n13_train_scz_min != 0) > 0]
#print(dim(n13_train_scz_min))

##    PS: The FPKM counts are already normalised but are absolutely not suitable for
##        cross-sample comparisons, i.e., not suitable for differential expression analysis.
#______________________________________________________________________________

                                                                              #_____ re-transform from log2(FPKM + z) ->
                                                                              #                              -> FPKM ->
                                                                              #                              -> zFPKM scores
#z=0.1
#exp.fpkm <- 2^expr
#exp.fpkm.original <- exp.fpkm - z
#exp.zfpkm <- zFPKM(exp.fpkm.original)
##filter out lowly expressed genes
#thres <- (ncol(exp.zfpkm) * 30) / 100
##filter all expression values that have absolute zfpkm score above 3.0 in more than 70% of the samples
#expr.zfpkm.filtered <- exp.zfpkm[(rowSums(abs(exp.zfpkm) > 3.0)) > thres, ]

#______________________________________________________________________________ Traits (CTL/SCZ)

data4 = []                                          #I. BrainSeq DATASET(from Lieber Institute)
for i in DER01_BrSeq['B']:
  if i == 'DLPFC':
    k = 0
  if i == 'HIPPO':
    k = 1
  data4.append(k)
DER01_BrSeq6 = pd.DataFrame(data4, columns=['region'])

data4 = []
for i in DER01_BrSeq['C']:
  if i == 'Control':
    k = 0
  if i == 'Schizo':
    k = 1
  data4.append(k)
DER01_BrSeq1 = pd.DataFrame(data4, columns=['diag'])

data4 = []
for i in DER01_BrSeq['D']:
```

```
  if i == 'F':
    k = 0
  if i == 'M':
    k = 1
  data4.append(k)
DER01_BrSeq2 = pd.DataFrame(data4, columns=['sex'])

data4 = []
for i in DER01_BrSeq['E']:
  if i == 'CAUC':
    k = 1
  else:
    k = 0
  data4.append(k)
DER01_BrSeq3 = pd.DataFrame(data4, columns=['ethn'])

data4 = []
for i in DER01_BrSeq['F']:
  data4.append(i)
DER01_BrSeq4 = pd.DataFrame(data4, columns=['rin'])

data4 = []
for i in DER01_BrSeq['G']:
  data4.append(i)
DER01_BrSeq5 = pd.DataFrame(data4, columns=['age'])

DER01_BrSeq_Tot = pd.concat([DER01_BrSeq1, DER01_BrSeq2, DER01_BrSeq3,
                             DER01_BrSeq4, DER01_BrSeq5, DER01_BrSeq6], axis=1)

n13_trait_path1 = []
n13_trait_path1 = pd.DataFrame(DER01_BrSeq_Tot)

n13_trait_path = n13_trait_path1
%R -i n13_trait_path

#________________________________________________________________________________
'''
                                                                                #II. CAPSTONE DATASET(from Kelsey)
#pd.DataFrame(capstone_BrSeq2_new['B']).value_counts()
#pd.DataFrame(capstone_BrSeq2_new['C']).value_counts()
#pd.DataFrame(capstone_BrSeq2_new['D']).value_counts()

data4 = []
for i in capstone_BrSeq2_new['B']:
  if i == 'Control':
    k = 0
  if i == 'Schizophrenia':
    k = 1
  data4.append(k)
DER01_BrFin1 = pd.DataFrame(data4, columns=['diag'])

data4 = []
for i in capstone_BrSeq2_new['C']:
  if i == 'F':
    k = 0
  if i == 'M':
    k = 1
  data4.append(k)
DER01_BrFin2 = pd.DataFrame(data4, columns=['sex'])

data4 = []
for i in capstone_BrSeq2_new['D']:
  if i == 'CAUC':
    k = 1
  else:
    k = 0
  data4.append(k)
DER01_BrFin3 = pd.DataFrame(data4, columns=['ethn'])

DER01_BrFin = pd.concat([DER01_BrFin1, DER01_BrFin2, DER01_BrFin3], axis=1)

n13_trait_path1 = []
n13_trait_path1 = pd.DataFrame(DER01_BrFin)

n13_trait_path = n13_trait_path1
%R -i n13_trait_path
'''
```

```

del DER01_BrSeq                                                                   #______ Clear unused variables
#del DER01_BrFin
del DER01
del n13_train_scz
del n14_train_scz
del n15_train_scz

```

```
#________________________________________________________________________________ Defining number of CTLs and SCZs
                                                                                # before removing outliers!!!!!!!!!
                                                                                # Use it as input to the PCA below


                                                                   #I. BrainSeq DATASET(from Lieber Institute)
DER01_BrSeq = pd.DataFrame(data1, columns=['A', 'B', 'C', 'D', 'E', 'F', 'G'])
#DER01_BrSeq = DER01_BrSeq[~DER01_BrSeq.B.str.contains("HIPPO")]

FER05 = DER01_BrSeq['C'].str.contains('Con')
z23_ind = np.array([i for i, x in enumerate(FER05) if x])

FER06 = DER01_BrSeq['C'].str.contains('Sc')
z24_ind = np.array([i for i, x in enumerate(FER06) if x])

gtx_all = [*z23_ind, *z24_ind]
#print(DER01_BrSeq.values[gtx_all])

n13_pca_scz_min = n13_train_scz_min[gtx_all,]
n13_pca_path = n13_trait_path.iloc[gtx_all]
#n13_pca_path = n13_trait_path['D'][gtx_all]
%R -i n13_pca_path

DER01_BrSeq['C'].value_counts()                                       #________ CTLs and SCZs

#________________________________________________________________________________
'''
                                                                    #II. CAPSTONE DATASET(from Kelsey)
FER05 = capstone_BrSeq2_new['B'].str.contains('Con')
z23_ind = np.array([i for i, x in enumerate(FER05) if x])

FER06 = capstone_BrSeq2_new['B'].str.contains('Sc')
z24_ind = np.array([i for i, x in enumerate(FER06) if x])

gtx_all = [*z23_ind, *z24_ind]
#print(DER01_BrFin1.values[gtx_all])

n13_pca_scz_min = n13_train_scz_min[gtx_all,]
n13_pca_path = n13_trait_path.iloc[gtx_all]
#n13_pca_path = n13_trait_path['diag'][gtx_all]
%R -i n13_pca_path

#DER01_BrFin['diag'].value_counts()                                       #________ CTLs and SCZs
capstone_BrSeq2_new['B'].value_counts()
'''
```

```
%%R -i n13_pca_scz_min                                                    #_____ Delete R variables, save files
                                                                               # for WGCNA analysis
rm(list=ls()[! ls() %in% c("n13_pca_scz_min", "n13_pca_path", "crr1")])
ls()

#save(n13_pca_scz_min, file = '/content/drive/My Drive/datasets/n13_pca_scz_min.RData')
#save(n13_pca_path, file = '/content/drive/My Drive/datasets/n13_pca_path.RData')

```

```
%%R -i n13_pca_scz_min
#________________________________________________________________________________ plot PCA before removing outliers!!!!!!!!!

library(ggplot2)

n13_pca_scz_min <- n13_pca_scz_min[ ,which(apply(n13_pca_scz_min, 2, var) != 0)]#_Remove zero variance columns from the new
genes=paste("gene",seq(1:dim(n13_pca_scz_min)[2]), sep="")
colnames(n13_pca_scz_min)=genes
row.names(n13_pca_scz_min)=c(paste0("ctl_",seq(1:261)), paste0("scz",seq(1:153)))  #___Python command: DER01_BrSeq['C'].val
condition1=rep(c("ctl"), each=261)
condition2=rep(c("scz"), each=153)
```

```
condition1 <- append(condition1, condition2)
length(condition1)

#pca_data=prcomp(n13_train_scz_min, center = TRUE, scale = TRUE)
#pca_data_perc=round(100*pca_data$sdev^2/sum(pca_data$sdev^2),1)
#df_pca_data=data.frame(PC1 = pca_data$x[,1], PC2 = pca_data$x[,2], sample = row.names(n13_train_scz_min), condition1=condi
#ggplot(df_pca_data, aes(PC1,PC2, color = condition1))+
#      geom_point(size=8)+
#      labs(x=paste0("PC1 (",pca_data_perc[1],")"), y=paste0("PC2 (",pca_data_perc[2],")"))

                                                                           #____ PCA for Z-scaled data
pca_data=prcomp(n13_pca_scz_min, center = TRUE, scale = TRUE)
pca_data_perc=round(100*pca_data$sdev^2/sum(pca_data$sdev^2),1)
df_pca_data=data.frame(PC1 = pca_data$x[,1], PC2 = pca_data$x[,2], sample = row.names(n13_pca_scz_min), condition1=conditic

                                                                   #____________ Plot samples
ggplot(df_pca_data, aes(PC1,PC2, color = condition1))+
      geom_point(size=2)+
      labs(x=paste0("PC1 (",pca_data_perc[1],")"), y=paste0("PC2 (",pca_data_perc[2],")"))

                                                                   #____________ Plot genes
#ggplot(df_pca_data, aes(PC1,PC2, color = sample))+geom_point(size=2)+
#      labs(x=paste0("PC1 (",pca_data_perc[1],")"), y=paste0("PC2 (",pca_data_perc[2],")"))+
#      theme(legend.position = "none")

#df_pca_data[df_pca_data$PC1>300000,]                                      #____ How many patients/controls
#sum(df_pca_data$PC1>300000)                                                   #  as outliers in the PC1 axis

#_______________________________________________________________________________ PCA using FactoMineR package

##install.packages("FactoMineR")
##library(FactoMineR)
##install.packages("factoextra")
##library(factoextra)
#pca_data <- as.data.frame(n13_train_scz_min)
#pca_data$group <- c(rep('ctl',261),rep('scz',153))
#pca <- PCA(pca_data[,1:(ncol(pca_data)-1)], graph = F)
#fviz_pca_ind(pca,
#         geom.ind = "point",
#         col.ind = pca_data$group,
#         palette = c("#00AFBB", "#E7B800"),
#         addEllipses = TRUE,
#         legend.title = "Groups")

```

```
#___________________________________WGCNA_______________________________________
```

```
#___________________________________WGCNA_______________________________________
```

```
#___________________________________WGCNA_______________________________________
```

```
%%R                                              #_____________________________ this calculates the whole network connecti

Aadj = adjacency(t(n13_pca_scz_min), type = "distance")
# this calculates the whole network connectivity
k = as.numeric(apply(Aadj, 2, sum)) - 1
# standardized connectivity
Z.k = scale(k)
```

```
%%R

sampleTree = hclust(dist(n13_pca_scz_min), method = "average");
par(cex = 0.6);
par(mar = c(0,4,2,0))
plot(sampleTree, main = "Sample clustering to detect outliers",
     sub="", xlab="", cex.lab = 1.5,cex.axis = 1.5, cex.main = 2)

abline(h = 160, col = "red");
clust = cutreeStatic(sampleTree, cutHeight = 160, minSize = 1)
table(clust)
keepSamples1 = (clust==1)
print(sum(keepSamples1))
keepSamples2 = (clust==2)
print(sum(keepSamples2))
```

```

keepSamples <- list()
keepSamples <- c(keepSamples, keepSamples1+keepSamples2)
length(keepSamples)

dim(n13_pca_scz_min)
datExpr = n13_pca_scz_min[which(keepSamples != 0),]
print(dim(datExpr))

nGenes <- ncol(datExpr)
nSamples <- nrow(datExpr)

datTraits <- n13_pca_path[which(keepSamples != 0),]
print(dim(datTraits))

```

```
%%R

table(clust)

```

```
%%R                                             #_____________________________ this calculates the whole network connecti

Badj = adjacency(t(datExpr), type = "distance")
k = as.numeric(apply(Badj, 2, sum)) - 1
# standardized connectivity
Z.k = scale(k)

```

```
%%R                                       #___________________________________A. Run the codes separately to create plots
                                                                              # Add each one of these plots beneath th
# Setting the colors to match the regional colors
region_labels <- RColorBrewer::brewer.pal(6, "Set1")[1:3]

datTraits <- n13_pca_path[which(keepSamples != 0),]

# Making sure the regional colors match what has been used througout and making a column with the hex codes.

datTraits <- datTraits %>%
  mutate(cell.names = row.names(datTraits)) %>%
  mutate(x = ifelse(datTraits$sex == "1", region_labels[1],
                    ifelse(datTraits$sex == "0", region_labels[2], NA)))

# Re-performing heirarchial clustering
sampleTree2 <- hclust(dist(datExpr), method = "average")

# Writing out that dendrogram to a PDF
#pdf(file = file.path(file_dir, "sample.dendrogram_region.heatmap.pdf"), height = 8, width = 12)
plotDendroAndColors(sampleTree2, datTraits$x,
                groupLabels = names(datTraits),
                main = "Sample dendrogram and sex")
#dev.off()

```

```
%%R                                       #___________________________________B.

datTraits <- datTraits %>%
  mutate(cell.names = row.names(datTraits)) %>%
  mutate(x = ifelse(datTraits$ethn == "1", region_labels[1],
                    ifelse(datTraits$ethn == "0", region_labels[2], NA)))

# Writing out that dendrogram to a PDF
#pdf(file = file.path(file_dir, "sample.dendrogram_region.heatmap.pdf"), height = 8, width = 12)
plotDendroAndColors(sampleTree2, datTraits$x,
                groupLabels = names(datTraits),
                main = "Sample dendrogram and ethnicity")

```

```
%%R                                       #___________________________________C.

datTraits <- datTraits %>%
  mutate(cell.names = row.names(datTraits)) %>%
  mutate(x = ifelse(datTraits$region == "1", region_labels[1],
                    ifelse(datTraits$region == "0", region_labels[2], NA)))
```

```

# Writing out that dendrogram to a PDF
#pdf(file = file.path(file_dir, "sample.dendrogram_region.heatmap.pdf"), height = 8, width = 12)
plotDendroAndColors(sampleTree2, datTraits$x,
                groupLabels = names(datTraits),
                main = "Sample dendrogram and region")

```

```
%%R                                         #____________________________________D.

datTraits <- datTraits %>%
  mutate(cell.names = row.names(datTraits)) %>%
  mutate(x = ifelse(datTraits$diag == "1", region_labels[1],
                    ifelse(datTraits$diag == "0", region_labels[2], NA)))

# Writing out that dendrogram to a PDF
#pdf(file = file.path(file_dir, "sample.dendrogram_region.heatmap.pdf"), height = 8, width = 12)
plotDendroAndColors(sampleTree2, datTraits$x,
                groupLabels = names(datTraits),
                main = "Sample dendrogram and diagnosis")

```

```
%%R                                         #____________________________________E.

xs=quantile(datTraits$age,c(0,1/3,2/3,1))
xs[1]=xs[1]-.00005
datTraits <- datTraits %>% mutate(category=cut(age, breaks=xs,
  labels=c("low","middle","high"),ordered_result = TRUE))
boxplot(datTraits$age~datTraits$category,col=3:5)

```

```
#________________________________________________________________________________ defining new number of CTLs and SCZs after
                                                                                 # Use it as input to the PCA below

                                            #I. BrainSeq DATASET(from Lieber Institute)
#DER01_BrSeq = pd.DataFrame(data1, columns=['A', 'B', 'C', 'D', 'E', 'F', 'G'])
#%R -i DER01_BrSeq
#%R -o DER02_BrSeq DER02_BrSeq <- DER01_BrSeq[which(keepSamples != 0),]
#pd.DataFrame(DER02_BrSeq['C']).value_counts()

%R -o datTraits
print(pd.DataFrame(datTraits['diag']).value_counts())

'''
                                            #II. CAPSTONE DATASET(from Kelsey)
%R -i capstone_BrSeq2_new
%R -o capstone_BrSeq3_new capstone_BrSeq3_new <- capstone_BrSeq2_new[which(keepSamples != 0),]
pd.DataFrame(capstone_BrSeq3_new['B']).value_counts()
'''
```

```
%%R
#________________________________________________________________________________ plot PCA after removing outliers!!!!!!!!!!

library(ggplot2)

crr1 <- crr1[which(apply(datExpr, 2, var) != 0),]
datExpr <- datExpr[ ,which(apply(datExpr, 2, var) != 0)]#_Remove zero variance columns from the new dataset
save(datExpr, file = '/content/drive/My Drive/datasets/datExpr.RData')
save(crr1, file = '/content/drive/My Drive/datasets/crr1.RData')

genes=paste("gene",seq(1:dim(datExpr)[2]), sep="")
colnames(datExpr)=genes
row.names(datExpr)=c(paste0("ctl_",seq(1:246)), paste0("scz",seq(1:138)))  #___Python command: DER01_BrSeq['C'].value_count
condition1=rep(c("ctl"), each=246)
condition2=rep(c("scz"), each=138)
condition1 <- append(condition1, condition2)
length(condition1)

#pca_data=prcomp(datExpr, center = TRUE, scale = TRUE)
#pca_data_perc=round(100*pca_data$sdev^2/sum(pca_data$sdev^2),1)
#df_pca_data=data.frame(PC1 = pca_data$x[,1], PC2 = pca_data$x[,2], sample = row.names(datExpr), condition1=condition1)
#ggplot(df_pca_data, aes(PC1,PC2, color = condition1))+
#      geom_point(size=8)+
#      labs(x=paste0("PC1 (",pca_data_perc[1],")"), y=paste0("PC2 (",pca_data_perc[2],")"))
```

```
                                                                           #____ PCA for Z-scaled data
pca_data=prcomp(datExpr, center = TRUE, scale = TRUE)
pca_data_perc=round(100*pca_data$sdev^2/sum(pca_data$sdev^2),1)
df_pca_data=data.frame(PC1 = pca_data$x[,1], PC2 = pca_data$x[,2], sample = row.names(datExpr), condition1=condition1)

                                                       #_____________Plot samples
ggplot(df_pca_data, aes(PC1,PC2, color = condition1))+
      geom_point(size=2)+
      labs(x=paste0("PC1 (",pca_data_perc[1],")"), y=paste0("PC2 (",pca_data_perc[2],")"))

                                                       #_____________Plot genes
#ggplot(df_pca_data, aes(PC1,PC2, color = sample))+geom_point(size=2)+
#      labs(x=paste0("PC1 (",pca_data_perc[1],")"), y=paste0("PC2 (",pca_data_perc[2],")"))+
#      theme(legend.position = "none")
#_______________________________________________________________________________ PCA using FactoMineR package

##install.packages("FactoMineR")
##library(FactoMineR)
##install.packages("factoextra")
##library(factoextra)
#pca_data <- as.data.frame(datExpr)
#pca_data$group <- c(rep('ctl',250),rep('scz',139))
#pca <- PCA(pca_data[,1:(ncol(pca_data)-1)], graph = F)
#fviz_pca_ind(pca,
#         geom.ind = "point",
#         col.ind = pca_data$group,
#         palette = c("#00AFBB", "#E7B800"),
#         addEllipses = TRUE,
#         legend.title = "Groups")

```

```
%%R
#_______________________________________________________________________________ Matrix factorization

#load(file = '/content/drive/My Drive/datasets/datExpr.RData')
#load(file = '/content/drive/My Drive/datasets/datTraits.RData')
#load(file = '/content/drive/My Drive/datasets/crr1.RData')

datExpr = t(datExpr)
colnames(datExpr) <- c(rownames(datTraits ))
rownames(datExpr) <- t(crr1)
datTraits$diag <- as.factor(datTraits$diag)
datTraits$ethn <- as.factor(datTraits$ethn)
datTraits$sex <- as.factor(datTraits$sex)
datTraits$age <- as.factor(datTraits$category)
datTraits$region <- as.factor(datTraits$region)
datExpr = t(datExpr)

```

```
%%R                                     #______________________________________ SVA analysis


if (!requireNamespace('BiocManager', quietly = TRUE))
    install.packages('BiocManager')

BiocManager::install('sva')
library(sva)

mod0 = model.matrix(~as.factor(ethn)+as.factor(sex)+as.factor(age)+as.factor(region), data=datTraits)
mod = model.matrix(~as.factor(diag)+as.factor(ethn)+as.factor(sex)+as.factor(age)+as.factor(region), data=datTraits)
n.sv = num.sv(t(datExpr),mod,method="leek")
svobj = sva(t(datExpr),mod,mod0,n.sv=n.sv)

cleanY = function(y, mod, svobj) {
    X = cbind(mod, svobj)
    Hat = solve(t(X) %*% X) %*% t(X)
    beta = (Hat %*% t(y))
    rm(Hat)
    gc()
    P = ncol(mod)
    return(y - t(as.matrix(X[,-c(1:P)]) %*% beta[-c(1:P),]))
}

datExpr_cln <- cleanY(t(datExpr), mod, svobj$sv)

```

```
#_____________________________________________________________________________________________________
#mod0 = model.matrix(~as.factor(Gender)+as.factor(Population), data=pheno)
#mod = model.matrix(~as.factor(inversion_genotype)+as.factor(Gender)+as.factor(Population), data=pheno)
#n.sv = num.sv(edata,mod,method="leek")
#svobj = sva(edata,mod,mod0,n.sv=n.sv)

```

```
%%R
#______________________________________________________________________________ plot PCA after SVA analysis!!!!!!!!!

datExpr_cln <- t(datExpr_cln)

library(ggplot2)

#crr1 <- crr1[which(apply(datExpr_cln, 2, var) != 0),]
#datExpr_cln <- datExpr_cln[ ,which(apply(datExpr_cln, 2, var) != 0)]#_Remove zero variance columns from the new dataset

genes=paste("gene",seq(1:dim(datExpr_cln)[2]), sep="")
colnames(datExpr_cln)=genes
row.names(datExpr_cln)=c(paste0("ctl_",seq(1:246)), paste0("scz",seq(1:138)))  #___Python command: DER01_BrSeq['C'].value_c
condition1=rep(c("ctl"), each=246)
condition2=rep(c("scz"), each=138)
condition1 <- append(condition1, condition2)
length(condition1)
                                                                            #____ PCA for Z-scaled data
pca_data=prcomp(datExpr_cln, center = TRUE, scale = TRUE)
pca_data_perc=round(100*pca_data$sdev^2/sum(pca_data$sdev^2),1)
df_pca_data=data.frame(PC1 = pca_data$x[,1], PC2 = pca_data$x[,2], sample = row.names(datExpr_cln), condition1=condition1)

                                                        #_____________Plot samples
ggplot(df_pca_data, aes(PC1,PC2, color = condition1))+
      geom_point(size=2)+
      labs(x=paste0("PC1 (",pca_data_perc[1],")"), y=paste0("PC2 (",pca_data_perc[2],")"))

```

```
%%R

library(devtools)
install_github("vqv/ggbiplot")
library(ggbiplot)
library(ggplot2)

# Plot the PCA plots
hvPCA1<-ggbiplot(pca_data,choices=c(1,2),scale=0,groups=datTraits$diag, ellipse=T,var.axes=F) + scale_color_manual(values=c
hvPCA2<-ggbiplot(pca_data,choices=c(1,2),scale=0,groups=datTraits$ethn, ellipse=T,var.axes=F) + scale_color_manual(values=c
hvPCA3<-ggbiplot(pca_data,choices=c(1,2),scale=0,groups=datTraits$sex, ellipse=T,var.axes=F) + scale_color_manual(values=c(
hvPCA4<-ggbiplot(pca_data,choices=c(1,2),scale=0,groups=datTraits$region, ellipse=T,var.axes=F) + scale_color_manual(values
hvPCA5<-ggbiplot(pca_data,choices=c(1,2),scale=0,groups=datTraits$age, ellipse=T,var.axes=F) + scale_color_manual(values=c(

```

```
%%R

# Show the diagnosis plot
hvPCA1
```

```
%%R

# Show the ethnicity plot
hvPCA2
```

```
%%R

# Show the sex plot
hvPCA3
```

```
%%R

# Show the region plot
hvPCA4
```

```
%%R

```

```
# Show the age plot
hvPCA5
```

```
%%R

datExpr <- datExpr_cln
datExpr = t(datExpr)
colnames(datExpr) <- c(rownames(datTraits ))
rownames(datExpr) <- t(crr1)
datTraits$diag <- as.factor(datTraits$diag)
datTraits$ethn <- as.factor(datTraits$ethn)
datTraits$sex <- as.factor(datTraits$sex)
datTraits$age <- as.factor(datTraits$category)
datTraits$region <- as.factor(datTraits$region)
datExpr = t(datExpr)

```

```
%%R                                               #_____________________________ this calculates the whole network connecti

Cadj = adjacency(t(datExpr), type = "distance")
k = as.numeric(apply(Cadj, 2, sum)) - 1
# standardized connectivity
Z.k = scale(k)

```

```
%%R

# Setting what soft thresholds (powers) should be tested for potential use as a soft threshold
powers <- c(c(1:10), seq(from = 12, to=30, by=2))

# Calculating soft thresholds for a signed network
#sft <- pickSoftThreshold(datExpr, powerVector = powers,
#                         verbose = 5,corFnc="bicor",
#                         corOptions = list(use = 'p', maxPOutliers = 0.05),
#                         networkType ="signed hybrid",
#                         moreNetworkConcepts = F)

# Calculating soft thresholds for a signed network
sft <- pickSoftThreshold(datExpr, powerVector = powers,
                        verbose = 5, corFnc="cor",
                        corOptions = list(use = 'p'),
                        networkType ="signed")
```

```
%%R

# Plotting the soft threshold diagnostics in order to choose an optimal soft threshold.
# Setting display margins
par(mfrow = c(1,1))
cex1 = 0.9

# Scale-free topology fit index as a function of the soft-thresholding power. We are looking to choose a soft threshold whe
plot(sft$fitIndices[,1], -sign(sft$fitIndices[,3])*sft$fitIndices[,2],
     xlab="Soft Threshold (power)",ylab="Scale Free Topology Model Fit,signed R^2",type="n",
     main = paste("Scale independence"))
text(sft$fitIndices[,1], -sign(sft$fitIndices[,3])*sft$fitIndices[,2],
labels=powers,cex=cex1,col="red")
abline(h=0.9,col="red") # this line corresponds to using an R^2 cut-off of h that could be used as a guide to picking a sof
```

```
%%R

# Mean connectivity as a function of the soft-thresholding power. Again, we are looking at when the plot begins leveling ou
plot(sft$fitIndices[,1], sft$fitIndices[,5],
xlab="Soft Threshold (power)",ylab="Mean Connectivity", type="n",
main = paste("Mean connectivity"))
text(sft$fitIndices[,1], sft$fitIndices[,5], labels=powers, cex=cex1,col="red")
```

```




#______________________________________________ Additional PCA analysis


```

```
%%R
#_______________________________________________________________________________ Additional PCA analysis

#load(file = '/content/drive/My Drive/datasets/m13_train_scz_min')
#load(file = '/content/drive/My Drive/datasets/m13_trait_path.RData')
#load(file = '/content/drive/My Drive/datasets/crr1.RData')

#m13_train_scz_min = t(m13_train_scz_min)
#colnames(m13_train_scz_min) <- c(rownames(m13_trait_path))
#rownames(m13_train_scz_min) <- t(crr1)
#m13_trait_path$diag <- as.factor(m13_trait_path$diag)
#m13_train_scz_min = t(m13_train_scz_min)
#_______________________________________________________________________________________ PRCOMP (& randomForest prediction)

install.packages("randomForest")
install.packages("caret")
library(caret)
library(randomForest)

colnames(datExpr) <- t(crr1)
#datTraits$diag <- as.factor(datTraits$diag)

set.seed(1234)
new_train_scz_min <- datExpr[sample(nrow(datExpr)),]
new_trait_path <- datTraits[sample(nrow(datTraits)),]
new_trait_path <- new_trait_path[match(rownames(new_train_scz_min), rownames(new_trait_path)),]
set.seed(NULL)

pca.train <- new_train_scz_min[1:(as.integer(0.7*nrow(new_train_scz_min))),]
pca.test <- new_train_scz_min[-(1:(as.integer(0.7*nrow(new_train_scz_min)))),]
pca_trait_train <- new_trait_path[1:(as.integer(0.7*nrow(new_trait_path))),]
pca_trait_test <- new_trait_path[-(1:(as.integer(0.7*nrow(new_trait_path)))),]
pca.test <- pca.test[ ,which(apply(pca.train, 2, var) != 0)]
pca.train <- pca.train[ ,which(apply(pca.train, 2, var) != 0)]
prin_comp <- prcomp(pca.train, scale. = T)

#std_dev <- prin_comp$sdev                                   #___________ Plots
#pr_var <- std_dev^2
#prop_varex <- pr_var/sum(pr_var)

#plot(prop_varex, xlab = "Principal Component",
#             ylab = "Proportion of Variance Explained",
#             type = "b")

#plot(cumsum(prop_varex), xlab = "Principal Component",
#              ylab = "Cumulative Proportion of Variance Explained",
#              type = "b")

                                                            #______randomForest
train.data <- data.frame(diag = pca_trait_train$diag, prin_comp$x)
train.data <- train.data[,1:31]

RFmodel_Fit <- randomForest(diag ~ .,data = train.data, method = "anova")
RFmodel_Fit
test.data <- predict(prin_comp, newdata = pca.test)
test.data <- as.data.frame(test.data)
test.data <- test.data[,1:30]
RFmodel_Fit.prediction <- predict(RFmodel_Fit, test.data)
print(confusionMatrix(RFmodel_Fit.prediction, pca_trait_test$diag)$overall[1])
print(RFmodel_Fit$confusion)

#_______________________________________________________________________________________ PCATOOLS

if (!requireNamespace('BiocManager', quietly = TRUE))
    install.packages('BiocManager')

BiocManager::install('PCAtools')
library(PCAtools)

#______________________________________________________ PCATOOLS(various Plotloadings and ENSEMBL to SYMBOL translation)

organism = "org.Hs.eg.db"
```

```
BiocManager::install(organism, character.only = TRUE, force = TRUE)
library(organism, character.only = TRUE)

require(org.Hs.eg.db)
mapping <- mapIds(
  org.Hs.eg.db,
  keys = colnames(datExpr),
  column = 'SYMBOL',
  keytype = 'ENSEMBL')
colnames(datExpr) <- make.unique(ifelse(is.na(mapping), colnames(datExpr), mapping))

p <- pca(t(datExpr), metadata = datTraits, center = TRUE,
         scale = TRUE, removeVar = 0.0)
```

```
%%R

biplot(p, showLoadings = TRUE, labSize = 5, pointSize = 5, sizeLoadingsNames = 5)      #_ 1.
```

```
%%R

pairsplot(p)                                                                            #_ 2.
```

```
%%R

eigencorplot(p,                                                                         #_ 3.
components = getComponents(p, 1:5),
metavars = c("diag", "sex", "ethn", "age"),
col = c('darkblue', 'blue2', 'black', 'red2', 'darkred'),
cexCorval = 0.7,
colCorval = 'white',
fontCorval = 2,
posLab = 'bottomleft',
rotLabX = 45,
posColKey = 'top',
cexLabColKey = 1.5,
scale = TRUE,
main = bquote(Principal ~ component ~ Pearson ~ r^2 ~ clinical ~ correlates),
plotRsquared = TRUE,
corFUN = 'pearson',
corUSE = 'pairwise.complete.obs',
corMultipleTestCorrection = 'BH',
signifSymbols = c('****', '***', '**', '*', ''),
signifCutpoints = c(0, 0.0001, 0.001, 0.01, 0.05, 1))
```

```
%%R

plotloadings(p,                                                                          #_ 4a.
    components = getComponents(p, c(1)),
    rangeRetain = 0.01, absolute = F,
    drawConnectors = TRUE, labSize = 4) + coord_flip()
```

```
%%R

plotloadings(p,                                                                          #_ 4b.
    components = getComponents(p, c(2)),
    rangeRetain = 0.01, absolute = F,
    drawConnectors = TRUE, labSize = 4) + coord_flip()
```

```
%%R

plotloadings(p,                                                                          #_ 4c.
    components = getComponents(p, c(3)),
    rangeRetain = 0.01, absolute = F,
    drawConnectors = TRUE, labSize = 4) + coord_flip()
```

```
%%R

```

```
plotloadings(p,                                                                   #_ 4d.
    components = getComponents(p, c(4)),
    rangeRetain = 0.01, absolute = F,
    drawConnectors = TRUE, labSize = 4) + coord_flip()
```

```
%%R

plotloadings(p,                                                                   #_ 4e.
    components = getComponents(p, c(5)),
    rangeRetain = 0.01, absolute = F,
    drawConnectors = TRUE, labSize = 4) + coord_flip()
```

```
%%R

plotloadings(p,                                                                   #_ 5.
    components = getComponents(p, c(1,2,3,4)),
    rangeRetain = 0.1,
    labSize = 3.0,
    absolute = FALSE,
    title = 'Loadings plot',
    subtitle = 'Misc PCs',
    caption = 'Top 10% variables',
    shape = 23, shapeSizeRange = c(1, 16),
    drawConnectors = FALSE)

```

```
%%R

plotloadings(p,                                                                   #_ 6.
components = getComponents(p, c(1,2,3,4,5)),
rangeRetain = 0.001,
labSize = 3.0,
title = 'Loadings plot',
subtitle = 'PC1, PC2, PC3, PC4, PC5',
caption = 'Top 1% variables',
drawConnectors = TRUE)

```

```
%%R

prin_comp <- prcomp(datExpr, scale. = F)
expl_var <- prin_comp$sdev^2/sum(prin_comp$sdev^2)
barplot(expl_var[1:50], ylab="Variance",
        names.arg=paste0("PC",seq(1:50)), col="darkred")

N_perm <- 10
expl_var_perm <- matrix(NA, ncol = length(prin_comp$sdev), nrow = N_perm)
for(k in 1:N_perm)
{
  expr_perm <- apply(datExpr,2,sample)
  PC_perm <- prcomp(expr_perm, center=TRUE, scale=FALSE)
  expl_var_perm[k,] <- PC_perm$sdev^2/sum(PC_perm$sdev^2)
}
plot(expl_var[1:200]~seq(1:200), ylab="Variance",
     col="green", type='o', xlab="PC")
lines(colMeans(expl_var_perm)[1:200]~seq(1:200),col="red")
legend("topright", c("original data", "data permutation"),
       fill=c("green","red"), inset=0.02)

pval <- apply(t(expl_var_perm) >= expl_var,1,sum) / N_perm
plot(pval[1:200]~seq(1:200),col="darkred",type='o',
     xlab="PC",ylab="p-value")
optPC<-head(which(pval>=0.05),1)-1
mtext(paste0("OPTIMAL PC = ",optPC))
```

```
%%R

install.packages("tsne")
##install.packages("Rtsne")
library(tsne)
##library(Rtsne)
library(ggplot2)

nComponents <- 48
```

```
seed <- 1234
set.seed(seed)

dat.filter.BCV.tsne<-tsne(pca_data$x[,1:nComponents],perplexity=210,max_iter=2000,whiten = FALSE)

tsne_plot <- data.frame(x = dat.filter.BCV.tsne[,1], y = dat.filter.BCV.tsne[,2], col = datTraits$diag)
ggplot(tsne_plot) + geom_point(aes(x=x, y=y, color=col, size=1))
```

```
%%R
'''
if (!requireNamespace('BiocManager', quietly = TRUE))
    install.packages('BiocManager')

BiocManager::install('PCAtools')
library(PCAtools)

#colnames(datExpr) <- c(rownames(datTraits ))
datExpr = t(datExpr)
p <- pca(datExpr, metadata = datTraits, center = TRUE,                  #_ 1.
         scale = TRUE)

elbow <- findElbowPoint(p$variance)

screeplot(p,components = getComponents(p, 1:20),vline = c(elbow)) +
    geom_label(aes(x = elbow + 1, y = 50,
      label = 'Elbow method', vjust = -1, size = 8))

datExpr = t(datExpr)
'''
```

```
%%R
'''
elbow <- findElbowPoint(p$variance)
screeplot(p,components = getComponents(p, 1:20),vline = c(elbow)) +
    geom_label(aes(x = elbow + 1, y = 50,
      label = 'Elbow method', vjust = -1, size = 8))
'''
```

```
%%R
'''
install.packages("tsne")
##install.packages("Rtsne")
library(tsne)
##library(Rtsne)
library(ggplot2)

nComponents <- elbow
seed <- 1234
set.seed(seed)

dat.filter.BCV.tsne<-tsne(pca_data$x[,1:nComponents],perplexity=210,max_iter=2000,whiten = FALSE)

tsne_plot <- data.frame(x = dat.filter.BCV.tsne[,1], y = dat.filter.BCV.tsne[,2], col = datTraits$diag)
ggplot(tsne_plot) + geom_point(aes(x=x, y=y, color=col, size=1))
'''
```

```




#______________________________________________ Additional PCA analysis




```

```
#- - - - - - - - - - - - - - - - - - - - - - - - - - - - - - - - - - - - - - - - - - - - - - - - - - - - - - - - - - - - - - - - - - - - - - - - - - - -
```

```
#- - - - - - - - - - - - - - - - - - - - - - - - - - - - - - - - - - - - - - - - - - - - - - - - - - - - - - - - - - - - - - - - - - - - - - - - - - - -
```

```
#- - - - - - - - - - - - - - - - - - - - - - - - - - - - - - - - - - - - - - - - - - - - - - - - - - - - - - - - - - - - - - - - - - - - - - - - - - - -
```

```
%%R

BiocManager::install("WGCNA")
library(WGCNA)

cor <- WGCNA::cor

net = blockwiseModules(datExpr, power = 12,
TOMType = "signed", minModuleSize = 50, deepSplit = 4,
reassignThreshold = 0, mergeCutHeight = 0.1,
numericLabels = TRUE, pamRespectsDendro = FALSE,
saveTOMs = FALSE, maxBlockSize = 16000,networkType = "signed",
saveTOMFileBase = "femaleMouseTOM",
verbose = 3)

#net = blockwiseModules(datExpr, power = 12,
#TOMType = "signed", minModuleSize = 30, corType = "bicor",
#reassignThreshold = 0, mergeCutHeight = 0.1, deepSplit = 4,
#numericLabels = TRUE, pamStage = FALSE, pamRespectsDendro = FALSE,
#saveTOMs = FALSE, maxBlockSize = 16000, maxPOutliers = 0.05,networkType = "signed hybrid",
#saveTOMFileBase = "femaleMouseTOM", robustY = FALSE, #consensusQuantile = 0.2,
#verbose = 3, pearsonFallback = "individual")

print(names(net))

print(table(net$colors))

# Convert labels to colors for plotting
mergedColors = labels2colors(net$colors)
# Plot the dendrogram and the module colors underneath
plotDendroAndColors(net$dendrograms[[1]], mergedColors[net$blockGenes[[1]]],
"Module colors",
dendroLabels = FALSE, hang = 0.03,
addGuide = TRUE, guideHang = 0.05)

moduleLabels = net$colors
moduleColors = labels2colors(net$colors)
MEs = net$MEs;
geneTree = net$dendrograms[[1]];
save(net, MEs, moduleLabels, moduleColors, mergedColors, geneTree, file = "/content/drive/My Drive/datasets/scz_networkCons

```

```
#_______________________________________________________________________________ Plot one dendrogram for each block
%%R

# Load the results of single-block analysis
load(file = "/content/drive/My Drive/datasets/scz_networkConstruction-auto.RData");
# Relabel blockwise modules
bwLabels = matchLabels(net$colors, moduleLabels);
# Convert labels to colors for plotting
bwModuleColors = labels2colors(bwLabels)

# Plot the dendrogram and the module colors underneath for block 1
plotDendroAndColors(net$dendrograms[[1]], bwModuleColors[net$blockGenes[[1]]],
"Module colors", main = "Gene dendrogram and module colors in block 1",
dendroLabels = FALSE, hang = 0.03,
addGuide = TRUE, guideHang = 0.05)

# Plot the dendrogram and the module colors underneath for block 2
plotDendroAndColors(net$dendrograms[[2]], bwModuleColors[net$blockGenes[[2]]],
"Module colors", main = "Gene dendrogram and module colors in block 2",
dendroLabels = FALSE, hang = 0.03,
addGuide = TRUE, guideHang = 0.05)

# Plot the dendrogram and the module colors underneath for block 3
plotDendroAndColors(net$dendrograms[[3]], bwModuleColors[net$blockGenes[[3]]],
"Module colors", main = "Gene dendrogram and module colors in block 3",
dendroLabels = FALSE, hang = 0.03,
addGuide = TRUE, guideHang = 0.05)
```

```
%%R

if (!requireNamespace("BiocManager", quietly = TRUE))
    install.packages("BiocManager")

```

```
BiocManager::install("biomaRt", force = TRUE)
library(biomaRt)

mart <- useMart("ENSEMBL_MART_ENSEMBL")
mart <- useDataset("hsapiens_gene_ensembl", mart)

devtools::install_github(c("GuangchuangYu/DOSE", "GuangchuangYu/enrichplot", "GuangchuangYu/GOSemSim", "eliocamp/ggnewscale

BiocManager::install("clusterProfiler")
library(clusterProfiler)

BiocManager::install("ReactomePA")
library(ReactomePA)

organism = "org.Hs.eg.db"
BiocManager::install(organism, character.only = TRUE)
library(organism, character.only = TRUE)

```

```
%%R

datTraits <- n13_pca_path[which(keepSamples != 0),]

diagnosis = as.data.frame(datTraits$diag)
names(diagnosis) = "diagnosis"
MEs0 = moduleEigengenes(datExpr, mergedColors)$eigengenes
MEsFemale = orderMEs(MEs0)
modTraitCor = cor(MEsFemale, datTraits, use = "p")
modTraitP = corPvalueStudent(modTraitCor, nSamples)
# Since we have a moderately large number of modules and traits, a
# suitable graphical representation will help in reading the table. We
# color code each association by the correlation value: Will display
# correlations and their p-values
textMatrix = paste(signif(modTraitCor, 2), "\n(", signif(modTraitP, 1), ")",
    sep = "")
dim(textMatrix) = dim(modTraitCor)
par(mar = c(6, 8.5, 3, 3))
# Display the correlation values within a heatmap plot
labeledHeatmap(Matrix = modTraitCor, xLabels = names(datTraits), yLabels = names(MEsFemale),
    ySymbols = names(MEsFemale), colorLabels = FALSE, colors = greenWhiteRed(50),
    #textMatrix = textMatrix,
    setStdMargins = FALSE, cex.text = 0.5, zlim = c(-1,
        1), main = paste("Module-trait relationships"))
                                                                                #The resulting color-coded table is shown ir
                                                                                #the several significant module-trait associ
                                                                                #(first column) as the trait of interest.

                                                                                #Caption: Table of module-trait correlations
                                                                                #correlation (and p-value) resulting from cc
                                                                                #traits (columns). The table is color-coded
                                                                                #legend.

```

```
%%R

#________________________________________________________________________________ Plot Module Significance in a Barplot

GS1=as.numeric(cor(datTraits$diag,datExpr, use="p"))
GeneSignificance=abs(GS1)
# Next module significance is defined as average gene significance.
ModuleSignificance=tapply(GeneSignificance, mergedColors, mean, na.rm=T)

par(mfrow = c(1,1))
plotModuleSignificance(GeneSignificance,mergedColors)
```

```
%%R

library("data.table")

p.sig = signif(cor(datTraits$diag,MEs0, use="p"),2)
#cor.test(as.numeric(datTraits$diag), MEs0$MEbrown)
p.values = corPvalueStudent(cor(datTraits$diag,MEs0, use="p"), nSamples = length(datTraits$diag))


p.values_tmp = as.data.frame(apply(p.values, 2, unlist))
```

```
colnames(p.values_tmp)[1] <- "name2"
p.values_tmp <- setDT(p.values_tmp, keep.rownames = TRUE)[]
colnames(p.values_tmp)[1] <- "name1"

data_mod1 <- p.values_tmp[order(p.values, decreasing = F)]
data_mod1 <- data.table(data_mod1)
data_mod1 <- data_mod1[ , head(.SD, 25)]
print(data_mod1)

print("_______________")

p.sig_tmp = as.data.frame(apply(p.sig, 2, unlist))
colnames(p.sig_tmp)[1] <- "name2"
p.sig_tmp <- setDT(p.sig_tmp, keep.rownames = TRUE)[]
colnames(p.sig_tmp)[1] <- "name1"
head(p.sig_tmp)


data_mod2 <- p.sig_tmp[order(abs(p.sig), decreasing = T)]
data_mod2 <- data.table(data_mod2)
data_mod2 <- data_mod2[ , head(.SD, 25)]
print(data_mod2)
```

```
%%R

#print(table(mergedColors))

#print(which(as.data.frame(table(mergedColors)) == "grey", arr.ind = TRUE))

print(which(as.data.frame(table(mergedColors)) == "mistyrose", arr.ind = TRUE))
print(which(as.data.frame(table(mergedColors)) == "honeydew", arr.ind = TRUE))
print(which(as.data.frame(table(mergedColors)) == "yellow", arr.ind = TRUE))
print(which(as.data.frame(table(mergedColors)) == "white", arr.ind = TRUE))
print(which(as.data.frame(table(mergedColors)) == "blue2", arr.ind = TRUE))
print(which(as.data.frame(table(mergedColors)) == "black", arr.ind = TRUE))
print(which(as.data.frame(table(mergedColors)) == "yellow3", arr.ind = TRUE))
print(which(as.data.frame(table(mergedColors)) == "maroon", arr.ind = TRUE))
print(which(as.data.frame(table(mergedColors)) == "darkturquoise", arr.ind = TRUE))
print(which(as.data.frame(table(mergedColors)) == "darkred", arr.ind = TRUE))
print(which(as.data.frame(table(mergedColors)) == "sienna2", arr.ind = TRUE))
print(which(as.data.frame(table(mergedColors)) == "darkgreen", arr.ind = TRUE))
print(which(as.data.frame(table(mergedColors)) == "turquoise", arr.ind = TRUE))
print(which(as.data.frame(table(mergedColors)) == "lightpink2", arr.ind = TRUE))
print(which(as.data.frame(table(mergedColors)) == "blue4", arr.ind = TRUE))
print(which(as.data.frame(table(mergedColors)) == "lightyellow", arr.ind = TRUE))
print(which(as.data.frame(table(mergedColors)) == "darkgrey", arr.ind = TRUE))
print(which(as.data.frame(table(mergedColors)) == "sienna3", arr.ind = TRUE))
print(which(as.data.frame(table(mergedColors)) == "thistle3", arr.ind = TRUE))
print(which(as.data.frame(table(mergedColors)) == "lightgreen", arr.ind = TRUE))
print(which(as.data.frame(table(mergedColors)) == "pink", arr.ind = TRUE))
print(which(as.data.frame(table(mergedColors)) == "indianred4", arr.ind = TRUE))
print(which(as.data.frame(table(mergedColors)) == "green", arr.ind = TRUE))
print(which(as.data.frame(table(mergedColors)) == "grey60", arr.ind = TRUE))
print(which(as.data.frame(table(mergedColors)) == "honeydew1", arr.ind = TRUE))

```

```
%%R
#____________(uncorrected code from Ch.12__________________________________calculate the module membership values (aka. modu

                                                                        #_________ Caption: Gene significance (GS.weight) ver
                                                                        #_________ for the body weight related modules. GS.we
                                                                        #_________ correlated reflecting the high correlatior
                                                                        #_________ respective module eigengenes. We find that
                                                                        #_________ contain genes that have high positive and
                                                                        #_________ with body weight. In contrast, the grey "t
                                                                        #_________ correlations with weight.
'''
# Next use this trait to define a gene significance variable
GS.diagnosis = as.numeric(cor(datExpr, diagnosis, use = "p"))
# This translates the numeric values into colors
GS.diagnosisColor = numbers2colors(GS.diagnosis, signed = T)
blocknumber = 1
#datColors = data.frame(mergedColors, GS.diagnosisColor)[net$blockGenes[[blocknumber]], ]

datKME = signedKME(datExpr, MEsFemale)

```

```
colorOfColumn = substring(names(datKME), first = 4)
selectModules = c("mistyrose","honeydew","yellow","white","blue2","black","yellow3",
                  "maroon","darkturquoise","darkred","sienna2","darkgreen","turquoise",
                  "lightpink2","blue4","lightyellow","darkgrey","sienna3","thistle3",
                  "lightgreen","pink","indianred4","green","grey60","honeydew1")
for (module in selectModules) {
    column = match(module, colorOfColumn)
    restModule = mergedColors == module
    verboseScatterplot(datKME[restModule, column], GS.diagnosis[restModule], xlab = paste("Module Membership ",
        module, "module"), ylab = "diagnosis", main = paste("kME.", module,
        "vs. diag"), col = module)
}
'''
```

```
%%R
#_________________(corrected code from Ch.12)_____________________________ calculate the module membership values (aka. moc

                                                                              #_________ Caption: Gene significance (GS.weight) ver
                                                                              #_________ for the body weight related modules. GS.we
                                                                              #_________ correlated reflecting the high correlatior
                                                                              #_________ respective module eigengenes. We find that
                                                                              #_________ contain genes that have high positive and
                                                                              #_________ with body weight. In contrast, the grey “b
                                                                              #_________ correlations with weight.

datKME = signedKME(datExpr, MEsFemale)

colorOfColumn = substring(names(datKME), first = 4)
#selectModules = c("lightcyan1","red","paleturquoise","navajowhite2","lightgreen","brown","cyan")
selectModules = c("mistyrose","honeydew","yellow","white","blue2","black","yellow3",
                  "maroon","darkturquoise","darkred","sienna2","darkgreen","turquoise",
                  "lightpink2","blue4","lightyellow","darkgrey","sienna3","thistle3",
                  "lightgreen","pink","indianred4","green","grey60","honeydew1")

for (module in selectModules) {
restModule = mergedColors == module
column = match(module, colorOfColumn)
verboseScatterplot(datKME[restModule, column], GS1[restModule], xlab = paste("Module Membership ",
        module, "module"), ylab = "diagnosis", main = paste("kME.", module,
        "vs. diag"), col = module)
}

#_______________________________
#selectModules = c("lightpink3")
#for (module in selectModules) {
#restModule = mergedColors == module
#print(Alldegrees1$kWithin[restModule])
#meta=na.omit(Alldegrees1$kWithin[restModule])
#}
#length(meta)
```

```
%%R
                                                              #__________________ Our previous analysis has shown that the
                                                              #__________________ “interesting” module in that its module s
                                                              #__________________ Here we show how to find genes with high
                                                              #__________________ high intramodular connectivity in the brc
#print(names(datKME))  #______ check names list


FilterGenes1= abs(GS1)> .2 & abs(datKME$kMEmistyrose)>.8
table(FilterGenes1)
dimnames(data.frame(datExpr))[[2]][FilterGenes1]

FilterGenes2= abs(GS1)> .2 & abs(datKME$kMEhoneydew)>.8
table(FilterGenes2)
dimnames(data.frame(datExpr))[[2]][FilterGenes2]

FilterGenes3= abs(GS1)> .2 & abs(datKME$kMEyellow)>.8
table(FilterGenes3)
dimnames(data.frame(datExpr))[[2]][FilterGenes3]

FilterGenes4= abs(GS1)> .2 & abs(datKME$kMEwhite)>.8
table(FilterGenes4)
dimnames(data.frame(datExpr))[[2]][FilterGenes4]

FilterGenes5= abs(GS1)> .2 & abs(datKME$kMEblue2)>.8
```

```
table(FilterGenes5)
dimnames(data.frame(datExpr))[[2]][FilterGenes5]

FilterGenes6= abs(GS1)> .2 & abs(datKME$kMEblack)>.8
table(FilterGenes6)
dimnames(data.frame(datExpr))[[2]][FilterGenes6]

FilterGenes7= abs(GS1)> .2 & abs(datKME$kMEyellow3)>.8
table(FilterGenes7)
dimnames(data.frame(datExpr))[[2]][FilterGenes7]

FilterGenes8= abs(GS1)> .2 & abs(datKME$kMEmaroon)>.8
table(FilterGenes8)
dimnames(data.frame(datExpr))[[2]][FilterGenes8]

FilterGenes9= abs(GS1)> .2 & abs(datKME$kMEdarkturquoise)>.8
table(FilterGenes9)
dimnames(data.frame(datExpr))[[2]][FilterGenes9]

FilterGenes10= abs(GS1)> .2 & abs(datKME$kMEdarkred)>.8
table(FilterGenes10)
dimnames(data.frame(datExpr))[[2]][FilterGenes10]

FilterGenes11= abs(GS1)> .2 & abs(datKME$kMEsienna2)>.8
table(FilterGenes11)
dimnames(data.frame(datExpr))[[2]][FilterGenes11]

FilterGenes12= abs(GS1)> .2 & abs(datKME$kMEdarkgreen)>.8
table(FilterGenes12)
dimnames(data.frame(datExpr))[[2]][FilterGenes12]

FilterGenes13= abs(GS1)> .2 & abs(datKME$kMEturquoise)>.8
table(FilterGenes13)
dimnames(data.frame(datExpr))[[2]][FilterGenes13]

FilterGenes14= abs(GS1)> .2 & abs(datKME$kMElightpink2)>.8
table(FilterGenes14)
dimnames(data.frame(datExpr))[[2]][FilterGenes14]

FilterGenes15= abs(GS1)> .2 & abs(datKME$kMEblue4)>.8
table(FilterGenes15)
dimnames(data.frame(datExpr))[[2]][FilterGenes15]

FilterGenes16= abs(GS1)> .2 & abs(datKME$kMElightyellow)>.8
table(FilterGenes16)
dimnames(data.frame(datExpr))[[2]][FilterGenes16]

FilterGenes17= abs(GS1)> .2 & abs(datKME$kMEdarkgrey)>.8
table(FilterGenes17)
dimnames(data.frame(datExpr))[[2]][FilterGenes17]

FilterGenes18= abs(GS1)> .2 & abs(datKME$kMEsienna3)>.8
table(FilterGenes18)
dimnames(data.frame(datExpr))[[2]][FilterGenes18]

FilterGenes19= abs(GS1)> .2 & abs(datKME$kMEthistle3)>.8
table(FilterGenes19)
dimnames(data.frame(datExpr))[[2]][FilterGenes19]

FilterGenes20= abs(GS1)> .2 & abs(datKME$kMElightgreen)>.8
table(FilterGenes20)
dimnames(data.frame(datExpr))[[2]][FilterGenes20]

FilterGenes21= abs(GS1)> .2 & abs(datKME$kMEpink)>.8
table(FilterGenes21)
dimnames(data.frame(datExpr))[[2]][FilterGenes21]

FilterGenes22= abs(GS1)> .2 & abs(datKME$kMEindianred4)>.8
table(FilterGenes22)
dimnames(data.frame(datExpr))[[2]][FilterGenes22]

FilterGenes23= abs(GS1)> .2 & abs(datKME$kMEgreen)>.8
table(FilterGenes23)
dimnames(data.frame(datExpr))[[2]][FilterGenes23]

FilterGenes24= abs(GS1)> .2 & abs(datKME$kMEgrey60)>.8
table(FilterGenes24)
```

```
dimnames(data.frame(datExpr))[[2]][FilterGenes24]

FilterGenes25= abs(GS1)> .2 & abs(datKME$kMEhoneydew1)>.8
table(FilterGenes25)
dimnames(data.frame(datExpr))[[2]][FilterGenes25]

```

```
%%R

whichmodule = "mistyrose"                                                         # The heatmap color-codes the scaled gene e
Eigengene = MEsFemale$MEmistyrose                                                       #  red corresponds to over-expression
datExprModule = datExpr[, mergedColors == whichmodule]
# set the margins of the graphics window
par(mfrow = c(1, 1), mar = c(0.3, 5.5, 3, 2))
# create a heatmap whose columns correspond to the arrays and whose rows
# correspond to genes
plotMat(t(scale(datExprModule)), cex.axis = 2, nrgcols = 30, rlabels = F, rcols = whichmodule,
    main = paste("heatmap", whichmodule, "module"))

# scatter plot between eigengene and sample network connectivity               # Scatter plot of the lightgreen module eig
par(mfrow = c(1, 1))                                                          #sample network connectivity Z.k, which is
verboseScatterplot(Eigengene, Z.k, xlab = paste("ME", whichmodule, sep = ""))  #module eigengene takes on an extreme negat
abline(h = -2, col = "red", lwd = 2)                                          #(corresponding to the sample where most mc
                                                                              #The red horizontal line in the scatter plc

whichmodule = "honeydew"
Eigengene = MEsFemale$MEhoneydew
datExprModule = datExpr[, mergedColors == whichmodule]
# set the margins of the graphics window
par(mfrow = c(1, 1), mar = c(0.3, 5.5, 3, 2))
# create a heatmap whose columns correspond to the arrays and whose rows
# correspond to genes
plotMat(t(scale(datExprModule)), cex.axis = 2, nrgcols = 30, rlabels = F, rcols = whichmodule,
    main = paste("heatmap", whichmodule, "module"))
# scatter plot between eigengene and sample network connectivity
par(mfrow = c(1, 1))
verboseScatterplot(Eigengene, Z.k, xlab = paste("ME", whichmodule, sep = ""))
abline(h = -2, col = "red", lwd = 2)

whichmodule = "yellow"
Eigengene = MEsFemale$MEyellow
datExprModule = datExpr[, mergedColors == whichmodule]
# set the margins of the graphics window
par(mfrow = c(1, 1), mar = c(0.3, 5.5, 3, 2))
# create a heatmap whose columns correspond to the arrays and whose rows
# correspond to genes
plotMat(t(scale(datExprModule)), cex.axis = 2, nrgcols = 30, rlabels = F, rcols = whichmodule,
    main = paste("heatmap", whichmodule, "module"))
# scatter plot between eigengene and sample network connectivity
par(mfrow = c(1, 1))
verboseScatterplot(Eigengene, Z.k, xlab = paste("ME", whichmodule, sep = ""))
abline(h = -2, col = "red", lwd = 2)

whichmodule = "white"
Eigengene = MEsFemale$MEwhite
datExprModule = datExpr[, mergedColors == whichmodule]
# set the margins of the graphics window
par(mfrow = c(1, 1), mar = c(0.3, 5.5, 3, 2))
# create a heatmap whose columns correspond to the arrays and whose rows
# correspond to genes
plotMat(t(scale(datExprModule)), cex.axis = 2, nrgcols = 30, rlabels = F, rcols = whichmodule,
    main = paste("heatmap", whichmodule, "module"))
# scatter plot between eigengene and sample network connectivity
par(mfrow = c(1, 1))
verboseScatterplot(Eigengene, Z.k, xlab = paste("ME", whichmodule, sep = ""))
abline(h = -2, col = "red", lwd = 2)

whichmodule = "blue2"
Eigengene = MEsFemale$MEblue2
datExprModule = datExpr[, mergedColors == whichmodule]
# set the margins of the graphics window
par(mfrow = c(1, 1), mar = c(0.3, 5.5, 3, 2))
# create a heatmap whose columns correspond to the arrays and whose rows
# correspond to genes
plotMat(t(scale(datExprModule)), cex.axis = 2, nrgcols = 30, rlabels = F, rcols = whichmodule,
    main = paste("heatmap", whichmodule, "module"))
```

```
# scatter plot between eigengene and sample network connectivity
par(mfrow = c(1, 1))
verboseScatterplot(Eigengene, Z.k, xlab = paste("ME", whichmodule, sep = ""))
abline(h = -2, col = "red", lwd = 2)

#______________________________________________________________________________________________________________________
par(mfrow=c(3,1), mar=c(1, 2, 4, 1))
which.module="mistyrose";
plotMat(t(scale(datExpr[,mergedColors==which.module ]) ),nrgcols=30,rlabels=T,
clabels=T,rcols=which.module,
title=which.module )
# for the second (blue) module we use
which.module="honeydew";
plotMat(t(scale(datExpr[,mergedColors==which.module ]) ),nrgcols=30,rlabels=T,
clabels=T,rcols=which.module,
title=which.module )
which.module="yellow";
plotMat(t(scale(datExpr[,mergedColors==which.module ]) ),nrgcols=30,rlabels=T,
clabels=T,rcols=which.module,
title=which.module )
which.module="white";
plotMat(t(scale(datExpr[,mergedColors==which.module ]) ),nrgcols=30,rlabels=T,
clabels=T,rcols=which.module,
title=which.module )
which.module="blue2";
plotMat(t(scale(datExpr[,mergedColors==which.module ]) ),nrgcols=30,rlabels=T,
clabels=T,rcols=which.module,
title=which.module )

#______________________________________________________________________________________________________________________
which.module="mistyrose"                                                                # Produces heatmap plot for a module.
ME=MEs0[, paste("ME",which.module, sep="")]                                             # Here the rows are genes and the columns a
par(mfrow=c(2,1), mar=c(0.3, 5.5, 3, 2))                                                # Well defined modules results in character
plotMat(t(scale(datExpr[,mergedColors==which.module ]) ),                               # structures since the corresponding genes
nrgcols=30,rlabels=F,rcols=which.module,
main=which.module, cex.main=2)
par(mar=c(5, 4.2, 0, 0.7))
barplot(ME, col=which.module, main="", cex.main=2,
ylab="eigengene expression",xlab="array sample")
#table(sign(ME))

which.module="honeydew"
ME=MEs0[, paste("ME",which.module, sep="")]
par(mfrow=c(2,1), mar=c(0.3, 5.5, 3, 2))
plotMat(t(scale(datExpr[,mergedColors==which.module ]) ),
nrgcols=30,rlabels=F,rcols=which.module,
main=which.module, cex.main=2)
par(mar=c(5, 4.2, 0, 0.7))
barplot(ME, col=which.module, main="", cex.main=2,
ylab="eigengene expression",xlab="array sample")
#table(sign(ME))

which.module="yellow"
ME=MEs0[, paste("ME",which.module, sep="")]
par(mfrow=c(2,1), mar=c(0.3, 5.5, 3, 2))
plotMat(t(scale(datExpr[,mergedColors==which.module ]) ),
nrgcols=30,rlabels=F,rcols=which.module,
main=which.module, cex.main=2)
par(mar=c(5, 4.2, 0, 0.7))
barplot(ME, col=which.module, main="", cex.main=2,
ylab="eigengene expression",xlab="array sample")
#table(sign(ME))

which.module="white"
ME=MEs0[, paste("ME",which.module, sep="")]
par(mfrow=c(2,1), mar=c(0.3, 5.5, 3, 2))
plotMat(t(scale(datExpr[,mergedColors==which.module ]) ),
nrgcols=30,rlabels=F,rcols=which.module,
main=which.module, cex.main=2)
par(mar=c(5, 4.2, 0, 0.7))
barplot(ME, col=which.module, main="", cex.main=2,
ylab="eigengene expression",xlab="array sample")
#table(sign(ME))

which.module="blue2"
ME=MEs0[, paste("ME",which.module, sep="")]
par(mfrow=c(2,1), mar=c(0.3, 5.5, 3, 2))
```

```
plotMat(t(scale(datExpr[,mergedColors==which.module ]) ),
nrgcols=30,rlabels=F,rcols=which.module,
main=which.module, cex.main=2)
par(mar=c(5, 4.2, 0, 0.7))
barplot(ME, col=which.module, main="", cex.main=2,
ylab="eigengene expression",xlab="array sample")
#table(sign(ME))


```

```
%%R
'''
#__________________________________________________________________________________ Correlating tSNE position with eigenvector
install.packages("tsne")
install.packages("Rtsne")
install.packages("ggplot2")
library(tsne)
library(ggplot2)
library("Rtsne")

seed <- 1234
set.seed(seed)

                                                        #_______________________ 1. tsne directly on the original data with
whichmodule = "blue2"
Eigengene = MEsFemale$MEblue2
datExprModule = datExpr[, mergedColors == whichmodule]

dat.filter.DCV.tsne<-tsne(datExprModule,perplexity=30,max_iter=2000,whiten = FALSE)

eigentSNE<-cbind(dat.filter.DCV.tsne[,1],dat.filter.DCV.tsne[,2],Eigengene)
colnames(eigentSNE)<-c("tSNE1_pos","tSNE2_pos",whichmodule)

p2.subset<-ggplot(data.frame(eigentSNE)) + geom_point(aes(x=tSNE1_pos,y=tSNE2_pos,color = blue2),size=4) + scale_color_grac
p2.subset.all <- p2.subset + ggtitle("WGCNA module eigengene expression") + coord_fixed(ratio = 1) + xlab("t-SNE 1") + ylab
p2.subset.all

                                                        #_______________________ 2. tsne on data after PCA

#whichmodule = "blue2"
#datExprModule = datExpr[, mergedColors == whichmodule]

#p <- pca(t(datExprModule), metadata = datTraits, center = TRUE,
#         scale = TRUE)
#elbow <- findElbowPoint(p$variance)
#screeplot(p,components = getComponents(p, 1:20),vline = c(elbow)) +
#    geom_label(aes(x = elbow + 1, y = 50,
#      label = 'Elbow method', vjust = -1, size = 8))

#dat.filter.DCV.tsne<-Rtsne(datExprModule,perplexity=30,max_iter=2000,whiten = FALSE,
#                           PCA = True, initial_dims = elbow + 1)
#plot(dat.filter.DCV.tsne$Y,col="blue",xlab="tSNE1",ylab="tSNE2",cex=0.5)
'''

```

```
%%R

BiocManager::install(c("dynamicTreeCut", "cluster", "flashClust", "Hmisc", "reshape", "foreach", "doParallel") )
library(dynamicTreeCut)
library(flashClust)
library(Hmisc)
library(reshape)
library(foreach)
library(doParallel)
```

```
%%R
                                                                #______________ Remove overrepresented modules (42->grey,1
# Calculate eigengenes for each module
MEs <- moduleEigengenes(datExpr, colors = mergedColors)$eigengenes

#print(names(MEs[120]))  #______________ how to remove genes
#print(names(MEs[42]))
#MEs <- MEs[,c(1:41,43:119,121:length(table(mergedColors)))]

print(names(MEs[78]))    #______________ how to add genes
```

```
print(names(MEs[44]))
print(names(MEs[127]))
print(names(MEs[126]))
print(names(MEs[7]))
print(names(MEs[5]))
print(names(MEs[129]))
print(names(MEs[70]))
print(names(MEs[33]))
print(names(MEs[28]))
print(names(MEs[107]))
print(names(MEs[20]))
print(names(MEs[124]))
print(names(MEs[59]))
print(names(MEs[8]))
print(names(MEs[66]))
print(names(MEs[21]))
print(names(MEs[108]))
print(names(MEs[122]))
print(names(MEs[58]))
print(names(MEs[91]))
print(names(MEs[48]))
print(names(MEs[39]))
print(names(MEs[43]))
print(names(MEs[45]))

MEs <- MEs[,c(78,44,127,126,7,5,129,70,33,28,107,20,124,59,8,66,21,108,122,58,91,48,39,43,45)]

```

```
%%R

install.packages("gplots")
library(gplots)
#myheatcol = colorpanel(250,'red',"orange",'lemonchiffon')

#________________________________________________________________ Remove grey and turquoise for ahead of attempting to look
modNames <- substring(names(MEs), 3)
#restGenes= (mergedColors != "grey" & mergedColors != "turquoise")
restGenes = (mergedColors != "mistyrose" & mergedColors != "honeydew" & mergedColors != "yellow" & mergedColors != "white"

table(!restGenes)

softPower = 12
diss1=1-TOMsimilarityFromExpr(datExpr[,!restGenes], power = softPower, TOMType = "signed")

colnames(diss1) =rownames(diss1) = modNames[!restGenes]
hier1=flashClust(as.dist(diss1), method="average" )
plotDendroAndColors(hier1, mergedColors[!restGenes], "Dynamic Tree Cut", dendroLabels = FALSE,
                    hang = 0.03, addGuide = TRUE, guideHang = 0.05, main = "Gene dendrogram and module colors")

#____________________________ The commented code below uses a lot of RAM
                                                                                # The TOM plot provides a reduced view of th
                                                                                # one to visualize and identify network modu
                                                                                # red indicates high adjacency (positive cor
                                                                                # low adjacency (negative correlation)
#diag(diss1) = NA;
#TOMplot(diss1^7, hier1, as.character(mergedColors[!restGenes]),
#main = "TOM heatmap plot, module genes")        # or add: "module genes" , col=myheatcol"


#diss1 = 1-adjacency( datExpr[, !restGenes], power = softPower, type='signed' )
#hier1 = hclust(as.dist(diss1), method="average" )
#diag(diss1) = NA;
#TOMplot(diss1^7, hier1, as.character(mergedColors[!restGenes]),
#main = "Adjacency heatmap plot, module genes")  # or add: "module genes" , col=myheatcol"

```

```
%%R

install.packages("reshape2")
library(ggplot2)
library(reshape2)

if (!requireNamespace('ComplexHeatmap', quietly = TRUE))
    BiocManager::install('ComplexHeatmap')
require(ComplexHeatmap)

```

```
if (!requireNamespace('circlize', quietly = TRUE))
    BiocManager::install('circlize')
require(circlize)

df <- data.frame(a = mergedColors[!restGenes], c = c(1:length(mergedColors[!restGenes])))

df.newer <- df %>%
             group_by(a) %>%
             summarise(new_strs = c(c))

heat = datExpr[,df.newer$new_strs]
heat <- scale(heat)
column_order <- MEs

l = list()
k = 0
l[1] = 0

for(i in 1:length(selectModules)+1){
k = k + table(df.newer$a)[[i-1]]
l[i] <- k
}

#print(l[120])
#print(length(mergedColors[restGenes]))
#print("________________")

#for(i in 1:119){
#print(dim(heat[,(l[[i]]+1):l[[i+1]]]))
#}
#print("________________________________________________________________________")
#print(table(mergedColors[restGenes]))
#print("________________________________________________________________________")
#print(table(df.newer$a))
#print("________________________________________________________________________")
#print(dim(heat[,(l[[1]]+1):l[[1+1]]]))
#print(length(column_order[,1]))
#print(dim(cor(heat[,(l[[1]]+1):l[[1+1]]], column_order[,1])))
#print("_________________________________________________________________________")
#for (i in 1:length(colnames(COR3))){
#print(dim(datExpr[, mergedColors == substring(colnames(COR3)[[i]], first = 3)]))
#}
#print("_________________________________________________________________________")
#for (i in 1:119){
#  print(dim(heat[,(l[[i]]+1):l[[i+1]]]))
#}

COR3 <- array(numeric(),c(length(mergedColors[!restGenes]),length(selectModules)))
COR4 <- array(numeric(),c(length(selectModules),length(selectModules)))

for(i in 1:length(selectModules)){
 for (j in 1:length(selectModules)){
  COR3[(l[[i]]+1):l[[i+1]],j] <- cor(heat[,(l[[i]]+1):l[[i+1]]], column_order[,j], method = "spearman")
  COR4[i,j] <- mean(COR3[(l[[i]]+1):l[[i+1]],j])
   }
 }
dim(COR3)

```

```
%%R

#________________________________________________________________________________ Correlating tSNE position with eigenvector

install.packages("tsne")
install.packages("ggplot2")
library(tsne)
library(ggplot2)

seed <- 1234
set.seed(seed)

Eigengene = MEsFemale
dat.filter.DCV.tsne<-tsne(heat,perplexity=30,max_iter=2000,whiten = FALSE)

eigentSNE<-cbind(dat.filter.DCV.tsne[,1],dat.filter.DCV.tsne[,2],MEs)
colnames(eigentSNE)<-c("tSNE1_pos","tSNE2_pos",modNames)
```

```
eigentSNE.melt<-melt(eigentSNE,id.vars=c("tSNE1_pos","tSNE2_pos"))

# Melting the data and selecting only the modules we want to plot
eigentSNE.melt.subset <- eigentSNE.melt %>%
  filter(variable == "mistyrose" | variable == "honeydew" | variable == "yellow" | variable == "white" | variable ==
           "blue2" | variable == "black" | variable == "yellow3" | variable == "maroon" | variable ==
           "darkturquoise" | variable == "darkred" | variable == "sienna2" | variable ==
           "darkgreen" | variable == "turquoise")

p2.subset<-ggplot(eigentSNE.melt.subset) + geom_point(aes(x=tSNE1_pos,y=tSNE2_pos,color=value),size=1) + scale_color_gradie

p2.subset.all <- p2.subset + facet_wrap("variable", nrow = 2) + ggtitle("WGCNA module eigengene") + coord_fixed(ratio = 1)

p2.subset.all
```

```
%%R                                                   #________________________ Correlation VIIc.

#incq <- which(names(datExpr[1,!restGenes][df.newer$new_strs]) %in% names(datExpr[1, !restGenes]))
#print(length(incq))

#hgt <- scale(datExpr[,!restGenes])
hgt <- datExpr[,!restGenes]
hgt_col <- mergedColors[!restGenes]

MEsFemale = orderMEs(moduleEigengenes(hgt[,df.newer$new_strs], hgt_col[df.newer$new_strs])$eigengenes)
datKME1 = signedKME(hgt[,df.newer$new_strs], MEsFemale, outputColumnName="MM.")

hmap <- ComplexHeatmap::Heatmap(datKME1, name = "CORRELATION", row_order = rownames(datKME1),
    column_order = colnames(datKME1),
    column_names_gp = grid::gpar(fontsize = 8),
    column_title = "modules")

genelabels <- rowAnnotation(
    Genes = anno_mark(
      at = seq(1, nrow(datKME1), 300),
      labels = rownames(datKME1)[seq(1, nrow(datKME1), 300)],
      labels_gp = gpar(fontsize = 10, fontface = 'bold'),
      padding = 0.75),
      width = unit(2.0, 'cm') +
max_text_width(
        rownames(datKME1)[seq(1, nrow(datKME1), 300)],
        gp = gpar(fontsize = 7,  fontface = 'bold')))

draw(hmap + genelabels)
```

```
'''
%%R                                                   #________________________ Correlation VIIIc.

install.packages("reshape2")
library(ggplot2)
library(reshape2)

if (!requireNamespace('ComplexHeatmap', quietly = TRUE))
    BiocManager::install('ComplexHeatmap')
require(ComplexHeatmap)

if (!requireNamespace('circlize', quietly = TRUE))
    BiocManager::install('circlize')
require(circlize)

sp <- c("-")
dspf <- data.frame(tmp = rep(sp, each = 119))             # reduce it by the number of modules you have excluded from analy
dspf

dspf$tmp[99] <- c("orange")
#dspf$tmp[100] <- c("purple")
dspf$tmp[101] <- c("darkturquoise")
#dspf$tmp[102] <- c("darkolivegreen")
dspf$tmp[103] <- c("coral4")
#dspf$tmp[104] <- c("darkmagenta")
dspf$tmp[105] <- c("pink")
#dspf$tmp[106] <- c("brown")
dspf$tmp[107] <- c("turquoise")
#dspf$tmp[108] <- c("antiquewhite1")
dspf$tmp[109] <- c("red")
```

```
#dspf$tmp[110] <- c("magenta3")
dspf$tmp[111] <- c("thistle1")
#dspf$tmp[112] <- c("deeppink")
dspf$tmp[113] <- c("tan4")
#dspf$tmp[114] <- c("green4")
dspf$tmp[115] <- c("blue4")
#dspf$tmp[116] <- c("blue")
dspf$tmp[117] <- c("skyblue2")
#dspf$tmp[118] <- c("kMEgrey")
dspf$tmp[119] <- c("yellow")
#dspf$tmp[120] <- c("yellow3")
#dspf$tmp[121] <- c("grey")

#incq <- which(names(datExpr[1,restGenes][df.newer$new_strs]) %in% names(datExpr[1, restGenes]))
#print(length(incq))

hgt <- datExpr[,restGenes]
hgt_col <- mergedColors[restGenes]

MEsFemale = orderMEs(moduleEigengenes(hgt[,df.newer$new_strs], hgt_col[df.newer$new_strs])$eigengenes)
datKME1 = signedKME(hgt[,df.newer$new_strs], MEsFemale, outputColumnName="MM.")
colnames(datKME1)<-dspf$tmp
hmap <- ComplexHeatmap::Heatmap(datKME1, name = "CORRELATION", row_order = rownames(datKME1),
    column_order = colnames(datKME1),
    column_title = "modules")

genelabels <- rowAnnotation(
    Genes = anno_mark(
      at = seq(1, nrow(datKME1), 1000),
      labels = rownames(datKME1)[seq(1, nrow(datKME1), 1000)],
      labels_gp = gpar(fontsize = 10, fontface = 'bold'),
      padding = 0.75),
      width = unit(2.0, 'cm') +
max_text_width(
        rownames(datKME1)[seq(1, nrow(datKME1), 1000)],
        gp = gpar(fontsize = 7,  fontface = 'bold')))

draw(hmap + genelabels)
'''
```

```
#_#_#_#_#_#_#_#_#_#_#_#_#_#_#_#_#_#_#_#_#_#_#_#_#_#_#_#_#_#_#_#_#_#_#_#_#_#_#_#_#_#_#_#_#_#_#_#_#_#_#_#_#_#_#_#_#_#_#_#

#_______________________________________________________________ANALYSIS PART B._____________________________________________

#_#_#_#_#_#_#_#_#_#_#_#_#_#_#_#_#_#_#_#_#_#_#_#_#_#_#_#_#_#_#_#_#_#_#_#_#_#_#_#_#_#_#_#_#_#_#_#_#_#_#_#_#_#_#_#_#_#_#_#
```

```
%%R                                       #________________________________________ MDS plot of modules

                                                                 #_____________ Multidimensional scaling (MDS) is an appro
                                                                 #_____________ relationships specified by a dissimilarity
                                                                 #_____________ dissimilarity matrix is visualized by a po

                                                                 #_____________ The Euclidean distances between a pair of
                                                                 #_____________ corresponding pairwise dissimilarity. An M
                                                                 #_____________ dissimilarity matrix as input and assigns
                                                                 #_____________ in d-dimensional space, where d is specifi
                                                                 #_____________ point locations can be displayed in the Eu

                                                                 #_____________ There are two major types of MDS: classica
                                                                 #_____________ function cmdscale) and non-metric MDS (R f
                                                                 #_____________ The following R code shows how to create a
                                                                 #_____________ d=2 scaling dimensions.

#restGenes_new= (mergedColors != "grey" & mergedColors != "turquoise")

adjac1 = adjacency(datExpr[, !restGenes], power = softPower, type='signed');
dissTOM = TOMdist(adjac1)
cmd1 = cmdscale(as.dist(dissTOM), 2)
par(mfrow = c(1, 1))
plot(cmd1, col = mergedColors[!restGenes], main = "MDS plot", xlab = "Dim 1",
    ylab = "Dim2")

```

```
%%R

```

```
datExpr_tmp = datExpr[, !restGenes]
module.order <- unlist(tapply(1:ncol(datExpr_tmp),as.factor(mergedColors[!restGenes]),I))
m<-t(t(datExpr_tmp[,module.order])/apply(datExpr_tmp[,module.order],2,max))
heatmap(t(m),zlim=c(0,1),col=gray.colors(100),Rowv=NA,Colv=NA,labRow=NA,scale="none",RowSideColors=mergedColors[module.orde

```

```
%%R

# Making a list of module names
modNames <- substring(names(MEs), first = 3)

# Correlating each genes expression profile with the module eigengenes in order to create module gene sets
geneModuleMembership <- as.data.frame(cor(datExpr, MEs, use = "p"))
# "For each module, we also define a quantitative measure of module membership MM as the correlation of the
#module eigengene and the gene expression profile."

# Iteratively creating a list of module genesets to test. These are in ensembl ids
moduleGeneSets<-lapply(modNames,function(module){
  column = match(module, modNames)
  moduleGenes = moduleColors==module
  rownames(geneModuleMembership[moduleGenes,])
})

names(moduleGeneSets)<-modNames

# Trimming the module gene sets so that the final two digits after the "." are removed
moduleGeneSets.trimmed<-lapply(moduleGeneSets,function(x){
  str_split_fixed(x,"\\.",2)[,1]
})

# Looking up the ENTREZ id for each gene
moduleGeneSets.Entrez_cmp<-lapply(moduleGeneSets.trimmed,function(x){
  bitr(x,fromType="SYMBOL",toType="ENTREZID",OrgDb="org.Hs.eg.db")$ENTREZID
})

```

```
%%R

moduleGeneSets_tmp = list()
moduleGeneSets.Entrez = list()
moduleGeneSets_entrez_tmp = list()
moduleGeneSets_ensembl_tmp = list()

#__________________________________ I. Use all the genes of the most important modules
#moduleGeneSets_tmp <- list(moduleGeneSets$antiquewhite1,moduleGeneSets$red,moduleGeneSets$coral4,
#moduleGeneSets$lightpink3,moduleGeneSets$brown,moduleGeneSets$skyblue2)

#__________________________________ II. Use only the most important genes of the most important modules
moduleGeneSets_tmp <- list(dimnames(data.frame(datExpr))[[2]][FilterGenes1],dimnames(data.frame(datExpr))[[2]][FilterGenes
dimnames(data.frame(datExpr))[[2]][FilterGenes4],dimnames(data.frame(datExpr))[[2]][FilterGenes5],dimnames(data.frame(datE
dimnames(data.frame(datExpr))[[2]][FilterGenes7],dimnames(data.frame(datExpr))[[2]][FilterGenes8],dimnames(data.frame(datE
dimnames(data.frame(datExpr))[[2]][FilterGenes10],dimnames(data.frame(datExpr))[[2]][FilterGenes11],dimnames(data.frame(da
dimnames(data.frame(datExpr))[[2]][FilterGenes13],dimnames(data.frame(datExpr))[[2]][FilterGenes14],dimnames(data.frame(da
dimnames(data.frame(datExpr))[[2]][FilterGenes16],dimnames(data.frame(datExpr))[[2]][FilterGenes17],dimnames(data.frame(da
dimnames(data.frame(datExpr))[[2]][FilterGenes19],dimnames(data.frame(datExpr))[[2]][FilterGenes20],dimnames(data.frame(da
dimnames(data.frame(datExpr))[[2]][FilterGenes22],dimnames(data.frame(datExpr))[[2]][FilterGenes23],dimnames(data.frame(da
dimnames(data.frame(datExpr))[[2]][FilterGenes25])

df = as.data.frame(org.Hs.egGO)
go_gene_list = unique(sort(df$gene_id))
go_gene_list.df <- bitr(go_gene_list, fromType = "ENTREZID",
        toType = c("ENSEMBL", "SYMBOL"),
        OrgDb = org.Hs.eg.db)

dfk = as.data.frame(org.Hs.egPATH)
kegg_gene_list = unique(sort(dfk$gene_id))


#__________________________________ I. Use all the genes of the most important modules
#moduleGeneSets_entrez_tmp <- list(moduleGeneSets.Entrez$antiquewhite1,moduleGeneSets.Entrez$red,moduleGeneSets.Entrez$cor
#moduleGeneSets.Entrez$lightpink3,moduleGeneSets.Entrez$brown,moduleGeneSets.Entrez$skyblue2)

#__________________________________ II. Use only the most important genes of the most important modules
for(i in 1:length(moduleGeneSets_tmp)) {
    if (length(moduleGeneSets_tmp[[i]])>2){
    print(i)
```

```
    moduleGeneSets.Entrez[[i]] <- bitr(moduleGeneSets_tmp[[i]], fromType = "SYMBOL",
        toType =  c("ENSEMBL", "ENTREZID"),
        OrgDb = org.Hs.eg.db)
    }
}

for(i in 1:length(moduleGeneSets.Entrez)) {
moduleGeneSets_entrez_tmp[[i]] <- list(moduleGeneSets.Entrez[[i]]$ENTREZID)
}

for(i in 1:length(moduleGeneSets.Entrez)) {
moduleGeneSets_ensembl_tmp[[i]] <- list(moduleGeneSets.Entrez[[i]]$ENSEMBL)
}

ego1 = list()
kk = list()

for(i in 1:length(moduleGeneSets_tmp)) {

print("___________________________Ia. enrichGO (BP) ________________________________________________")
l1 <- enrichGO(gene         = unique(sort(unlist(moduleGeneSets_ensembl_tmp[[i]]))),
                 universe      = go_gene_list.df$ENSEMBL,                       #org.Hs.egGO
                 #universe      = geneList.df$ENSEMBL,                         #background genes
                 OrgDb         = org.Hs.eg.db,
                #keyType       = "ENTREZID",
                 keyType       = "ENSEMBL",
                 ont           = "BP",
                 pAdjustMethod = "BH",                                          #pAdjustMethod = "fdr"
                 pvalueCutoff  = 0.05,
                 qvalueCutoff  = 0.1,
                 readable      = TRUE)
if (length(l1$ID) == 0) {
ego1[[i]] <- "NULL"
}
else{
ego1[[i]] <- data.frame(
 i,
 l1$ID,
 l1$Description,
 l1$GeneRatio,
 l1$BgRatio,
 l1$pvalue,
 l1$p.adjust,
 l1$qvalue,
 l1$geneID,
 l1$Count
)
colnames(ego1[[i]]) <- c("Community#", "ID", "Description", "GeneRatio",
                           "BgRatio", "pvalue", "p.adjust", "qvalue",
                           "geneID","Count")
options(scipen=999)
}

print("___________________________III. enrichKEGG_______________________________________________")
l5 <- enrichKEGG(gene         = as.character(unique(sort(unlist(moduleGeneSets_entrez_tmp[[i]])))),
                 organism     = 'hsa',
                 pvalueCutoff = 0.05,
                 universe  = kegg_gene_list,                                    #org.Hs.egPATH
                 #universe = geneList.df$ENTREZID,                              #background genes
                 qvalueCutoff  = 0.1)
if (length(l5$ID) == 0) {
kk[[i]] <- "NULL"
}
else{
kk[[i]] <- data.frame(
 i,
 l5$ID,
 l5$Description,
 l5$GeneRatio,
 l5$BgRatio,
 l5$pvalue,
 l5$p.adjust,
 l5$qvalue,
 l5$geneID,
 l5$Count
)
colnames(kk[[i]]) <- c("Community#", "ID", "Description", "GeneRatio",
```

```
                            "BgRatio", "pvalue", "p.adjust", "qvalue",
                            "geneID","Count")
options(scipen=999)
}
}

```

```
%R -o ego1,kk

for i in range(len(ego1)):
  if (len(ego1[i])!=1):
    df1 = pd.DataFrame(ego1[i]).T
    df1.rename(columns={0: 'Community#', 1: 'ID',
          2: 'Description', 3: 'GeneRatio',
          4: 'BgRatio', 5: 'pvalue',
          6: 'p.adjust', 7: 'qvalue',
          8: 'geneID', 9: 'Count'},inplace=True)
    print(df1.to_string())
    print("##########################################################################################################

print("_____________________________________________________________________________________________________________________
print("_____________________________________________________________________________________________________________________
print("_____________________________________________________________________________________________________________________

for i in range(len(kk)):
  if (len(kk[i])!=1):
    df5 = pd.DataFrame(kk[i]).T
    df5.rename(columns={0: 'Community#', 1: 'ID',
          2: 'Description', 3: 'GeneRatio',
          4: 'BgRatio', 5: 'pvalue',
          6: 'p.adjust', 7: 'qvalue',
          8: 'geneID', 9: 'Count'},inplace=True)
    print(df5.to_string())
    print("##########################################################################################################

```

```
%%R

ck<-compareCluster(geneCluster=moduleGeneSets.Entrez_cmp,
                   fun="enrichKEGG",organism="hsa",pvalueCutoff = 0.05,qvalueCutoff  = 0.1)


cg.mf<-compareCluster(geneCluster=moduleGeneSets,fun="enrichGO",universe  = go_gene_list.df$SYMBOL,keyType = "SYMBOL", pAdj
                      OrgDb = "org.Hs.eg.db",pvalueCutoff = 0.05,qvalueCutoff  = 0.1, ont="MF", readable = F)


cg.bp<-compareCluster(geneCluster=moduleGeneSets,fun="enrichGO",universe  = go_gene_list.df$SYMBOL,keyType = "SYMBOL", pAdj
                      OrgDb = "org.Hs.eg.db", pvalueCutoff = 0.05,qvalueCutoff  = 0.1, ont="BP", readable = F)


cg.cc<-compareCluster(geneCluster=moduleGeneSets,fun="enrichGO",universe  = go_gene_list.df$SYMBOL,keyType = "SYMBOL", pAdj
                      OrgDb = "org.Hs.eg.db", pvalueCutoff = 0.05,qvalueCutoff  = 0.1, ont="CC", readable = F)


cr<-compareCluster(geneCluster=moduleGeneSets.Entrez_cmp,fun="enrichPathway",
                   organism="human",pvalueCutoff = 0.05, qvalueCutoff  = 0.1, readable = F)


#file_dir = "/content/drive/My Drive/datasets/"
#pdf(file = file.path(file_dir,"en-tts-subset.pdf"), width = 18, height = 30, useDingbats = F)

#selected_pathways <- sample(cg.bp@compareClusterResult$Description, 10)
#selected_pathways <- cg.bp@compareClusterResult$Description[1:10]

selected_pathways_ck <- head(ck@compareClusterResult[order(ck@compareClusterResult$p.adjust, decreasing = F),],10)
selected_pathways_cg.mf <- head(cg.mf@compareClusterResult[order(cg.mf@compareClusterResult$p.adjust, decreasing = F),],10)
selected_pathways_cg.bp <- head(cg.bp@compareClusterResult[order(cg.bp@compareClusterResult$p.adjust, decreasing = F),],10)
selected_pathways_cg.cc <- head(cg.cc@compareClusterResult[order(cg.cc@compareClusterResult$p.adjust, decreasing = F),],10)
selected_pathways_cr <- head(cr@compareClusterResult[order(cr@compareClusterResult$p.adjust, decreasing = F),],10)

blue.bold.italic.16x.text <- element_text(face = "bold.italic", color = "blue", size = 10)
blue.bold.italic.16y.text <- element_text(face = "bold.italic", color = "blue", size = 10)

```

```
%%R


dotplot(ck, showCategory=selected_pathways_ck$Description, font.size=14)+ggtitle("KEGG enrichment")  +
  theme_bw(base_size = 24) + theme(axis.text.x = blue.bold.italic.16x.text, axis.text.y = blue.bold.italic.16y.text) +
scale_x_discrete(guide = guide_axis(angle = 90))

```

```
%%R

dotplot(cg.mf,showCategory=selected_pathways_cg.mf$Description, font.size=14)+ggtitle("GO MF enrichment")  +
  theme_bw(base_size = 24) + theme(axis.text.x = blue.bold.italic.16x.text, axis.text.y = blue.bold.italic.16y.text) +
scale_x_discrete(guide = guide_axis(angle = 90))
```

```
%%R

dotplot(cg.bp,showCategory=selected_pathways_cg.bp$Description, font.size=14)+ggtitle("GO BP enrichment")  +
  theme_bw(base_size = 24) + theme(axis.text.x = blue.bold.italic.16x.text, axis.text.y = blue.bold.italic.16y.text) +
scale_x_discrete(guide = guide_axis(angle = 90))
```

```
%%R

dotplot(cg.cc,showCategory=selected_pathways_cg.cc$Description, font.size=14)+ggtitle("GO CC enrichment")  +
  theme_bw(base_size = 24) + theme(axis.text.x = blue.bold.italic.16x.text, axis.text.y = blue.bold.italic.16y.text) +
scale_x_discrete(guide = guide_axis(angle = 90))
```

```
%%R

dotplot(cr,showCategory=selected_pathways_cr$Description, font.size=14)+ggtitle("Reactome enrichment")  +
  theme_bw(base_size = 24) + theme(axis.text.x = blue.bold.italic.16x.text, axis.text.y = blue.bold.italic.16y.text) +
scale_x_discrete(guide = guide_axis(angle = 90))
```

```
%%R                       #____________________________________________________ Create dataframe with the top genes of the
                                                                               # Use it as input to Reactome enrichment ana

ph_all = list()
pl_all = list()

p1h<-order(p$loadings[1], decreasing=T)[1:5]
p1l<-order(p$loadings[1], decreasing=F)[1:5]

p2h<-order(p$loadings[2], decreasing=T)[1:5]
p2l<-order(p$loadings[2], decreasing=F)[1:5]

p3h<-order(p$loadings[3], decreasing=T)[1:5]
p3l<-order(p$loadings[3], decreasing=F)[1:5]

p4h<-order(p$loadings[4], decreasing=T)[1:5]
p4l<-order(p$loadings[4], decreasing=F)[1:5]

p5h<-order(p$loadings[5], decreasing=T)[1:5]
p5l<-order(p$loadings[5], decreasing=F)[1:5]

ph_all[[1]] <- rownames(p$loadings[p1h,])
ph_all[[2]] <- rownames(p$loadings[p2h,])
ph_all[[3]] <- rownames(p$loadings[p3h,])
ph_all[[4]] <- rownames(p$loadings[p4h,])
ph_all[[5]] <- rownames(p$loadings[p5h,])

pl_all[[1]] <- rownames(p$loadings[p1l,])
pl_all[[2]] <- rownames(p$loadings[p2l,])
pl_all[[3]] <- rownames(p$loadings[p3l,])
pl_all[[4]] <- rownames(p$loadings[p4l,])
pl_all[[5]] <- rownames(p$loadings[p5l,])
```

```
%%R                #___________________________________________________________ Reactome pathway gene set enrichment analy

ll = list()
PCAGeneSets_tmp = list()
                                                                              #________________________ 1.
PCAGeneSets_tmp <- list(ph_all[[1]],ph_all[[2]],ph_all[[3]],ph_all[[4]],ph_all[[5]],
                           pl_all[[1]],pl_all[[2]],pl_all[[3]],pl_all[[4]],pl_all[[5]])

```

```
#pe1 = list()                                                                      #________________________ 2.
#pe2 = list()
#pe3 = list()
#pe4 = list()
#pe5 = list()
#pe1[[1]]<-ph_all[[1]]
#pe1[[2]]<-pl_all[[1]]
#pe2[[1]]<-ph_all[[2]]
#pe2[[2]]<-pl_all[[2]]
#pe3[[1]]<-ph_all[[3]]
#pe3[[2]]<-pl_all[[3]]
#pe4[[1]]<-ph_all[[4]]
#pe4[[2]]<-pl_all[[4]]
#pe5[[1]]<-ph_all[[5]]
#pe5[[2]]<-pl_all[[5]]
#PCAGeneSets_tmp <- list(unlist(pe1),unlist(pe2),unlist(pe3),unlist(pe4),unlist(pe5))

PCAGeneSets_entrez_tmp = list()
PCAGeneSets_entrez_tmp<-lapply(PCAGeneSets_tmp,function(x){
  bitr(x,fromType="SYMBOL",toType="ENTREZID",OrgDb="org.Hs.eg.db")$ENTREZID})

for (i in 1:length(PCAGeneSets_entrez_tmp)){
print("___________________________IV. enrichPathway_________________________________")
l6 <- enrichPathway(gene = as.character(unique(sort(PCAGeneSets_entrez_tmp[[i]]))),
                 pvalueCutoff = 0.05,
                 qvalueCutoff  = 0.1,
                 readable = TRUE)
if (length(l6$ID) == 0) {
ll[[i]] <- "NULL"
}
else{
ll[[i]] <- data.frame(
 i,
 l6$ID,
 l6$Description,
 l6$GeneRatio,
 l6$BgRatio,
 l6$pvalue,
 l6$p.adjust,
 l6$qvalue,
 l6$geneID,
 l6$Count
)
colnames(ll[[i]]) <- c("Community#", "ID", "Description", "GeneRatio",
                            "BgRatio", "pvalue", "p.adjust", "qvalue",
                            "geneID","Count")
options(scipen=999)
print(ll[[i]])
print("##################################################################################################################
}
}

```

```
%%R

# The first step to find these correlations is to make a trait table
# Copying over the datTraits dataframe

file_dir = "/content/drive/My Drive/datasets/"

datTraits.new <- datTraits

datTraits.new$diag <- as.factor(datTraits.new$diag)

for (l in levels(datTraits.new$diag)){
  datTraits.new[[l]] <- datTraits.new$diag == l
}

# Factoring the T/F columns in to a binary 0/1 classification system
datTraitsFactor<-datTraits.new
datTraitsFactor$diag<-as.numeric(as.factor(datTraits.new$diag))

for (l in levels(datTraits.new$diag)){
  datTraitsFactor[[l]]<-as.numeric(datTraits.new[[l]])
}

```

```
# Correlating the module eigengenes with subset cluster identity
moduleTraitCor <- cor(MEs, datTraitsFactor[, -c(6:9)])

# Getting a p-value for each correlation
moduleTraitPvalue <- corPvalueStudent(moduleTraitCor, nSamples)

# Plotting the relationship between learned gene co-expression modules and parameterizations
pdf(file = file.path(file_dir,"module-trait_relationships.pdf"), width = 20, height = 16)

par(mfrow=c(1,1))
textMatrix = paste(signif(moduleTraitCor, 2), "\n(",
signif(moduleTraitPvalue, 1), ")", sep = "")
dim(textMatrix) = dim(moduleTraitCor)
par(mar = c(6, 8.5, 3, 3))

labeledHeatmap(Matrix = t(moduleTraitCor),
               xLabels = names(MEs), yLabels = names(datTraitsFactor[, -c(6:9)]),
               ySymbols = names(datTraitsFactor[, -c(6:9)]),
               colorLabels = FALSE,
               colors = blueWhiteRed(50),
               textMatrix = t(textMatrix),
               setStdMargins = T, cex.text = 0.5, zlim = c(-1,1),
               main = paste("Module-trait relationships"))
dev.off()

```

```
%%R

par(mfrow=c(1,1))
textMatrix = paste(signif(moduleTraitCor, 2), "\n(",
signif(moduleTraitPvalue, 1), ")", sep = "")
dim(textMatrix) = dim(moduleTraitCor)
par(mar = c(6, 8.5, 3, 3))

labeledHeatmap(Matrix = t(moduleTraitCor),
               xLabels = names(MEs), yLabels = names(datTraitsFactor[, -c(6:9)]),
               ySymbols = names(datTraitsFactor[, -c(6:9)]),
               colorLabels = FALSE,
               colors = blueWhiteRed(50),
               #textMatrix = t(textMatrix),
               setStdMargins = T, cex.text = 0.5, zlim = c(-1,1),
               main = paste("Module-trait relationships"))
```

```
%%R

saveRDS(moduleGeneSets$green, file = file.path(file_dir,"WGCNA.ParkGeneSet.Green.rds"))
saveRDS(moduleGeneSets$brown, file = file.path(file_dir,"WGCNA.ParkGeneSet.Brown.rds"))

```
