# Supplementary material for: Exploration of schizophrenia-associated gene modules using graph theory, co-expression networks, and dimensionality reduction
Source: PLoS One. 2026 Apr 15;21(4):e0346663. doi: 10.1371/journal.pone.0346663 (PMC13082716; doi:10.1371/journal.pone.0346663)
Supplement: S1 Code — This archive contains four distinct Jupyter Notebooks used for the analysis: SVA_Diagnostics_and_PCA_Loadings.ipynb, PCA_Variance_Driven_Reactome_Enrichment.ipynb, WGCNA_Module_Functional_Enrichment.ipynb, and igraph_MST_Topological_Centrality.ipynb. (ZIP) [file pone.0346663.s008.zip › SVA_Diagnostics_and_PCA_Loadings.ipynb - Colab.pdf]

```
'''
Summary: This notebook is a highly specialized variation of the PCA/SVA pipeline that focuses on
preprocessing optimization and batch-correction diagnostics. Its defining feature is the manipulation
of the SVA design matrices—specifically transforming the continuous age parameter into binned categorical
factors (ageL, ageM, ageH) to better model latent covariates. It actively plots SVA residuals and runs
linear models to ensure surrogate variables are not inadvertently capturing the primary biological diagnosis,
before passing the cleanly corrected data into PCA loading extraction and subsequent Reactome pathway analysis.
'''
```

```
%load_ext rpy2.ipython
from google.colab import drive
drive.mount('/content/drive')
```

```
%%R -o DER_01 -o DER_03a -o DER_03b -o DER_tmp -o capstone

if (!requireNamespace("BiocManager", quietly = TRUE))
    install.packages("BiocManager")

BiocManager::install("WGCNA")
library(WGCNA)
#BiocManager::install("zFPKM")
#library(zFPKM)
#BiocManager::install("CancerSubtypes")
#library(CancerSubtypes)
#BiocManager::install("DESeq2")
#library(DESeq2)

install.packages("tidyverse")
library(tidyverse)

capstone_tmp <- read.csv("/content/drive/My Drive/datasets/PEC_capstone_data_map_clinical.csv")
capstone <- capstone_tmp[, c(1, 2, 6, 7, 8)]

#DER_tmp <- read.delim("/content/drive/My Drive/datasets/Controls_only_PEC_Gene_expression_matrix_normalized.txt")
DER_tmp <- read.delim("/content/drive/My Drive/datasets/DER-02_PEC_Gene_expression_matrix_TPM.txt")
#DER_03_tmp <- DER_tmp[,1]
#sql_peaks_tmp <- gsub("\\.\\d+", "", DER_03_tmp)
#DER_tmp[,1] <- gsub("c(", "", sql_peaks_tmp, fixed="TRUE")

DER_01 <- read.delim("/content/drive/My Drive/datasets/DER-02_PEC_Gene_expression_matrix_TPM.txt")
#DER_01 <- read.delim("/content/drive/My Drive/datasets/Controls_only_PEC_Gene_expression_matrix_normalized.txt")
DER_03 <- DER_01[,1]
sql_peaks <- gsub("\\.\\d+", "", DER_03)
DER_01[,1] <- gsub("c(", "", sql_peaks, fixed="TRUE")
DER_03a <- DER_01[,1]    #___ gene names
DER_03b <- colnames(DER_01)[-1] #___ sample names
DER_01 <- t(scale(t(DER_01[,-1])))   #_____scaling the data(otherwise all of the highly expressed genes
                                         #will cluster together even if they have different patterns
                                         #among the samples)

```

```
import numpy                      #___________________ Divide tissues and subjects to brain/peripheral
import numpy as np                                    # and CTL/SCZ, respectively.
import pandas as pd
from sklearn.preprocessing import MinMaxScaler
from sklearn.feature_selection import VarianceThreshold
from sklearn.preprocessing import StandardScaler
from scipy.stats import gaussian_kde

%R library(data.table)
%R BrainSeq <- fread('https://raw.githubusercontent.com/LieberInstitute/brainseq_phase2/master/BrainSeq_Phase2_phenotype_c
%R head(BrainSeq)
%R E <- readRDS('/content/drive/My Drive/datasets/expression.rds')
%R gdt <- readRDS('/content/drive/My Drive/datasets/gdt.rds')
%R samp <- fread('https://storage.googleapis.com/gtex_analysis_v7/annotations/GTEx_v7_Annotations_SampleAttributesDS.txt')
%R subj <- fread('https://storage.googleapis.com/gtex_analysis_v7/annotations/GTEx_v7_Annotations_SubjectPhenotypesDS.txt'
%R samp[, SUBJID := gsub('([^-]*)-([^-]*)-.*', '\\1-\\2', SAMPID)]
%R sdt <- merge(samp, subj, by='SUBJID')
%R sdt <- merge(samp, subj, by='SUBJID')[SAMPID %in% rownames(E)]
#______________________________________________________________________________________________________________

%R -o BrainSeq
%R -o DER_01
```

```
                                                        #________________________ Check for bias due to Tissue/Sex/Race.
#1.____BrainSeq[~BrainSeq.Region.str.contains("HIPPO")]      #___DLPFC/HIPPO   # Replace each line to the line below "3.__
#2.____BrainSeq[~BrainSeq.Race.str.contains("AA")]    #___CAUC/AA
#3.____BrainSeq[~BrainSeq.Sex.str.contains("F")]    #___M/F
BrSeq = pd.DataFrame(BrainSeq)
capstone = pd.DataFrame(capstone)

DER01 = pd.DataFrame(DER_01)
DER01.columns =  DER_03b
DER01 = np.transpose(DER01)
DER01.columns =  DER_03a
DER01 = np.transpose(DER01)

#____________________________________________________________________________I. BrainSeq DATASET(from Lieber Institute)

arr20 = np.array([])
arr20 = BrSeq['BrNum']
arr21 = np.array([])
arr21 = DER01.columns
xy21, x21_ind, y21_ind = np.intersect1d(arr20, arr21,
                                     return_indices=True)
data1 = []
for i in x21_ind:
  data1.append([BrSeq['BrNum'][i], BrSeq['Region'][i], BrSeq['Dx'][i],
                BrSeq['Sex'][i], BrSeq['Race'][i], BrSeq['RIN'][i], BrSeq['Age'][i]])
DER01_BrSeq = pd.DataFrame(data1, columns=['A', 'B', 'C', 'D', 'E', 'F', 'G'])
#DER01_BrSeq = DER01_BrSeq[~DER01_BrSeq.B.str.contains("HIPPO")]
#______________________
%R -o sdt
GTx = pd.DataFrame(sdt)

arr22 = np.array([])
arr22 = GTx['SUBJID']
xy22, x22_ind, y22_ind = np.intersect1d(arr22, arr21,
                                     return_indices=True)
data2 = []
for i in x22_ind:
  data2.append([GTx['SUBJID'][i], GTx['SMTSD'][i]])
df_GTx = pd.DataFrame(data2, columns=['A', 'B'])
#____________________________________________________
#FER01 = DER01.columns.to_series().str.contains('CMC')
#y23_ind = np.array([i for i, x in enumerate(FER01) if x])
#FER02 = DER01.columns.to_series().str.contains('Br')
#y24_ind = np.array([i for i, x in enumerate(FER02) if x])
#gtx_br = [*y23_ind, *y24_ind]

#gtx_br = [*y21_ind, *y23_ind, *y22_ind]
gtx_br = [*y21_ind, *y22_ind]

n13_train_scz = pd.DataFrame(DER01)
n14_train_scz = n13_train_scz.values[:,]
n15_train_scz = n14_train_scz[:,gtx_br]                                  #______ Filter samples

#___________________________________________________________________________II. CAPSTONE DATASET(from Kelsey)
'''
arr19a = np.array([])
arr19a = capstone['Synapse..individualID']
arr19b = np.array([])
arr19b = capstone['resource.psychencode.org..individualID']
arr21 = np.array([])
arr21 = DER01.columns
xy22, x22_ind, y22_ind = np.intersect1d(arr19b, arr21,
                                     return_indices=True)
gtx_cr = [*y22_ind]

data2 = []
for i in x22_ind:
  data2.append([capstone['resource.psychencode.org..individualID'][i],
                capstone['diagnosis'][i], capstone['sex'][i], capstone['ethnicity'][i]])
capstone_BrSeq2 = pd.DataFrame(data2, columns=['A', 'B', 'C', 'D'])

BipD = np.where(capstone_BrSeq2.B.str.contains("Bipolar Disorder"))
AutD = np.where(capstone_BrSeq2.B.str.contains("Autism Spectrum Disorder"))
AffD = np.where(capstone_BrSeq2.B.str.contains("Affective Disorder"))
BPD = np.where(capstone_BrSeq2.B.str.contains("BP"))

Dis_ind = [*BipD, *AutD, *AffD, *BPD]
```

```
Dis_ind_flat = [item for sublist in Dis_ind for item in sublist]

capstone_BrSeq2 = capstone_BrSeq2[~capstone_BrSeq2.B.str.contains("Bipolar Disorder")]
capstone_BrSeq2 = capstone_BrSeq2[~capstone_BrSeq2.B.str.contains("Autism Spectrum Disorder")]
capstone_BrSeq2 = capstone_BrSeq2[~capstone_BrSeq2.B.str.contains("Affective Disorder")]
capstone_BrSeq2 = capstone_BrSeq2[~capstone_BrSeq2.B.str.contains("BP")]

gj = []
for i in range(len(capstone_BrSeq2)):
  if len(list(capstone_BrSeq2['A'])[i])==0 :
    gj.append(i)
  if len(list(capstone_BrSeq2['B'])[i])==0 :
    gj.append(i)
  if len(list(capstone_BrSeq2['C'])[i])==0 :
    gj.append(i)
  if len(list(capstone_BrSeq2['D'])[i])==0 :
    gj.append(i)

res = []
[res.append(x) for x in gj if x not in res];

mask = np.ones(len(capstone_BrSeq2), bool)
mask[res] = False
capstone_BrSeq2_new = capstone_BrSeq2.iloc[mask]

len(capstone_BrSeq2_new)

n13_train_scz = pd.DataFrame(DER01)
n14_train_scz = n13_train_scz.values[:,]
n15_train_scz = n14_train_scz[:,gtx_cr]                                  #______ Filter samples
Dis_mask = np.ones(n15_train_scz.shape[1], bool)
Dis_mask[Dis_ind_flat] = False
n15_train_scz = n15_train_scz[:,Dis_mask]
n15_train_scz = n15_train_scz[:,mask]
'''
#________________________________________________________________________________

#n13_train_scz_min = n15_train_scz.astype(float)                          #______1. No transformation

###medianValue = median(logData);                                        #______2. Median centering data before log2-trans
###medianCtrData = logData-medianValue

n13_train_scz_min = np.log(n15_train_scz.astype(float)+1)               #______3. Log2 transformation

#n13_train_scz_min <- zFPKM(n15_train_scz.astype(float))                 #______4. Z-scale transformation (in R, from zFPK

n13_train_scz_min = np.transpose(n13_train_scz_min)                      #______ Shape the array as (samples, genes)

n13_train_scz_min[np.isnan(n13_train_scz_min)] = 0   #(57820, 414)       #______ Replace NAs with zeroes
pd.DataFrame(n13_train_scz_min).isnull().sum()

print(n13_train_scz_min.shape)
jdx = np.argwhere(np.all(n13_train_scz_min[..., :] == 0, axis=0))        #______ Remove zero columns
n13_train_scz_min = np.delete(n13_train_scz_min, jdx, axis=1)
print(n13_train_scz_min.shape)


f = VarianceThreshold().fit(n13_train_scz_min)
n13_train_scz_min = n13_train_scz_min[:, f.variances_ > 0.0]                                       #.___________________
print(n13_train_scz_min.shape)


kdx = jdx.tolist()                                                       #______ Final gene list after zero column removal
ldx = [item for sublist in kdx for item in sublist]
ER_TMM = []
DER_tmp.rename(columns = {'gene_id':'GeneName'}, inplace = True)
for i in range(len(DER_tmp['GeneName'][ldx])):
  ER_TMM.append(DER_tmp['GeneName'][ldx][i])
crr1 = np.array([])
crr1 = DER_tmp['GeneName']
crr2 = np.array([])
crr2 = ER_TMM
c22, c1_ind, c2_ind = np.intersect1d(crr1, crr2,
                     return_indices=True)
crr1[c1_ind] = 0
crr1 = [i for i in crr1 if i != 0]

```

```
crr1 = pd.DataFrame(crr1).iloc[f.variances_ > 0.0]                                                   #.___________________ 2
#print(pd.DataFrame(crr1).iloc[transform.get_support(indices=True)].shape)  #____ Gene Names(columns)


'''
for i in range(n13_train_scz_min.shape[0]):                                                #______ Z-FPKM(log2) transformation (in Python)
  n13_train_tmp = n13_train_scz_min[:,i]
  kernel = gaussian_kde(n13_train_tmp)
  xi = np.linspace(n13_train_tmp.min(), n13_train_tmp.max(), 100)
  yi = kernel.evaluate(xi)
  mu = xi[np.argmax(yi)]
  U = n13_train_scz_min[n13_train_scz_min > mu].mean()
  sigma = (U - mu) * np.sqrt(np.pi / 2)
  n13_train_scz_min[:,i] = (n13_train_tmp - mu) / sigma
'''

%R -i crr1
%R -i n13_train_scz_min                                                                   #______ Remove zero columns
#%R n13_train_scz_min <- n13_train_scz_min[, colSums(n13_train_scz_min != 0) > 0]
#%R print(dim(n13_train_scz_min))

#%R -i n13_train_scz_min                                                                   #______ z-transformation (in R)
#zscore<- function(x){
#    z<- (x - mean(x)) / sd(x)
#    return(z)
#}
#print(dim(n13_train_scz_min))
#n13_train_scz_min <- zscore(n13_train_scz_min)
#n13_train_scz_min <- n13_train_scz_min[, colSums(n13_train_scz_min != 0) > 0]
#print(dim(n13_train_scz_min))

##    PS: The FPKM counts are already normalised but are absolutely not suitable for
##        cross-sample comparisons, i.e., not suitable for differential expression analysis.
#______________________________________________________________________________

                                                                                    #_____ re-transform from log2(FPKM + z) ->
                                                                                    #                              -> FPKM ->
                                                                                    #                              -> zFPKM scores
#z=0.1
#exp.fpkm <- 2^expr
#exp.fpkm.original <- exp.fpkm - z
#exp.zfpkm <- zFPKM(exp.fpkm.original)
##filter out lowly expressed genes
#thres <- (ncol(exp.zfpkm) * 30) / 100
##filter all expression values that have absolute zfpkm score above 3.0 in more than 70% of the samples
#expr.zfpkm.filtered <- exp.zfpkm[(rowSums(abs(exp.zfpkm) > 3.0)) > thres, ]

#______________________________________________________________________________ Traits (CTL/SCZ)

data4 = []                                                  #I. BrainSeq DATASET(from Lieber Institute)
for i in DER01_BrSeq['B']:
  if i == 'DLPFC':
    k = 0
  if i == 'HIPPO':
    k = 1
  data4.append(k)
DER01_BrSeq6 = pd.DataFrame(data4, columns=['region'])

data4 = []
for i in DER01_BrSeq['C']:
  if i == 'Control':
    k = 0
  if i == 'Schizo':
    k = 1
  data4.append(k)
DER01_BrSeq1 = pd.DataFrame(data4, columns=['diag'])

data4 = []
for i in DER01_BrSeq['D']:
  if i == 'F':
    k = 0
  if i == 'M':
    k = 1
  data4.append(k)
DER01_BrSeq2 = pd.DataFrame(data4, columns=['sex'])

data4 = []
```

```
for i in DER01_BrSeq['E']:
  if i == 'CAUC':
    k = 1
  else:
    k = 0
  data4.append(k)
DER01_BrSeq3 = pd.DataFrame(data4, columns=['ethn'])

data4 = []
for i in DER01_BrSeq['F']:
  data4.append(i)
DER01_BrSeq4 = pd.DataFrame(data4, columns=['rin'])

data4 = []
for i in DER01_BrSeq['G']:
  data4.append(i)
DER01_BrSeq5 = pd.DataFrame(data4, columns=['age'])

DER01_BrSeq_Tot = pd.concat([DER01_BrSeq1, DER01_BrSeq2, DER01_BrSeq3,
                             DER01_BrSeq4, DER01_BrSeq5, DER01_BrSeq6], axis=1)

n13_trait_path1 = []
n13_trait_path1 = pd.DataFrame(DER01_BrSeq_Tot)

n13_trait_path = n13_trait_path1
%R -i n13_trait_path

#________________________________________________________________________________
'''
                                                                          #II. CAPSTONE DATASET(from Kelsey)
#pd.DataFrame(capstone_BrSeq2_new['B']).value_counts()
#pd.DataFrame(capstone_BrSeq2_new['C']).value_counts()
#pd.DataFrame(capstone_BrSeq2_new['D']).value_counts()

data4 = []
for i in capstone_BrSeq2_new['B']:
  if i == 'Control':
    k = 0
  if i == 'Schizophrenia':
    k = 1
  data4.append(k)
DER01_BrFin1 = pd.DataFrame(data4, columns=['diag'])

data4 = []
for i in capstone_BrSeq2_new['C']:
  if i == 'F':
    k = 0
  if i == 'M':
    k = 1
  data4.append(k)
DER01_BrFin2 = pd.DataFrame(data4, columns=['sex'])

data4 = []
for i in capstone_BrSeq2_new['D']:
  if i == 'CAUC':
    k = 1
  else:
    k = 0
  data4.append(k)
DER01_BrFin3 = pd.DataFrame(data4, columns=['ethn'])

DER01_BrFin = pd.concat([DER01_BrFin1, DER01_BrFin2, DER01_BrFin3], axis=1)

n13_trait_path1 = []
n13_trait_path1 = pd.DataFrame(DER01_BrFin)

n13_trait_path = n13_trait_path1
%R -i n13_trait_path
'''

del DER01_BrSeq                                                       #______ Clear unused variables
#del DER01_BrFin
del DER01
del n13_train_scz
del n14_train_scz
del n15_train_scz
```

```
#________________________________________________________________________________ Defining number of CTLs and SCZs
                                                                                # before removing outliers!!!!!!!!!
                                                                                # Use it as input to the PCA below


                                                                  #I. BrainSeq DATASET(from Lieber Institute)
DER01_BrSeq = pd.DataFrame(data1, columns=['A', 'B', 'C', 'D', 'E', 'F', 'G'])
#DER01_BrSeq = DER01_BrSeq[~DER01_BrSeq.B.str.contains("HIPPO")]

FER05 = DER01_BrSeq['C'].str.contains('Con')
z23_ind = np.array([i for i, x in enumerate(FER05) if x])

FER06 = DER01_BrSeq['C'].str.contains('Sc')
z24_ind = np.array([i for i, x in enumerate(FER06) if x])

gtx_all = [*z23_ind, *z24_ind]
#print(DER01_BrSeq.values[gtx_all])

n13_pca_scz_min = n13_train_scz_min[gtx_all,]
n13_pca_path = n13_trait_path.iloc[gtx_all]
#n13_pca_path = n13_trait_path['D'][gtx_all]
%R -i n13_pca_path

DER01_BrSeq['C'].value_counts()                                        #________ CTLs and SCZs

#________________________________________________________________________________
'''
                                                                   #II. CAPSTONE DATASET(from Kelsey)
FER05 = capstone_BrSeq2_new['B'].str.contains('Con')
z23_ind = np.array([i for i, x in enumerate(FER05) if x])

FER06 = capstone_BrSeq2_new['B'].str.contains('Sc')
z24_ind = np.array([i for i, x in enumerate(FER06) if x])

gtx_all = [*z23_ind, *z24_ind]
#print(DER01_BrFin1.values[gtx_all])

n13_pca_scz_min = n13_train_scz_min[gtx_all,]
n13_pca_path = n13_trait_path.iloc[gtx_all]
#n13_pca_path = n13_trait_path['diag'][gtx_all]
%R -i n13_pca_path

#DER01_BrFin['diag'].value_counts()                                        #________ CTLs and SCZs
capstone_BrSeq2_new['B'].value_counts()
'''
```

```
%%R -i n13_pca_scz_min                                                     #_____ Delete R variables, save files
                                                                                # for WGCNA analysis
rm(list=ls()[! ls() %in% c("n13_pca_scz_min", "n13_pca_path", "crr1")])
ls()

#save(n13_pca_scz_min, file = '/content/drive/My Drive/datasets/n13_pca_scz_min.RData')
#save(n13_pca_path, file = '/content/drive/My Drive/datasets/n13_pca_path.RData')

```

```
%%R -i n13_pca_scz_min
#________________________________________________________________________________ plot PCA before removing outliers!!!!!!!!!

library(ggplot2)

n13_pca_scz_min <- n13_pca_scz_min[ ,which(apply(n13_pca_scz_min, 2, var) != 0)]#_Remove zero variance columns from the new
genes=paste("gene",seq(1:dim(n13_pca_scz_min)[2]), sep="")
colnames(n13_pca_scz_min)=genes
row.names(n13_pca_scz_min)=c(paste0("ctl_",seq(1:261)), paste0("scz",seq(1:153)))  #___Python command: DER01_BrSeq['C'].val
condition1=rep(c("ctl"), each=261)
condition2=rep(c("scz"), each=153)
condition1 <- append(condition1, condition2)
length(condition1)

#pca_data=prcomp(n13_train_scz_min, center = TRUE, scale = TRUE)
#pca_data_perc=round(100*pca_data$sdev^2/sum(pca_data$sdev^2),1)
#df_pca_data=data.frame(PC1 = pca_data$x[,1], PC2 = pca_data$x[,2], sample = row.names(n13_train_scz_min), condition1=condi
#ggplot(df_pca_data, aes(PC1,PC2, color = condition1))+
#       geom_point(size=8)+
```

```
#      labs(x=paste0("PC1 (",pca_data_perc[1],")"), y=paste0("PC2 (",pca_data_perc[2],")"))

                                                                            #____ PCA for Z-scaled data
pca_data=prcomp(n13_pca_scz_min, center = TRUE, scale = F)
pca_data_perc=round(100*pca_data$sdev^2/sum(pca_data$sdev^2),1)
df_pca_data=data.frame(PC1 = pca_data$x[,1], PC2 = pca_data$x[,2], sample = row.names(n13_pca_scz_min), condition1=conditic

                                                                    #____________ Plot samples
ggplot(df_pca_data, aes(PC1,PC2, color = condition1))+
      geom_point(size=2)+
      labs(x=paste0("PC1 (",pca_data_perc[1],")"), y=paste0("PC2 (",pca_data_perc[2],")"))

                                                                    #____________ Plot genes
#ggplot(df_pca_data, aes(PC1,PC2, color = sample))+geom_point(size=2)+
#      labs(x=paste0("PC1 (",pca_data_perc[1],")"), y=paste0("PC2 (",pca_data_perc[2],")"))+
#      theme(legend.position = "none")

#df_pca_data[df_pca_data$PC1>300000,]                                        #____ How many patients/controls
#sum(df_pca_data$PC1>300000)                                                     #  as outliers in the PC1 axis

#________________________________________________________________________________ PCA using FactoMineR package

##install.packages("FactoMineR")
##library(FactoMineR)
##install.packages("factoextra")
##library(factoextra)
#pca_data <- as.data.frame(n13_train_scz_min)
#pca_data$group <- c(rep('ctl',261),rep('scz',153))
#pca <- PCA(pca_data[,1:(ncol(pca_data)-1)], graph = F)
#fviz_pca_ind(pca,
#         geom.ind = "point",
#         col.ind = pca_data$group,
#         palette = c("#00AFBB", "#E7B800"),
#         addEllipses = TRUE,
#         legend.title = "Groups")

```

```
#___________________________________WGCNA_______________________________________
```

```
#___________________________________WGCNA_______________________________________
```

```
#___________________________________WGCNA_______________________________________
```

```
%%R

sampleTree = hclust(dist(n13_pca_scz_min), method = "average");
par(cex = 0.6);
par(mar = c(0,4,2,0))
plot(sampleTree, main = "Sample clustering to detect outliers",
     sub="", xlab="", cex.lab = 1.5,cex.axis = 1.5, cex.main = 2)

abline(h = 160, col = "red");
clust = cutreeStatic(sampleTree, cutHeight = 160, minSize = 1)
table(clust)
keepSamples1 = (clust==1)
print(sum(keepSamples1))
keepSamples2 = (clust==2)
print(sum(keepSamples2))

keepSamples <- list()
keepSamples <- c(keepSamples, keepSamples1+keepSamples2)
length(keepSamples)

dim(n13_pca_scz_min)
datExpr = n13_pca_scz_min[which(keepSamples != 0),]
print(dim(datExpr))

nGenes <- ncol(datExpr)
nSamples <- nrow(datExpr)

datTraits <- n13_pca_path[which(keepSamples != 0),]
print(dim(datTraits))

```

```
#_______________________________________________________________________________ defining new number of CTLs and SCZs after
                                                                                  # Use it as input to the PCA below

                                        #I. BrainSeq DATASET(from Lieber Institute)
#DER01_BrSeq = pd.DataFrame(data1, columns=['A', 'B', 'C', 'D', 'E', 'F', 'G'])
#%R -i DER01_BrSeq
#%R -o DER02_BrSeq DER02_BrSeq <- DER01_BrSeq[which(keepSamples != 0),]
#pd.DataFrame(DER02_BrSeq['C']).value_counts()

%R -o datTraits
print(pd.DataFrame(datTraits['diag']).value_counts())

'''
                                        #II. CAPSTONE DATASET(from Kelsey)
%R -i capstone_BrSeq2_new
%R -o capstone_BrSeq3_new capstone_BrSeq3_new <- capstone_BrSeq2_new[which(keepSamples != 0),]
pd.DataFrame(capstone_BrSeq3_new['B']).value_counts()
'''
```

```
%%R
#_______________________________________________________________________________ plot PCA after removing outliers!!!!!!!!!!

library(ggplot2)

crr1 <- crr1[which(apply(datExpr, 2, var) != 0),]
datExpr <- datExpr[ ,which(apply(datExpr, 2, var) != 0)]#_Remove zero variance columns from the new dataset
save(datExpr, file = '/content/drive/My Drive/datasets/datExpr.RData')
save(crr1, file = '/content/drive/My Drive/datasets/crr1.RData')

genes=paste("gene",seq(1:dim(datExpr)[2]), sep="")
colnames(datExpr)=genes
row.names(datExpr)=c(paste0("ctl_",seq(1:246)), paste0("scz",seq(1:138)))  #___Python command: DER01_BrSeq['C'].value_count
condition1=rep(c("ctl"), each=246)
condition2=rep(c("scz"), each=138)
condition1 <- append(condition1, condition2)
length(condition1)

#pca_data=prcomp(datExpr, center = TRUE, scale = TRUE)
#pca_data_perc=round(100*pca_data$sdev^2/sum(pca_data$sdev^2),1)
#df_pca_data=data.frame(PC1 = pca_data$x[,1], PC2 = pca_data$x[,2], sample = row.names(datExpr), condition1=condition1)
#ggplot(df_pca_data, aes(PC1,PC2, color = condition1))+
#      geom_point(size=8)+
#      labs(x=paste0("PC1 (",pca_data_perc[1],")"), y=paste0("PC2 (",pca_data_perc[2],")"))

                                                                              #____ PCA for Z-scaled data
pca_data=prcomp(datExpr, center = TRUE, scale = F)
pca_data_perc=round(100*pca_data$sdev^2/sum(pca_data$sdev^2),1)
df_pca_data=data.frame(PC1 = pca_data$x[,1], PC2 = pca_data$x[,2], sample = row.names(datExpr), condition1=condition1)

                                                              #_____________Plot samples
ggplot(df_pca_data, aes(PC1,PC2, color = condition1))+
      geom_point(size=2)+
      labs(x=paste0("PC1 (",pca_data_perc[1],")"), y=paste0("PC2 (",pca_data_perc[2],")"))

                                                              #_____________Plot genes
#ggplot(df_pca_data, aes(PC1,PC2, color = sample))+geom_point(size=2)+
#      labs(x=paste0("PC1 (",pca_data_perc[1],")"), y=paste0("PC2 (",pca_data_perc[2],")"))+
#      theme(legend.position = "none")
#_______________________________________________________________________________ PCA using FactoMineR package

##install.packages("FactoMineR")
##library(FactoMineR)
##install.packages("factoextra")
##library(factoextra)
#pca_data <- as.data.frame(datExpr)
#pca_data$group <- c(rep('ctl',250),rep('scz',139))
#pca <- PCA(pca_data[,1:(ncol(pca_data)-1)], graph = F)
#fviz_pca_ind(pca,
#         geom.ind = "point",
#         col.ind = pca_data$group,
#         palette = c("#00AFBB", "#E7B800"),
#         addEllipses = TRUE,
#         legend.title = "Groups")
```

```
%%R
#________________________________________________________________________________ Matrix factorization (age parameter as thr

#load(file = '/content/drive/My Drive/datasets/datExpr.RData')
#load(file = '/content/drive/My Drive/datasets/datTraits.RData')
#load(file = '/content/drive/My Drive/datasets/crr1.RData')

datTraits <- n13_pca_path[which(keepSamples != 0),]

xs=quantile(datTraits$age,c(0,1/3,2/3,1))
xs[1]=xs[1]-.00005
datTraits <- datTraits %>% mutate(category=cut(age, breaks=xs,
  labels=c("low","middle","high"),ordered_result = TRUE))

datExpr = t(datExpr)
datTraits$diag <- as.factor(datTraits$diag)
datTraits$ethn <- as.factor(datTraits$ethn)
datTraits$sex <- as.factor(datTraits$sex)
datTraits$age <- as.factor(as.numeric(as.factor(datTraits$category)))
datTraits$region <- as.factor(datTraits$region)

out = binarizeCategoricalVariable(datTraits$age,
includePairwise = FALSE,
includeLevelVsAll = TRUE);
datTraits$ageL <- as.factor(out[,1])
datTraits$ageM <- as.factor(out[,2])
datTraits$ageH <- as.factor(out[,3])
colnames(datExpr) <- c(rownames(datTraits[,c(-4:-5,-7)] ))
rownames(datExpr) <- t(crr1)
datExpr = t(datExpr)

datTraits<-datTraits[,c(-4:-5,-7)]
print(head(datTraits))

```

```
%%R                                      #______________________________________ SVA analysis    (AG3/5)


if (!requireNamespace('BiocManager', quietly = TRUE))
    install.packages('BiocManager')

BiocManager::install('sva')
library(sva)

#mod0 = model.matrix(~as.factor(ethn)+as.factor(sex)+as.factor(age)+as.factor(region), data=datTraits)   #________________
#mod = model.matrix(~as.factor(diag)+as.factor(ethn)+as.factor(sex)+as.factor(age)+as.factor(region), data=datTraits)

mod0 = model.matrix(~as.factor(ethn)+as.factor(sex)+as.factor(ageL)+as.factor(ageH)+as.factor(region), data=datTraits)   #_
mod = model.matrix(~as.factor(diag)+as.factor(ethn)+as.factor(sex)+as.factor(ageL)+as.factor(ageH)+as.factor(region), data=

n.sv = num.sv(t(datExpr),mod,method="leek")
svobj = sva(t(datExpr),mod,mod0,n.sv=n.sv)

cleanY = function(y, mod, svobj) {
    X = cbind(mod, svobj)
    Hat = solve(t(X) %*% X) %*% t(X)
    beta = (Hat %*% t(y))
    rm(Hat)
    gc()
    P = ncol(mod)
    return(y - t(as.matrix(X[,-c(1:P)]) %*% beta[-c(1:P),]))
}

datExpr_cln <- cleanY(t(datExpr), mod, svobj$sv)

#____________________________________________________________________________________________________
#mod0 = model.matrix(~as.factor(Gender)+as.factor(Population), data=pheno)
#mod = model.matrix(~as.factor(inversion_genotype)+as.factor(Gender)+as.factor(Population), data=pheno)
#n.sv = num.sv(edata,mod,method="leek")
#svobj = sva(edata,mod,mod0,n.sv=n.sv)

```

```
%%R

fit1 = lm.fit(mod,datExpr)
modsv = cbind(mod,svobj$sv)
```

```
fitsv = lm.fit(modsv,datExpr)

plot(fitsv$coefficients[2,], fit1$coefficients[2,],col=2,
      xlab="SVA",ylab="linear model",xlim=c(-1,1),ylim=c(-1,1))
abline(c(0,1),col=1,lwd=3)

```

```
%%R

summary(lm(svobj$sv ~ datTraits$diag))

```

```
%%R

boxplot(svobj$sv[,3] ~ datTraits$diag)
points(svobj$sv[,3] ~ jitter(as.numeric(datTraits$diag)),col=as.numeric(datTraits$diag))

```

```
%%R
#________________________________________________________________________________ plot PCA after SVA analysis!!!!!!!!!!

datExpr_cln <- t(datExpr_cln)

library(ggplot2)

#crr1 <- crr1[which(apply(datExpr_cln, 2, var) != 0),]
#datExpr_cln <- datExpr_cln[ ,which(apply(datExpr_cln, 2, var) != 0)]#_Remove zero variance columns from the new dataset

genes=paste("gene",seq(1:dim(datExpr_cln)[2]), sep="")
colnames(datExpr_cln)=genes
row.names(datExpr_cln)=c(paste0("ctl_",seq(1:246)), paste0("scz",seq(1:138)))  #___Python command: DER01_BrSeq['C'].value_c
condition1=rep(c("ctl"), each=246)
condition2=rep(c("scz"), each=138)
condition1 <- append(condition1, condition2)
length(condition1)
                                                                            #____ PCA for Z-scaled data
pca_data=prcomp(datExpr_cln, center = TRUE, scale. = F)
pca_data_perc=round(100*pca_data$sdev^2/sum(pca_data$sdev^2),1)
df_pca_data=data.frame(PC1 = pca_data$x[,1], PC2 = pca_data$x[,2], sample = row.names(datExpr_cln), condition1=condition1)

                                                        #_____________Plot samples
ggplot(df_pca_data, aes(PC1,PC2, color = condition1))+
      geom_point(size=2)+
      labs(x=paste0("PC1 (",pca_data_perc[1],")"), y=paste0("PC2 (",pca_data_perc[2],")"))

```

```
%%R

library(devtools)
install_github("vqv/ggbiplot")
library(ggbiplot)
library(ggplot2)

# Plot the PCA plots
hvPCA1<-ggbiplot(pca_data,choices=c(1,2),scale=0,groups=datTraits$diag, ellipse=T,var.axes=F) + scale_color_manual(values=c
hvPCA2<-ggbiplot(pca_data,choices=c(1,2),scale=0,groups=datTraits$ethn, ellipse=T,var.axes=F) + scale_color_manual(values=c
hvPCA3<-ggbiplot(pca_data,choices=c(1,2),scale=0,groups=datTraits$sex, ellipse=T,var.axes=F) + scale_color_manual(values=c(
hvPCA4<-ggbiplot(pca_data,choices=c(1,2),scale=0,groups=datTraits$region, ellipse=T,var.axes=F) + scale_color_manual(values


#hvPCA5<-ggbiplot(pca_data,choices=c(1,2),scale=0,groups=datTraits$age, ellipse=T,var.axes=F) + scale_color_manual(values=c
#hvPCA5<-ggbiplot(pca_data,choices=c(1,2),scale=0,groups=datTraits$age, ellipse=T,var.axes=F) + scale_color_manual(values=c


```

```
%%R

# Show the diagnosis plot
hvPCA1
```

```
%%R

```

```
# Show the ethnicity plot
hvPCA2
```

```
%%R

# Show the sex plot
hvPCA3
```

```
%%R

# Show the region plot
hvPCA4
```

```
%%R

datTraits1 <- n13_pca_path[which(keepSamples != 0),]
xs=quantile(datTraits1$age,c(0,1/3,2/3,1))
xs[1]=xs[1]-.00005
datTraits1 <- datTraits1 %>% mutate(category=cut(age, breaks=xs,
  labels=c("low","middle","high"),ordered_result = TRUE))
datTraits1$diag <- as.factor(datTraits1$diag)
datTraits1$ethn <- as.factor(datTraits1$ethn)
datTraits1$sex <- as.factor(datTraits1$sex)
datTraits1$age <- as.factor(as.numeric(as.factor(datTraits1$category)))
datTraits1$region <- as.factor(datTraits1$region)

hvPCA5<-ggbiplot(pca_data,choices=c(1,2),scale=0,groups=datTraits1$age, ellipse=T,var.axes=F) + scale_color_manual(values=c

# Show the age plot
hvPCA5
```

```
%%R                                                    #___________________________________________________ age parameter as

datExpr <- datExpr_cln
datTraits <- n13_pca_path[which(keepSamples != 0),]

xs=quantile(datTraits$age,c(0,1/3,2/3,1))
xs[1]=xs[1]-.00005
datTraits <- datTraits %>% mutate(category=cut(age, breaks=xs,
  labels=c("low","middle","high"),ordered_result = TRUE))

datExpr = t(datExpr)
datTraits$diag <- as.factor(datTraits$diag)
datTraits$ethn <- as.factor(datTraits$ethn)
datTraits$sex <- as.factor(datTraits$sex)
datTraits$age <- as.factor(as.numeric(as.factor(datTraits$category)))
datTraits$region <- as.factor(datTraits$region)

out = binarizeCategoricalVariable(datTraits$age,
includePairwise = FALSE,
includeLevelVsAll = TRUE);
datTraits$ageL <- as.factor(out[,1])
datTraits$ageM <- as.factor(out[,2])
datTraits$ageH <- as.factor(out[,3])
colnames(datExpr) <- c(rownames(datTraits[,c(-4:-5,-7)] ))
rownames(datExpr) <- t(crr1)
datExpr = t(datExpr)

datTraits<-datTraits[,c(-4:-5,-7)]
print(head(datTraits))

```

```
%%R
#_______________________________________________________________________________ Additional PCA analysis

#load(file = '/content/drive/My Drive/datasets/m13_train_scz_min')
#load(file = '/content/drive/My Drive/datasets/m13_trait_path.RData')
#load(file = '/content/drive/My Drive/datasets/crr1.RData')

#m13_train_scz_min = t(m13_train_scz_min)
#colnames(m13_train_scz_min) <- c(rownames(m13_trait_path))
#rownames(m13_train_scz_min) <- t(crr1)
#m13_trait_path$diag <- as.factor(m13_trait_path$diag)
#m13_train_scz_min = t(m13_train_scz_min)
```

```
#_____________________________________________________________________________________ PRCOMP (& randomForest prediction)

install.packages("randomForest")
install.packages("caret")
library(caret)
library(randomForest)

colnames(datExpr) <- t(crr1)
#datTraits$diag <- as.factor(datTraits$diag)

set.seed(1234)
new_train_scz_min <- datExpr[sample(nrow(datExpr)),]
new_trait_path <- datTraits[sample(nrow(datTraits)),]
new_trait_path <- new_trait_path[match(rownames(new_train_scz_min), rownames(new_trait_path)),]
set.seed(NULL)

pca.train <- new_train_scz_min[1:(as.integer(0.7*nrow(new_train_scz_min))),]
pca.test <- new_train_scz_min[-(1:(as.integer(0.7*nrow(new_train_scz_min)))),]
pca_trait_train <- new_trait_path[1:(as.integer(0.7*nrow(new_trait_path))),]
pca_trait_test <- new_trait_path[-(1:(as.integer(0.7*nrow(new_trait_path)))),]
pca.test <- pca.test[ ,which(apply(pca.train, 2, var) != 0)]
pca.train <- pca.train[ ,which(apply(pca.train, 2, var) != 0)]
prin_comp <- prcomp(pca.train, scale. = F)

#std_dev <- prin_comp$sdev                                  #___________ Plots
#pr_var <- std_dev^2
#prop_varex <- pr_var/sum(pr_var)

#plot(prop_varex, xlab = "Principal Component",
#             ylab = "Proportion of Variance Explained",
#             type = "b")

#plot(cumsum(prop_varex), xlab = "Principal Component",
#              ylab = "Cumulative Proportion of Variance Explained",
#              type = "b")

                                                           #______randomForest
train.data <- data.frame(diag = pca_trait_train$diag, prin_comp$x)
train.data <- train.data[,1:31]

RFmodel_Fit <- randomForest(diag ~ .,data = train.data, method = "anova")
RFmodel_Fit
test.data <- predict(prin_comp, newdata = pca.test)
test.data <- as.data.frame(test.data)
test.data <- test.data[,1:30]
RFmodel_Fit.prediction <- predict(RFmodel_Fit, test.data)
print(confusionMatrix(RFmodel_Fit.prediction, pca_trait_test$diag)$overall[1])
print(RFmodel_Fit$confusion)

#_____________________________________________________________________________________ PCATOOLS

if (!requireNamespace('BiocManager', quietly = TRUE))
    install.packages('BiocManager')

BiocManager::install('PCAtools')
library(PCAtools)

#_______________________________________________________ PCATOOLS(various Plotloadings and ENSEMBL to SYMBOL translation)

organism = "org.Hs.eg.db"
BiocManager::install(organism, character.only = TRUE, force = TRUE)
library(organism, character.only = TRUE)

require(org.Hs.eg.db)
mapping <- mapIds(
  org.Hs.eg.db,
  keys = colnames(datExpr),
  column = 'SYMBOL',
  keytype = 'ENSEMBL')
colnames(datExpr) <- make.unique(ifelse(is.na(mapping), colnames(datExpr), mapping))

p <- pca(t(datExpr), metadata = datTraits, center = TRUE,
         scale = F, removeVar = 0.1)

```

```
%%R                                              #___________________________________________________ age parameter as

eigencorplot(p,                                                                                  #_ 3a.
components = getComponents(p, 1:6),
metavars = c("diag", "sex", "ethn", "ageL", "ageH"),
col = c('darkblue', 'blue2', 'black', 'red2', 'darkred'),
cexCorval = 0.7,
colCorval = 'white',
fontCorval = 2,
posLab = 'bottomleft',
rotLabX = 45,
posColKey = 'top',
cexLabColKey = 1.5,
scale = TRUE,
main = bquote(Principal ~ component ~ Pearson ~ r^2 ~ clinical ~ correlates),
plotRsquared = TRUE,
corFUN = 'pearson',
corUSE = 'pairwise.complete.obs',
corMultipleTestCorrection = 'BH',
signifSymbols = c('****', '***', '**', '*', ''),
signifCutpoints = c(0, 0.0001, 0.001, 0.01, 0.05, 1))
```

```
%%R

biplot(p, showLoadings = TRUE, labSize = 5, pointSize = 5, sizeLoadingsNames = 5)      #_ 1.

```

```
%%R

pairsplot(p)                                                                              #_ 2.

```

```
%%R

plotloadings(p,                                                                           #_ 5.
    components = getComponents(p, c(1,2,3,4)),
    rangeRetain = 0.1,
    labSize = 3.0,
    absolute = FALSE,
    title = 'Loadings plot',
    subtitle = 'Misc PCs',
    caption = 'Top 10% variables',
    shape = 23, shapeSizeRange = c(1, 16),
    drawConnectors = FALSE)

```

```
%%R

plotloadings(p,                                                                           #_ 6.
components = getComponents(p, c(1,2,3,4,5)),
rangeRetain = 0.001,
labSize = 3.0,
title = 'Loadings plot',
subtitle = 'PC1, PC2, PC3, PC4, PC5',
caption = 'Top 1% variables',
drawConnectors = TRUE)

```

```
%%R

prin_comp <- prcomp(datExpr, scale. = F)
expl_var <- prin_comp$sdev^2/sum(prin_comp$sdev^2)
barplot(expl_var[1:50], ylab="Variance",
        names.arg=paste0("PC",seq(1:50)), col="darkred")

N_perm <- 10
expl_var_perm <- matrix(NA, ncol = length(prin_comp$sdev), nrow = N_perm)
for(k in 1:N_perm)
{
  expr_perm <- apply(datExpr,2,sample)
  PC_perm <- prcomp(expr_perm, center=TRUE, scale=FALSE)
  expl_var_perm[k,] <- PC_perm$sdev^2/sum(PC_perm$sdev^2)
}
plot(expl_var[1:200]~seq(1:200), ylab="Variance",
```

```
    col="green", type='o', xlab="PC")
lines(colMeans(expl_var_perm)[1:200]~seq(1:200),col="red")
legend("topright", c("original data", "data permutation"),
       fill=c("green","red"), inset=0.02)

pval <- apply(t(expl_var_perm) >= expl_var,1,sum) / N_perm
plot(pval[1:200]~seq(1:200),col="darkred",type='o',
    xlab="PC",ylab="p-value")
optPC<-head(which(pval>=0.05),1)-1
mtext(paste0("OPTIMAL PC = ",optPC))
```

```
%%R

install.packages("tsne")
##install.packages("Rtsne")
library(tsne)
##library(Rtsne)
library(ggplot2)

nComponents <- 118
seed <- 1234
set.seed(seed)

dat.filter.BCV.tsne<-tsne(pca_data$x[,1:nComponents],perplexity=20,max_iter=2000,whiten = FALSE)

tsne_plot <- data.frame(x = dat.filter.BCV.tsne[,1], y = dat.filter.BCV.tsne[,2], col = datTraits$diag)
ggplot(tsne_plot) + geom_point(aes(x=x, y=y, color=col, size=1))
```

```
%%R                        #_____________________________________________________ Create dataframe with the top genes of the
                                                                                  # Use it as input to Reactome enrichment ana

ph_all = list()
pl_all = list()

p1h<-order(p$loadings[1], decreasing=T)[1:5]
p1l<-order(p$loadings[1], decreasing=F)[1:5]

p2h<-order(p$loadings[2], decreasing=T)[1:5]
p2l<-order(p$loadings[2], decreasing=F)[1:5]

p3h<-order(p$loadings[3], decreasing=T)[1:5]
p3l<-order(p$loadings[3], decreasing=F)[1:5]

p4h<-order(p$loadings[4], decreasing=T)[1:5]
p4l<-order(p$loadings[4], decreasing=F)[1:5]

p5h<-order(p$loadings[5], decreasing=T)[1:5]
p5l<-order(p$loadings[5], decreasing=F)[1:5]

ph_all[[1]] <- rownames(p$loadings[p1h,])
ph_all[[2]] <- rownames(p$loadings[p2h,])
ph_all[[3]] <- rownames(p$loadings[p3h,])
ph_all[[4]] <- rownames(p$loadings[p4h,])
ph_all[[5]] <- rownames(p$loadings[p5h,])

pl_all[[1]] <- rownames(p$loadings[p1l,])
pl_all[[2]] <- rownames(p$loadings[p2l,])
pl_all[[3]] <- rownames(p$loadings[p3l,])
pl_all[[4]] <- rownames(p$loadings[p4l,])
pl_all[[5]] <- rownames(p$loadings[p5l,])
```

```
%%R

if (!requireNamespace("BiocManager", quietly = TRUE))
    install.packages("BiocManager")

BiocManager::install("biomaRt", force = TRUE)
library(biomaRt)

mart <- useMart("ENSEMBL_MART_ENSEMBL")
mart <- useDataset("hsapiens_gene_ensembl", mart)

#devtools::install_github(c("GuangchuangYu/DOSE", "GuangchuangYu/enrichplot", "GuangchuangYu/GOSemSim", "eliocamp/ggnewscal

BiocManager::install("clusterProfiler")
```

```
library(clusterProfiler)

BiocManager::install("ReactomePA")
library(ReactomePA)

#organism = "org.Hs.eg.db"
#BiocManager::install(organism, character.only = TRUE)
#library(organism, character.only = TRUE)

```

```
%%R                 #____________________________________________________________ Reactome pathway gene set enrichment analy

ll = list()
PCAGeneSets_tmp = list()
                                                                                #________________________ 1.
PCAGeneSets_tmp <- list(ph_all[[1]],ph_all[[2]],ph_all[[3]],ph_all[[4]],ph_all[[5]],
                           pl_all[[1]],pl_all[[2]],pl_all[[3]],pl_all[[4]],pl_all[[5]])

#pe1 = list()                                                                   #________________________ 2.
#pe2 = list()
#pe3 = list()
#pe4 = list()
#pe5 = list()
#pe1[[1]]<-ph_all[[1]]
#pe1[[2]]<-pl_all[[1]]
#pe2[[1]]<-ph_all[[2]]
#pe2[[2]]<-pl_all[[2]]
#pe3[[1]]<-ph_all[[3]]
#pe3[[2]]<-pl_all[[3]]
#pe4[[1]]<-ph_all[[4]]
#pe4[[2]]<-pl_all[[4]]
#pe5[[1]]<-ph_all[[5]]
#pe5[[2]]<-pl_all[[5]]
#PCAGeneSets_tmp <- list(unlist(pe1),unlist(pe2),unlist(pe3),unlist(pe4),unlist(pe5))

PCAGeneSets_entrez_tmp = list()
PCAGeneSets_entrez_tmp<-lapply(PCAGeneSets_tmp,function(x){
  bitr(x,fromType="SYMBOL",toType="ENTREZID",OrgDb="org.Hs.eg.db")$ENTREZID})

for (i in 1:length(PCAGeneSets_entrez_tmp)){
print("___________________________IV. enrichPathway_________________________________")
l6 <- enrichPathway(gene = as.character(unique(sort(PCAGeneSets_entrez_tmp[[i]]))),
                 pvalueCutoff = 0.05,
                 qvalueCutoff  = 0.1,
                 readable = TRUE)
if (length(l6$ID) == 0) {
ll[[i]] <- "NULL"
}
else{
ll[[i]] <- data.frame(
 i,
 l6$ID,
 l6$Description,
 l6$GeneRatio,
 l6$BgRatio,
 l6$pvalue,
 l6$p.adjust,
 l6$qvalue,
 l6$geneID,
 l6$Count
)
colnames(ll[[i]]) <- c("Community#", "ID", "Description", "GeneRatio",
                           "BgRatio", "pvalue", "p.adjust", "qvalue",
                           "geneID","Count")
options(scipen=999)
print(ll[[i]])
print("#######################################################################################################################
}
}

```
