# Supplementary material for: Exploration of schizophrenia-associated gene modules using graph theory, co-expression networks, and dimensionality reduction
Source: PLoS One. 2026 Apr 15;21(4):e0346663. doi: 10.1371/journal.pone.0346663 (PMC13082716; doi:10.1371/journal.pone.0346663)
Supplement: S1 Code — This archive contains four distinct Jupyter Notebooks used for the analysis: SVA_Diagnostics_and_PCA_Loadings.ipynb, PCA_Variance_Driven_Reactome_Enrichment.ipynb, WGCNA_Module_Functional_Enrichment.ipynb, and igraph_MST_Topological_Centrality.ipynb. (ZIP) [file pone.0346663.s008.zip › igraph_MST_Topological_Centrality.ipynb - Colab.pdf]

```
'''
Summary: This notebook abandons the dense WGCNA approach to construct a sparse, topological gene regulatory network
using the igraph package. By applying a dynamic signed threshold to a Spearman correlation matrix and executing
Prim's algorithm on the raw weights, it forces the construction of a Minimum Spanning Tree (MST). It then isolates
topological bottlenecks by stripping edge weights during community detection, calculates formal mathematical centrality
metrics (degree, closeness, betweenness, and eigenvector), and subsets the highest-ranking "bottleneck" genes from each
community for targeted GO and KEGG enrichment.
'''
```

```
%load_ext rpy2.ipython
from google.colab import drive
drive.mount('/content/drive')
```

```
%%R -o Idx_new -o DER_01 -o DER_03a -o DER_03b -o DER_tmp -o capstone

install.packages("R.matlab")
library(R.matlab)
library(dplyr)

DER_tmp <- read.delim("/content/drive/My Drive/datasets/DER-02_PEC_Gene_expression_matrix_TPM.txt")
capstone_tmp <- read.csv("/content/drive/My Drive/datasets/PEC_capstone_data_map_clinical.csv")
capstone <- capstone_tmp[, c(1, 2, 6, 7, 8)]

DER_01 <- read.delim("/content/drive/My Drive/datasets/DER-02_PEC_Gene_expression_matrix_TPM.txt")
DER_01[,1] <- gsub("c(", "", gsub("\\.\\d+", "", DER_01[,1]), fixed="TRUE")

scz_data1 <- readMat("/content/drive/My Drive/datasets/scz_data1.mat")

scz_data1f <- data.frame("X.geneIds"=matrix(unlist(scz_data1$X.geneIds), nrow=length(scz_data1$X.geneIds), byrow=TRUE),strin
scz_data1f$X.geneIds <- as.character(scz_data1f$X.geneIds)
scz_data1f$X.geneIds <- sub("[.][0-9]*","",scz_data1f$X.geneIds)
genes <-  scz_data1f$X.geneIds
crs3 <- list()
crs3 <- DER_01[DER_01[,1] %in% genes,1]
Idx_new <- which(DER_01[,1] %in% genes)

install.packages("tidyverse")
library(tidyverse)

DER_03a <- DER_01[,1]    #___ gene names
DER_03b <- colnames(DER_01)[-1] #___ sample names
DER_01 <- t(scale(t(DER_01[,-1])))   #_____scaling the data(otherwise all of the highly expressed genes
                                         #will cluster together even if they have different patterns
                                         #among the samples)
library(data.table)
BrainSeq <- fread('https://raw.githubusercontent.com/LieberInstitute/brainseq_phase2/master/BrainSeq_Phase2_phenotype_data_s
head(BrainSeq)

```

```
import numpy
import numpy as np
import pandas as pd

%R DEX_Genes <- read.csv('/content/drive/My Drive/datasets/DER-13_Disorder_DEX_Genes.csv')
%R dim(DEX_Genes)
%R DEX_SCZ <- DEX_Genes[grep("SCZ", DEX_Genes$Disorder.DGE_RegulationDirection), ]
%R dim(DEX_SCZ)
%R -o DEX_SCZ
arr3 = np.array([])
arr3 = DEX_SCZ.values[:,1]
seen = set()  #_______ remove duplicates
uniq = []
for x in arr3:
    if x not in seen:
        uniq.append(x)
        seen.add(x)             #_____________________(4821,)
if (len(DEX_SCZ) == len(uniq)):
  print("There are no duplicates")

arr4 = np.array([])
arr4 = DER_03a
xz2, z1_ind, z2_ind = np.intersect1d(arr3, arr4,
```

```
                                       return_indices=True)

```

```
import numpy                      #___________________ Divide tissues and subjects to brain/peripheral
import numpy as np                                  # and CTL/SCZ, respectively.
import pandas as pd
from sklearn.preprocessing import MinMaxScaler
from sklearn.feature_selection import VarianceThreshold
from sklearn.preprocessing import StandardScaler
from scipy.stats import gaussian_kde

%R library(data.table)
%R BrainSeq <- fread('https://raw.githubusercontent.com/LieberInstitute/brainseq_phase2/master/BrainSeq_Phase2_phenotype_c
%R head(BrainSeq)
%R E <- readRDS('/content/drive/My Drive/datasets/expression.rds')
%R gdt <- readRDS('/content/drive/My Drive/datasets/gdt.rds')
%R samp <- fread('https://storage.googleapis.com/gtex_analysis_v7/annotations/GTEx_v7_Annotations_SampleAttributesDS.txt')
%R subj <- fread('https://storage.googleapis.com/gtex_analysis_v7/annotations/GTEx_v7_Annotations_SubjectPhenotypesDS.txt'
%R samp[, SUBJID := gsub('([^-]*)-([^-]*)-.*', '\\1-\\2', SAMPID)]
%R sdt <- merge(samp, subj, by='SUBJID')
%R sdt <- merge(samp, subj, by='SUBJID')[SAMPID %in% rownames(E)]
#____________________________________________________________________________________________________________

%R -o BrainSeq
%R -o DER_01
                                                #________________________ Check for bias due to Tissue/Sex/Race.
#1.____BrainSeq[~BrainSeq.Region.str.contains("HIPPO")]      #___DLPFC/HIPPO   # Replace each line to the line below "3.__
#2.____BrainSeq[~BrainSeq.Race.str.contains("AA")]    #___CAUC/AA
#3.____BrainSeq[~BrainSeq.Sex.str.contains("F")]    #___M/F
BrSeq = pd.DataFrame(BrainSeq)
#capstone = pd.DataFrame(capstone)

DER01 = pd.DataFrame(DER_01)
DER01.columns =  DER_03b
DER01 = np.transpose(DER01)
DER01.columns =  DER_03a
DER01 = np.transpose(DER01)

#______________________________________________________________________________I. BrainSeq DATASET(from Lieber Institute)

arr20 = np.array([])
arr20 = BrSeq['BrNum']
arr21 = np.array([])
arr21 = DER01.columns
xy21, x21_ind, y21_ind = np.intersect1d(arr20, arr21,
                                     return_indices=True)
data1 = []
for i in x21_ind:
  data1.append([BrSeq['BrNum'][i], BrSeq['Region'][i], BrSeq['Dx'][i],
                BrSeq['Sex'][i], BrSeq['Race'][i], BrSeq['RIN'][i], BrSeq['Age'][i]])
DER01_BrSeq = pd.DataFrame(data1, columns=['A', 'B', 'C', 'D', 'E', 'F', 'G'])
#DER01_BrSeq = DER01_BrSeq[~DER01_BrSeq.B.str.contains("HIPPO")]
#______________________
%R -o sdt
GTx = pd.DataFrame(sdt)

arr22 = np.array([])
arr22 = GTx['SUBJID']
xy22, x22_ind, y22_ind = np.intersect1d(arr22, arr21,
                                     return_indices=True)
data2 = []
for i in x22_ind:
  data2.append([GTx['SUBJID'][i], GTx['SMTSD'][i]])
df_GTx = pd.DataFrame(data2, columns=['A', 'B'])
#____________________________________________________
#FER01 = DER01.columns.to_series().str.contains('CMC')
#y23_ind = np.array([i for i, x in enumerate(FER01) if x])
#FER02 = DER01.columns.to_series().str.contains('Br')
#y24_ind = np.array([i for i, x in enumerate(FER02) if x])
#gtx_br = [*y23_ind, *y24_ind]

#gtx_br = [*y21_ind, *y23_ind, *y22_ind]
gtx_br = [*y21_ind, *y22_ind]

n13_train_scz = pd.DataFrame(DER01)
n14_train_scz = n13_train_scz.values[list(z2_ind),]                     #______ Filter rows by DEGs or (igraph)...(1/2)
```

```
n15_train_scz = n14_train_scz[:,gtx_br]                                  #______ Filter samples

#________________________________________________________________________________

#n13_train_scz_min = n15_train_scz.astype(float)                          #______1. No transformation

###medianValue = median(logData);                                        #______2. Median centering data before log2-trans
###medianCtrData = logData-medianValue

n13_train_scz_min = np.log(n15_train_scz.astype(float)+1)               #______3. Log2 transformation

#n13_train_scz_min <- zFPKM(n15_train_scz.astype(float))                 #______4. Z-scale transformation (in R, from zFPK

n13_train_scz_min = np.transpose(n13_train_scz_min)                      #______ Shape the array as (samples, genes)

n13_train_scz_min[np.isnan(n13_train_scz_min)] = 0   #(57820, 414)       #______ Replace NAs with zeroes
pd.DataFrame(n13_train_scz_min).isnull().sum()

print(n13_train_scz_min.shape)
jdx = np.argwhere(np.all(n13_train_scz_min[..., :] == 0, axis=0))        #______ Remove zero columns
n13_train_scz_min = np.delete(n13_train_scz_min, jdx, axis=1)
print(n13_train_scz_min.shape)



#n13_train_scz_min = pd.DataFrame(n13_train_scz_min)
#transform = VarianceThreshold(0)                                          #______ Remove genes with variance
print(n13_train_scz_min.shape)                                                 # over a threshold
DER_04a = np.array(DER_03a)[list(z2_ind)]    #______ Filter rows by DEGs or (igraph)...(2/2)
DER_05a = np.delete(DER_04a, jdx)
#DER_06a = DER_05a[VarianceThreshold().fit(n13_train_scz_min).get_support()] #___ Final Gene headers!!!!
#n13_train_scz_min = n13_train_scz_min.iloc[:,VarianceThreshold().fit(n13_train_scz_min).get_support()]

f = VarianceThreshold().fit(n13_train_scz_min)
DER_06a = DER_05a[f.variances_ > 0.0] #___ Final Gene headers!!!!
print(DER_06a.shape)
n13_train_scz_min = n13_train_scz_min[:, f.variances_ > 0.0]
print(n13_train_scz_min.shape)

%R -i n13_train_scz_min                                                 #______ Remove zero columns

#________________________________________________________________________________ Traits (CTL/SCZ)

data4 = []                                            #I. BrainSeq DATASET(from Lieber Institute)
for i in DER01_BrSeq['B']:
  if i == 'DLPFC':
    k = 0
  if i == 'HIPPO':
    k = 1
  data4.append(k)
DER01_BrSeq6 = pd.DataFrame(data4, columns=['region'])

data4 = []
for i in DER01_BrSeq['C']:
  if i == 'Control':
    k = 0
  if i == 'Schizo':
    k = 1
  data4.append(k)
DER01_BrSeq1 = pd.DataFrame(data4, columns=['diag'])

data4 = []
for i in DER01_BrSeq['D']:
  if i == 'F':
    k = 0
  if i == 'M':
    k = 1
  data4.append(k)
DER01_BrSeq2 = pd.DataFrame(data4, columns=['sex'])

data4 = []
for i in DER01_BrSeq['E']:
  if i == 'CAUC':
    k = 1
  else:
    k = 0
  data4.append(k)
```

```
DER01_BrSeq3 = pd.DataFrame(data4, columns=['ethn'])

data4 = []
for i in DER01_BrSeq['F']:
  data4.append(i)
DER01_BrSeq4 = pd.DataFrame(data4, columns=['rin'])

data4 = []
for i in DER01_BrSeq['G']:
  data4.append(i)
DER01_BrSeq5 = pd.DataFrame(data4, columns=['age'])

DER01_BrSeq_Tot = pd.concat([DER01_BrSeq1, DER01_BrSeq2, DER01_BrSeq3,
                             DER01_BrSeq4, DER01_BrSeq5, DER01_BrSeq6], axis=1)

n13_trait_path1 = []
n13_trait_path1 = pd.DataFrame(DER01_BrSeq_Tot)

n13_trait_path = n13_trait_path1
%R -i n13_trait_path

del DER01_BrSeq                                                             #______ Clear unused variables
#del DER01_BrFin
del DER01
del n13_train_scz
del n14_train_scz
del n15_train_scz

```

```
#________________________________________________________________________________ Defining number of CTLs and SCZs
                                                                                 # before removing outliers!!!!!!!!!
                                                                                 # Use it as input to the PCA below


                                                                   #I. BrainSeq DATASET(from Lieber Institute)
DER01_BrSeq = pd.DataFrame(data1, columns=['A', 'B', 'C', 'D', 'E', 'F', 'G'])
#DER01_BrSeq = DER01_BrSeq[~DER01_BrSeq.B.str.contains("HIPPO")]

FER05 = DER01_BrSeq['C'].str.contains('Con')
z23_ind = np.array([i for i, x in enumerate(FER05) if x])

FER06 = DER01_BrSeq['C'].str.contains('Sc')
z24_ind = np.array([i for i, x in enumerate(FER06) if x])

gtx_all = [*z23_ind, *z24_ind]
#print(DER01_BrSeq.values[gtx_all])

n13_pca_scz_min = n13_train_scz_min[gtx_all,]
n13_pca_path = n13_trait_path.iloc[gtx_all]
#n13_pca_path = n13_trait_path['D'][gtx_all]
%R -i n13_pca_path

DER01_BrSeq['C'].value_counts()                                        #_________ CTLs and SCZs

```

```
%%R -i n13_pca_scz_min
#________________________________________________________________________________ plot PCA before removing outliers!!!!!!!!!

library(ggplot2)

n13_pca_scz_min <- n13_pca_scz_min[ ,which(apply(n13_pca_scz_min, 2, var) != 0)]#_Remove zero variance columns from the new
genes=paste("gene",seq(1:dim(n13_pca_scz_min)[2]), sep="")
colnames(n13_pca_scz_min)=genes
row.names(n13_pca_scz_min)=c(paste0("ctl_",seq(1:261)), paste0("scz",seq(1:153)))  #___Python command: DER01_BrSeq['C'].val
condition1=rep(c("ctl"), each=261)
condition2=rep(c("scz"), each=153)
condition1 <- append(condition1, condition2)
length(condition1)

#pca_data=prcomp(n13_train_scz_min, center = TRUE, scale = TRUE)
#pca_data_perc=round(100*pca_data$sdev^2/sum(pca_data$sdev^2),1)
#df_pca_data=data.frame(PC1 = pca_data$x[,1], PC2 = pca_data$x[,2], sample = row.names(n13_train_scz_min), condition1=condi
#ggplot(df_pca_data, aes(PC1,PC2, color = condition1))+
#      geom_point(size=8)+
#      labs(x=paste0("PC1 (",pca_data_perc[1],")"), y=paste0("PC2 (",pca_data_perc[2],")"))

```

```
                                                                      #____ PCA for Z-scaled data
pca_data=prcomp(n13_pca_scz_min, center = TRUE, scale = TRUE)
pca_data_perc=round(100*pca_data$sdev^2/sum(pca_data$sdev^2),1)
df_pca_data=data.frame(PC1 = pca_data$x[,1], PC2 = pca_data$x[,2], sample = row.names(n13_pca_scz_min), condition1=conditic

                                                              #____________ Plot samples
ggplot(df_pca_data, aes(PC1,PC2, color = condition1))+
      geom_point(size=2)+
      labs(x=paste0("PC1 (",pca_data_perc[1],")"), y=paste0("PC2 (",pca_data_perc[2],")"))

```

```
%%R -i n13_pca_path

datExpr = n13_pca_scz_min

nGenes <- ncol(datExpr)
nSamples <- nrow(datExpr)

datTraits <- n13_pca_path

rm(list=ls()[! ls() %in% c("datExpr", "nGenes", "datTraits", "nSamples")])
ls()

```

```
%%R

# Setting the colors to match the regional colors
region_labels <- RColorBrewer::brewer.pal(6, "Set1")[1:3]

# Making sure the regional colors match what has been used througout and making a column with the hex codes.

datTraits <- datTraits %>%
  mutate(cell.names = row.names(datTraits)) %>%
  mutate(x = ifelse(datTraits$sex == "1", region_labels[1],
                    ifelse(datTraits$sex == "0", region_labels[2], NA)))

datTraits <- datTraits %>%
  mutate(cell.names = row.names(datTraits)) %>%
  mutate(x = ifelse(datTraits$ethn == "1", region_labels[1],
                    ifelse(datTraits$ethn == "0", region_labels[2], NA)))

datTraits <- datTraits %>%
  mutate(cell.names = row.names(datTraits)) %>%
  mutate(x = ifelse(datTraits$region == "1", region_labels[1],
                    ifelse(datTraits$region == "0", region_labels[2], NA)))

datTraits <- datTraits %>%
  mutate(cell.names = row.names(datTraits)) %>%
  mutate(x = ifelse(datTraits$diag == "1", region_labels[1],
                    ifelse(datTraits$diag == "0", region_labels[2], NA)))

xs=quantile(datTraits$age,c(0,1/3,2/3,1))
xs[1]=xs[1]-.00005
datTraits <- datTraits %>% mutate(category=cut(age, breaks=xs,
  labels=c("low","middle","high"),ordered_result = TRUE))
boxplot(datTraits$age~datTraits$category,col=3:5)

datExpr <- datExpr[ ,which(apply(datExpr, 2, var) != 0)]
datExpr = t(datExpr)
colnames(datExpr) <- c(rownames(datTraits ))

datTraits$diag <- as.factor(datTraits$diag)
datTraits$ethn <- as.factor(datTraits$ethn)
datTraits$sex <- as.factor(datTraits$sex)
datTraits$age <- as.factor(datTraits$category)
datTraits$region <- as.factor(datTraits$region)

datExpr = t(datExpr)
```

```
%%R                                         #_______________________________________ SVA analysis

if (!requireNamespace('BiocManager', quietly = TRUE))
    install.packages('BiocManager')

BiocManager::install('sva')
```

```
library(sva)

mod0 = model.matrix(~as.factor(ethn)+as.factor(sex)+as.factor(age)+as.factor(region), data=datTraits)
mod = model.matrix(~as.factor(diag)+as.factor(ethn)+as.factor(sex)+as.factor(age)+as.factor(region), data=datTraits)
n.sv = num.sv(t(datExpr),mod,method="leek")
svobj = sva(t(datExpr),mod,mod0,n.sv=n.sv)

cleanY = function(y, mod, svobj) {
    X = cbind(mod, svobj)
    Hat = solve(t(X) %*% X) %*% t(X)
    beta = (Hat %*% t(y))
    rm(Hat)
    gc()
    P = ncol(mod)
    return(y - t(as.matrix(X[,-c(1:P)]) %*% beta[-c(1:P),]))
}

datExpr_cln <- cleanY(t(datExpr), mod, svobj$sv)
datExpr_cln <- t(datExpr_cln)
datExpr = datExpr_cln
nGenes <- ncol(datExpr)
nSamples <- nrow(datExpr)

```

```
%%R

rm(list=ls()[! ls() %in% c("datExpr", "nGenes", "datTraits", "nSamples")])
ls()

```

```
%%R
#________________________________________________________________________________ plot PCA after removing outliers!!!!!!!!!!

library(ggplot2)

row.names(datExpr)=c(paste0("ctl_",seq(1:261)), paste0("scz",seq(1:153)))  #___Python command: DER01_BrSeq['C'].value_count
condition1=rep(c("ctl"), each=261)
condition2=rep(c("scz"), each=153)
condition1 <- append(condition1, condition2)
length(condition1)
                                                                            #____ PCA for Z-scaled data
pca_data=prcomp(datExpr, center = TRUE, scale = TRUE)
pca_data_perc=round(100*pca_data$sdev^2/sum(pca_data$sdev^2),1)
df_pca_data=data.frame(PC1 = pca_data$x[,1], PC2 = pca_data$x[,2], sample = row.names(datExpr), condition1=condition1)

                                                        #_____________Plot samples
ggplot(df_pca_data, aes(PC1,PC2, color = condition1))+
      geom_point(size=2)+
      labs(x=paste0("PC1 (",pca_data_perc[1],")"), y=paste0("PC2 (",pca_data_perc[2],")"))

```

```
%%R -i DER_06a

colnames(datExpr) <- DER_06a

#Corr_ctl2 <- as.matrix(as.dist(cor(datExpr[1:261,], method="spearman")))   #_________ Correlations for CTL network
Corr_ctl2 <- as.matrix(as.dist(cor(datExpr[262:414,], method="spearman"))) #_________ Correlations for SCZ network
                                                                          #_____method="pearson", "kendall", "spearman"

#save(Corr_ctl2, file="/content/drive/My Drive/datasets/Corr_ctl_net.Rdata")
#save(Corr_ctl2, file="/content/drive/My Drive/datasets/Corr_scz_net.Rdata")

#____________________________________________________________________________________________________________________
#load("/content/drive/My Drive/datasets/Corr_ctl_net.Rdata")
#load("/content/drive/My Drive/datasets/Corr_scz_net.Rdata")
Corr_ctl2 <- ((Corr_ctl2-min(Corr_ctl2))/(max(Corr_ctl2)-min(Corr_ctl2)))*2 - 1

set.seed(1234)

install.packages("igraph")
library(igraph)
#__________________________________
ctl1 <- graph.adjacency(
  Corr_ctl2,
  mode="undirected",
```

```
  weighted=TRUE,
  diag=FALSE)
ctl1 <- delete_edges(ctl1, E(ctl1)[which(E(ctl1)$weight < max(E(ctl1)$weight)*0.8 & E(ctl1)$weight > min(E(ctl1)$weight)*0.
ctl1 <- igraph::simplify(ctl1, remove.multiple=TRUE, remove.loops=FALSE)
#save(ctl1, file="/content/drive/My Drive/datasets/ctl_net.Rdata")

E(ctl1)[which(E(ctl1)$weight<0)]$color <- "darkblue"
E(ctl1)[which(E(ctl1)$weight>0)]$color <- "darkred"

G2 <- igraph::delete.vertices(ctl1, igraph::graph.strength(ctl1)==0)

ctl1 <- induced_subgraph(
  G2, V(G2)[components(G2)$membership == which.max(components(G2)$csize)]
)
print(is.connected(ctl1))

V(ctl1)$shape <- "sphere"
V(ctl1)$color <- "skyblue"
V(ctl1)$vertex.frame.color <- "white"

M2.subgraph <- mst(ctl1, algorithm="prim")
print(is.connected(M2.subgraph))

E(M2.subgraph)$weight <- abs(E(M2.subgraph)$weight)

```

```
%%R                                                        #______ RUN FIRST
'''
M2.subgraph <- induced_subgraph(
  G2, V(G2)[components(G2)$membership == which.max(components(G2)$csize)]
)
print(is.connected(M2.subgraph))

V(M2.subgraph)$shape <- "sphere"
V(M2.subgraph)$color <- "skyblue"
V(M2.subgraph)$vertex.frame.color <- "white"

E(M2.subgraph)$weight <- abs(E(M2.subgraph)$weight)
'''
```

```
%%R -i n13_pca_scz_min                                                      #______ RUN SECOND
'''
#______________________________________________________________________________ I. Normality tests

#x = datExpr[1:261,]; qqnorm(x); qqline(x,col='red')           #_________ CTL network
#hist(x)
#boxplot(x)


x = datExpr[262:414,]; qqnorm(x); qqline(x,col='red')        #_________ SCZ network
hist(x)
boxplot(x)

#______________________________________________________________________________ II. CTL and SCZ Networks Various Distribut

hist(igraph::degree(M2.subgraph), col="lightblue", breaks=10,#xlim=c(0,400),
    xlab="Vertex Degree", ylab="Frequency", main="Histogram of node degree")

hist(igraph::closeness(M2.subgraph), col="lightblue", breaks=10,#xlim=c(0,400),
    xlab="Closeness", ylab="Frequency", main="Histogram of node closeness")

hist(igraph::betweenness(M2.subgraph), col="lightblue", breaks=10,#xlim=c(0,400),
    xlab="Betweenness", ylab="Frequency", main="Histogram of node betweenness")

hist(igraph::evcent(M2.subgraph)$vector, col="lightblue", breaks=10,#xlim=c(0,400),
    xlab="EigenVector", ylab="Frequency", main="Histogram of node eigenVector")
'''
```

```
%%R

install.packages("GGally")
install.packages("ggplot2")
install.packages("ggdendro")
library(GGally)
library(ggplot2)
```

```
library(ggdendro)

#M2_LGL = layout_nicely(M2.subgraph)
#M2_LGL = layout.fruchterman.reingold(M2.subgraph)
M2_LGL = layout_with_lgl(M2.subgraph)
plot(M2.subgraph, vertex.size = 2, vertex.frame.color = NULL,layout=M2_LGL, asp=FALSE,
vertex.label=NA, edge.curved=TRUE, edge.arrow.size=0.1, vertex.label.dist = 0.5, edge.width = 0.5)

#_____________________________________________
#M2.subgraph.communities <- cluster_louvain(as.undirected(M2.subgraph), weights = NULL)         #weights = E(M2.subgraph)$w
#louvain_sizesComm <- sizes(M2.subgraph.communities)
#louvain_numComm <- length(louvain_sizesComm)
#louvain_modularity <- modularity(M2.subgraph.communities)
#print(louvain_numComm)
##print(louvain_sizesComm)
#print(louvain_modularity)

M2.subgraph.communities <- edge.betweenness.community(as.undirected(M2.subgraph),
                    weights = NULL, modularity = TRUE, membership = TRUE) #%>% cut_at(no = 23)

#M2.subgraph.communities$degree <- (igraph::degree(M2.subgraph)[M2.subgraph.communities$names])
#M2.subgraph.communities$cluster <- unname(ave(M2.subgraph.communities$degree,
#                                    M2.subgraph.communities$membership,
#                                    FUN=function(x)names(x)[which.max(x)]))
#V(M2.subgraph)$name <- M2.subgraph.communities$cluster
#ln1 <- order(order(M2.subgraph.communities$degree, decreasing=T))

#M2.subgraph.communities <- walktrap.community(M2.subgraph, weights = E(M2.subgraph)$weight)

#_____________________________________________

x <- which.max(sizes(M2.subgraph.communities))
subg1 <- induced.subgraph(M2.subgraph, which(membership(M2.subgraph.communities) == x))  #_________ LARGEST COMPONENT
vcount(subg1)
y <- which.min(sizes(M2.subgraph.communities))
subg2 <- induced.subgraph(M2.subgraph, which(membership(M2.subgraph.communities) == y))  #_________ SMALLEST COMPONENT
vcount(subg2)
#modularity(as.undirected(M2.subgraph), membership = cutat(M2.subgraph.communities, no = 10), #_________ MODULARITY
#           weights = E(M2.subgraph)$weight)

#M2.subgraph.communities <- walktrap.community(M2.subgraph) #__________________________OTHER HIERARCHICAL METHODS
#modularity(M2.subgraph.communities)
#M2.subgraph.communities <- spinglass.community(M2.subgraph)
#modularity(M2.subgraph.communities)
#M2.subgraph.communities <- fastgreedy.community(M2.subgraph)
#modularity(M2.subgraph.communities)
#_ _ _ _ _ _ _ _ _ _ _ _ _ _ _ _ _ _ _ _ _ _ _ _ _ _ _ _ _ _ _ _ _ _ _ _ _ _ _ _ _OR
#sg <- M2.subgraph %>% cluster_spinglass(spins = 10) # produces 10 communities using spinglass algorithm
#walk <- M2.subgraph %>% cluster_walktrap() %>% cut_at(no = 10)
#eb <- M2.subgraph %>% cluster_edge_betweenness() %>% cut_at(no = 10)

M2.subgraph.clustering <- make_clusters(as.undirected(M2.subgraph), membership = M2.subgraph.communities$membership)

#M2.subgraph.comm_den <- as.dendrogram(M2.subgraph.communities) #_________________________________________ DENDROGRAM
#plot_dendrogram(M2.subgraph.comm_den)
#cut_at(M2.subgraph.communities,no = 2)
#for(i in unique(M2.subgraph.communities$membership)) {
#  V(M2.subgraph)[Faction == i]$shape <- M2.subgraph.communities$membership + 1
#set.seed(1234)
#plot(M2.subgraph.communities,M2.subgraph)

V(M2.subgraph)$color <- M2.subgraph.communities$membership + 1
set.seed(1234)
par(mar=c(0,0,0,0))
plot(M2.subgraph.clustering, M2.subgraph, vertex.size = 2, vertex.frame.color = NULL,layout=M2_LGL,asp=FALSE,
vertex.label=NA, edge.curved=TRUE, edge.arrow.size=0.1, vertex.label.dist = 0.5, vertex.label.cex = 0.7, edge.width = 0.5)

set.seed(1234)
par(mar=c(0,0,0,0))
plot(M2.subgraph, vertex.size = 2, vertex.frame.color = NULL,layout=M2_LGL,asp=FALSE,
    vertex.label=NA, edge.curved=TRUE, edge.arrow.size=0.1, vertex.label.dist = 0.5, vertex.label.cex = 0.7, edge.width = 6
```

```
%%R

set.seed(81234)
```

```
#LO1a = layout_nicely(M2.subgraph)                                             #__________ Simplified network plotting
#LO1a = layout.fruchterman.reingold(M2.subgraph)
LO1a = layout_with_lgl(M2.subgraph)
par(mar=c(0,0,0,0))
plot(M2.subgraph,layout=LO1a,vertex.size = 3,asp=FALSE,edge.curved=TRUE, vertex.frame.color = NULL,edge.arrow.size=0.04,
    vertex.color=rainbow(max(M2.subgraph.communities$membership), alpha=0.4)[M2.subgraph.communities$membership], vertex.l

write_graph(M2.subgraph,"/content/drive/My Drive/datasets/M2.subgraph.txt",format = "graphml")

colrs <- adjustcolor( c("gray50", "tomato", "gold", "yellowgreen", "orange", "lightsteelblue2", "dark red"), alpha=.6)

#________________________________________________________________________________________________ Hub scores
                                                                                               #(for directed graphs)

#M2.subgraph.communities$Hub <- igraph::hub.score(M2.subgraph)$vector[M2.subgraph.communities$names]
#M2.subgraph.communities$cluster <- unname(ave(M2.subgraph.communities$Hub,
#                                    M2.subgraph.communities$membership,
#                                    FUN=function(x)names(x)[which.max(x)]))
#V(M2.subgraph)$name <- M2.subgraph.communities$cluster
#ctlGroups = igraph::simplify(contract(M2.subgraph, M2.subgraph.communities$membership,
#                                  vertex.attr.comb = function(x)x[1]),remove.loops=TRUE)
#V(ctlGroups)$hub.score <- unname(igraph::hub.score(ctlGroups)$vector)
##duplicated(V(ctlGroups)$name) | duplicated(V(ctlGroups)$name, fromLast = TRUE)

#set.seed(1234)
#GLOa = layout_with_lgl(ctlGroups)
#par(mar=c(0,0,2,0))
#plot(ctlGroups, layout = GLOa,vertex.size = 7 * sqrt(V(ctlGroups)$hub.score), vertex.color=colrs[1],
#     edge.arrow.size=0.04,asp=FALSE, main="Top degree nodes",
#     edge.curved=TRUE, vertex.frame.color = NULL, vertex.label.cex = 0.8 * (V(ctlGroups)$hub.score),
#     vertex.label = ifelse(unname(igraph::hub.score(ctlGroups)$vector) > 0.85*max(unname(igraph::hub.score(ctlGroups)$vec
#     vertex.label.dist=0,vertex.label.family="Helvetica",vertex.label.color="black")

#___________________________________________________________________________________________ Degree
#M2.subgraph.communities <- edge.betweenness.community(as.undirected(M2.subgraph),
#                    weights = NULL, modularity = TRUE, membership = TRUE) #%>% cut_at(no = 23)
M2.subgraph.communities$degree <- (igraph::degree(M2.subgraph)[M2.subgraph.communities$names])
M2.subgraph.communities$cluster <- unname(ave(M2.subgraph.communities$degree,
                                   M2.subgraph.communities$membership,
                                   FUN=function(x)names(x)[which.max(x)]))
V(M2.subgraph)$name <- M2.subgraph.communities$cluster
ctlGroups = igraph::simplify(contract(M2.subgraph, M2.subgraph.communities$membership,
                                 vertex.attr.comb = function(x)x[1]),remove.loops=TRUE)
V(ctlGroups)$degree <- unname(igraph::degree(ctlGroups))
##duplicated(V(ctlGroups)$name) | duplicated(V(ctlGroups)$name, fromLast = TRUE)

set.seed(81234)
GLOa = layout_with_lgl(ctlGroups)
par(mar=c(0,0,2,0))
plot(ctlGroups, layout = GLOa,vertex.size = sqrt(V(ctlGroups)$degree), vertex.color=colrs[2],
     edge.arrow.size=0.04,asp=FALSE, main="Top Degree nodes",
     edge.curved=TRUE, vertex.frame.color = NULL, vertex.label.cex = 0.03 * (V(ctlGroups)$degree),
     vertex.label = ifelse(unname(igraph::degree(ctlGroups)) > 0.01*max(unname(igraph::degree(ctlGroups))), V(ctlGroups)$r
     vertex.label.dist=0,vertex.label.family="Helvetica",
     vertex.label.color="black")
#__________________________________________________________________________________________ EigenVector
#M2.subgraph.communities <- edge.betweenness.community(as.undirected(M2.subgraph),
#                    weights = NULL, modularity = TRUE, membership = TRUE) #%>% cut_at(no = 23)
M2.subgraph.communities$evcent <- (igraph::evcent(M2.subgraph)$vector[M2.subgraph.communities$names])
M2.subgraph.communities$cluster <- unname(ave(M2.subgraph.communities$evcent,
                                   M2.subgraph.communities$membership,
                                   FUN=function(x)names(x)[which.max(x)]))
V(M2.subgraph)$name <- M2.subgraph.communities$cluster
ctlGroups = igraph::simplify(contract(M2.subgraph, M2.subgraph.communities$membership,
                                 vertex.attr.comb = function(x)x[1]),remove.loops=TRUE)
V(ctlGroups)$evcent <- unname(igraph::evcent(ctlGroups)$vector)
##duplicated(V(ctlGroups)$name) | duplicated(V(ctlGroups)$name, fromLast = TRUE)

set.seed(81234)
GLOa = layout_with_lgl(ctlGroups)
par(mar=c(0,0,2,0))
plot(ctlGroups, layout = GLOa,vertex.size = 7 * sqrt(V(ctlGroups)$evcent), vertex.color=colrs[3],
     edge.arrow.size=0.04,asp=FALSE, main="Top Eigenvector nodes",
     edge.curved=TRUE, vertex.frame.color = NULL, vertex.label.cex = 0.8 * (V(ctlGroups)$evcent),
     vertex.label = ifelse(unname(igraph::evcent(ctlGroups)$vector) > 0.85*max(unname(igraph::evcent(ctlGroups)$vector)),
     vertex.label.dist=0,vertex.label.family="Helvetica",
     vertex.label.color="black")
```

```
#________________________________________________________________________________________________ Closeness
#M2.subgraph.communities <- edge.betweenness.community(as.undirected(M2.subgraph),
#                    weights = NULL, modularity = TRUE, membership = TRUE) #%>% cut_at(no = 23)
M2.subgraph.communities$closeness <- (igraph::closeness(M2.subgraph)[M2.subgraph.communities$names])
M2.subgraph.communities$cluster <- unname(ave(M2.subgraph.communities$closeness,
                                    M2.subgraph.communities$membership,
                                    FUN=function(x)names(x)[which.max(x)]))
V(M2.subgraph)$name <- M2.subgraph.communities$cluster
ctlGroups = igraph::simplify(contract(M2.subgraph, M2.subgraph.communities$membership,
                                  vertex.attr.comb = function(x)x[1]),remove.loops=TRUE)
V(ctlGroups)$closeness <- unname(igraph::closeness(ctlGroups))
##duplicated(V(ctlGroups)$name) | duplicated(V(ctlGroups)$name, fromLast = TRUE)

set.seed(81234)
GLOa = layout_with_lgl(ctlGroups)
par(mar=c(0,0,2,0))
plot(ctlGroups, layout = GLOa,vertex.size = 1500 * V(ctlGroups)$closeness, vertex.color=colrs[4],
     edge.arrow.size=0.04,asp=FALSE, main="Top Closeness nodes",
     edge.curved=TRUE, vertex.frame.color = NULL, vertex.label.cex = 150 * V(ctlGroups)$closeness,
     vertex.label = ifelse(unname(igraph::closeness(ctlGroups)) > 0.8*max(unname(igraph::closeness(ctlGroups))), V(ctlGrou
     vertex.label.dist=0,vertex.label.family="Helvetica",
     vertex.label.color="black")
#___________________________________________________________________________________________________ Betweenness
#M2.subgraph.communities <- edge.betweenness.community(as.undirected(M2.subgraph),
#                    weights = NULL, modularity = TRUE, membership = TRUE) #%>% cut_at(no = 23)
M2.subgraph.communities$betweenness <- (igraph::betweenness(M2.subgraph)[M2.subgraph.communities$names])
M2.subgraph.communities$cluster <- unname(ave(M2.subgraph.communities$betweenness,
                                    M2.subgraph.communities$membership,
                                    FUN=function(x)names(x)[which.max(x)]))
V(M2.subgraph)$name <- M2.subgraph.communities$cluster
ctlGroups = igraph::simplify(contract(M2.subgraph, M2.subgraph.communities$membership,
                                  vertex.attr.comb = function(x)x[1]),remove.loops=TRUE)
V(ctlGroups)$betweenness <- unname(igraph::betweenness(ctlGroups))
##duplicated(V(ctlGroups)$name) | duplicated(V(ctlGroups)$name, fromLast = TRUE)

set.seed(81234)
GLOa = layout_with_lgl(ctlGroups)
par(mar=c(0,0,2,0))
plot(ctlGroups, layout = GLOa,vertex.size = 0.1 * sqrt(V(ctlGroups)$betweenness), vertex.color=colrs[6],
     edge.arrow.size=0.04,asp=FALSE, main="Top Betweenness nodes",
     edge.curved=TRUE, vertex.frame.color = NULL, vertex.label.cex = 0.00045 * (V(ctlGroups)$betweenness),
     vertex.label = ifelse(unname(igraph::betweenness(ctlGroups)) > 0.7*max(unname(igraph::betweenness(ctlGroups))), V(ctl
     vertex.label.dist=0,vertex.label.family="Helvetica",
     vertex.label.color="black")
#_____________________________________________________________________________________________________ Authority
##M2.subgraph.communities <- edge.betweenness.community(as.undirected(M2.subgraph),                 #(for directed graphs)
##                    weights = NULL, modularity = TRUE, membership = TRUE) #%>% cut_at(no = 23)
#M2.subgraph.communities$Authority <- igraph::authority.score(M2.subgraph)$vector[M2.subgraph.communities$names]
#M2.subgraph.communities$cluster <- unname(ave(M2.subgraph.communities$Authority,
#                                    M2.subgraph.communities$membership,
#                                    FUN=function(x)names(x)[which.max(x)]))
#V(M2.subgraph)$name <- M2.subgraph.communities$cluster
#ctlGroups = igraph::simplify(contract(M2.subgraph, M2.subgraph.communities$membership,
#                                  vertex.attr.comb = function(x)x[1]),remove.loops=TRUE)
#V(ctlGroups)$authority.score <- unname(igraph::authority.score(ctlGroups)$vector)
##duplicated(V(ctlGroups)$name) | duplicated(V(ctlGroups)$name, fromLast = TRUE)

#set.seed(1234)
#GLOa = layout_with_lgl(ctlGroups)
#par(mar=c(0,0,0,0))
#plot(ctlGroups, layout = GLOa,vertex.size = 7 * sqrt(V(ctlGroups)$authority.score), vertex.color=colrs[7],
#     edge.arrow.size=0.04,asp=FALSE, main="Community number with the highest degree node",
#     edge.curved=TRUE, vertex.frame.color = NULL, vertex.label.cex = 0.8 * (V(ctlGroups)$authority.score),
#     vertex.label = ifelse(unname(igraph::authority.score(ctlGroups)$vector) > 0.85*max(unname(igraph::authority.score(ct
#     vertex.label.dist=0,vertex.label.family="Helvetica",
#     vertex.label.color="black")

#__________________________________________________________________________________________ Community numbers on graph

M2.subgraph.communities <- edge.betweenness.community(as.undirected(M2.subgraph),
                   weights = NULL, modularity = TRUE, membership = TRUE) #%>% cut_at(no = 23)
M2.subgraph.communities$Hub <- igraph::hub.score(M2.subgraph)$vector[M2.subgraph.communities$names]
M2.subgraph.communities$cluster <- unname(ave(M2.subgraph.communities$Hub,
                                    M2.subgraph.communities$membership,
                                    FUN=function(x)names(x)[which.max(x)]))
V(M2.subgraph)$name <- M2.subgraph.communities$membership[which(M2.subgraph.communities$names == M2.subgraph.communities$c
ctlGroups = igraph::simplify(contract(M2.subgraph, M2.subgraph.communities$membership,
```

```
                                  vertex.attr.comb = function(x)x[1]),remove.loops=TRUE)
V(ctlGroups)$hub.score <- unname(igraph::hub.score(ctlGroups)$vector)

set.seed(81234)
GLOa = layout_with_lgl(ctlGroups)
par(mar=c(0,0,0,0))
plot(ctlGroups, layout = GLOa,vertex.size = 5,
     edge.arrow.size=0.04,asp=FALSE, main="Community numbers",
     edge.curved=TRUE, vertex.frame.color = NULL, vertex.label.cex = 1.2,vertex.color=colrs[5],
     vertex.label = ifelse(unname(igraph::hub.score(ctlGroups)$vector) >= 0, V(ctlGroups)$name, NA),
     vertex.label.dist=0.5,vertex.label.family="Helvetica",vertex.label.color="black")

M2.subgraph <- read_graph("/content/drive/My Drive/datasets/M2.subgraph.txt",format = "graphml")
subg1 <- induced.subgraph(M2.subgraph, which(membership(M2.subgraph.communities) == 67))
V(subg1)$name
#_______________________________________________________________________________
Rada = min(dist(GLOa))
LO2a = matrix(0, nrow=vcount(M2.subgraph), ncol=2)
for(i in unique(M2.subgraph.communities$membership)) {
    #vids <- M2.subgraph.communities$membership[[i]]
    #Clusta = induced_subgraph(M2.subgraph, vids)
    Clusta = induced_subgraph(M2.subgraph, which(M2.subgraph.communities$membership == i))
    CLOa = layout_with_fr(Clusta)
    LO2a[which(M2.subgraph.communities$membership == i), ] = t(t(scale(CLOa)*Rada/3) + GLOa[i,])
      }

set.seed(81234)
par(mar=c(0,0,0,0))
plot(M2.subgraph, layout=LO2a, vertex.label=NA, vertex.size = 3, edge.arrow.size=0.04, asp=FALSE,edge.curved=TRUE, vertex.
    vertex.color=rainbow(max(M2.subgraph.communities$membership), alpha=0.4)[M2.subgraph.communities$membership])

```

```
%%R -o commSummary2

#install.packages("centiserve")
#library(centiserve)

#arpack_defaults$maxiter = 10000

#Hub <- igraph::hub.score(M2.subgraph, options=arpack_defaults)$vector
#Authority <- igraph::authority.score(M2.subgraph)
Closeness <- igraph::closeness(M2.subgraph)
Betweenness <- igraph::betweenness(M2.subgraph)
#centr_lap_top <- centiserve::laplacian(M2.subgraph)
Degree <- igraph::degree(M2.subgraph)
EigVec <- igraph::evcent(M2.subgraph)$vector

commSummary <- data.frame(
  M2.subgraph.communities$names,
  M2.subgraph.communities$membership,
  #M2.subgraph.communities$modularity,
  #centr_lap_top,
  #Hub,
  #Authority,
  Closeness,
  Betweenness,
  Degree,
  EigVec
)

colnames(commSummary) <- c("Gene", "Community", "Closeness", "Betweenness", "Degree", "EigVec")
options(scipen=999)

#save(commSummary, file="/content/drive/My Drive/datasets/commSummary.Rdata")

#_______________________________________________________________________________

#load("/content/drive/My Drive/datasets/commSummary.Rdata")

cols1 <- grep("Closeness", names(commSummary))
#commSummary2 <- commSummary[rowSums(commSummary[cols1] > 1.3*mean(centr_lap_top)) == length(cols1), ]
#commSummary2 <- commSummary[rowSums(commSummary['Hub_Score'] > 1.5*mean(Hub))== length(cols1), ]
commSummary2 <- commSummary[rowSums(commSummary['Closeness'] > min(Closeness))== length(cols1), ]
#commSummary2 <- commSummary[rowSums(commSummary[cols1] >= min(centr_lap_top)) == length(cols1), ]

```

```
head(commSummary2)

```

```
import numpy as np
import pandas as pd

Gene_Lap = commSummary2.groupby('Community').count().sort_values(by=['Gene'], ascending=False)
comm_to_R=[]

#for i in range(len(list(Gene_Lap.index)[0:4])):     #_____ Top 5 communities
for i in range(len(list(Gene_Lap.index))):           #_____ All communities
  comm_to_R.append(list(Gene_Lap.index)[i])

back_genes = pd.read_csv('/content/drive/My Drive/datasets/back_genes.csv')
background_uni = back_genes['x']
background_uni.dropna(how='all', inplace=True)

```

```
Start coding or generate with AI.
```

```
Start coding or generate with AI.
```

```
Start coding or generate with AI.
```

```
%%R -i comm_to_R -o commSum2_subg_to

inp_ab = 1.3
commSum2_subg_to <- list()
con_fin <- list()

for(i in 1:length(comm_to_R)) {
subg <- paste("subg", i, sep = "")
subg_to<-assign(subg, induced.subgraph(M2.subgraph, which(membership(M2.subgraph.communities) == unlist(comm_to_R)[i])))

#subg3_lap_top <- centiserve::laplacian(subg_to)
#subg3_hub_top <- igraph::hub.score(subg_to, options=arpack_defaults)$vector
subg3_clos_top <- igraph::closeness(subg_to)
subg3_betw_top <- igraph::betweenness(subg_to)
subg3_deg_top <- igraph::degree(subg_to)
subg3_eig_top <- igraph::evcent(subg_to)$vector

commSum_subg3 <- data.frame(
  V(subg_to)$name,
  subg3_clos_top,
  subg3_betw_top,
  subg3_deg_top,
  subg3_eig_top
  )

colnames(commSum_subg3) <- c("Gene", "Closeness", "Betweenness", "Degree", "EigenVector")

options(scipen=999)

cols3 <- grep("Closeness", names(commSum_subg3))

commSum2_subg <- paste("commSum2_subg", i, sep = "")
commSum2_subg_to[i] <- list(assign(commSum2_subg, commSum_subg3[rowSums(commSum_subg3[cols3]  >= min(subg3_clos_top)) == le
}

```

```
'''
import numpy
import numpy as np
import pandas as pd

con_fin1 = []
fin_tmpa = []
fin_tmpb = []
fin_tmpc = []
fin_tmpd = []

for i in range(len(commSum2_subg_to)) :
  con_fin = []
```

```
  commSum2_subg_tmp1 = pd.DataFrame(commSum2_subg_to[i])

  commSum2_subg_tmp2a = pd.DataFrame(np.transpose(commSum2_subg_tmp1.values[1,:]))
  commSum2_subg_tmp2b = pd.DataFrame(np.transpose(commSum2_subg_tmp1.values[2,:]))
  commSum2_subg_tmp2c = pd.DataFrame(np.transpose(commSum2_subg_tmp1.values[3,:]))
  commSum2_subg_tmp2d = pd.DataFrame(np.transpose(commSum2_subg_tmp1.values[4,:]))

  commSum2_subg_tmp2a.columns = ['Closeness']
  commSum2_subg_tmp2b.columns = ['Betweenness']
  commSum2_subg_tmp2c.columns = ['Degree']
  commSum2_subg_tmp2d.columns = ['EigenVector']

  commSum2_subg_tmp1 = pd.DataFrame(np.transpose(commSum2_subg_tmp1.values[0,:]))
  commSum2_subg_tmp1.columns = ['Gene']

  commSum2_subg_tmp = pd.concat([commSum2_subg_tmp1.reset_index(drop=True),
                                 commSum2_subg_tmp2a, commSum2_subg_tmp2b,
                                 commSum2_subg_tmp2c, commSum2_subg_tmp2d], axis=1)

  commSum2_subg_tmp['Closeness'].astype(float).nlargest(int(commSum2_subg_tmp.shape[0]*0.3)).reset_index()
  fin_tmpa.append(commSum2_subg_tmp['Closeness'].astype(float).nlargest(int(commSum2_subg_tmp.shape[0]*0.3)).reset_index())
  commSum2_subg_tmp['Betweenness'].astype(float).nlargest(int(commSum2_subg_tmp.shape[0]*0.3)).reset_index()
  fin_tmpb.append(commSum2_subg_tmp['Betweenness'].astype(float).nlargest(int(commSum2_subg_tmp.shape[0]*0.3)).reset_index(
  commSum2_subg_tmp['Degree'].astype(float).nlargest(int(commSum2_subg_tmp.shape[0]*0.3)).reset_index()
  fin_tmpc.append(commSum2_subg_tmp['Degree'].astype(float).nlargest(int(commSum2_subg_tmp.shape[0]*0.3)).reset_index())
  commSum2_subg_tmp['EigenVector'].astype(float).nlargest(int(commSum2_subg_tmp.shape[0]*0.3)).reset_index()
  fin_tmpd.append(commSum2_subg_tmp['EigenVector'].astype(float).nlargest(int(commSum2_subg_tmp.shape[0]*0.3)).reset_index(

  con_fin.append(commSum2_subg_tmp1.values[fin_tmpa[i]['index']])
  con_fin.append(commSum2_subg_tmp1.values[fin_tmpb[i]['index']])
  con_fin.append(commSum2_subg_tmp1.values[fin_tmpc[i]['index']])
  con_fin.append(commSum2_subg_tmp1.values[fin_tmpd[i]['index']])
  flat_con_fin = [item for sublist in con_fin for item in sublist]
  con_finx = [i[0] for i in flat_con_fin]
  con_fin1.append(list(set(con_finx)))

%R -i con_fin1
#%R save(con_fin1, file="/content/drive/My Drive/datasets/con_fin1.Rdata")

'''

'''
You can choose genes by 'Closeness', 'EigenVector', 'Degree', or 'Betweenness'
or all of them (as in this module of the comment). You can also disjoint the
biggest communities into smaller ones and try the genes from each one of them!!
'''
```

```
'''
%%R -i con_fin1

ensLookup_tmp = list()

for (i in 1:length(con_fin1)) {
ensLookup_tmp[[i]] <- unlist(con_fin1[i])
}
print("top 30% of genes kept:")   # nlargest(int(commSum2_subg_tmp.shape[0]*0.3))

print("Number of communities:")
print(length(ensLookup_tmp))

j=0
for (i in 1:length(ensLookup_tmp)) {
if (length(unlist(con_fin1[i]))>2){
j=j+1
}
}
print("Number of communities with more than 2 genes:")
print(j)

'''

```

```
#______________________________________________________________________________________________________________ Installing li
%%R

if (!requireNamespace("BiocManager", quietly = TRUE))
```

```
    install.packages("BiocManager")
BiocManager::install("biomaRt", force = TRUE)
library(biomaRt)
#require("biomaRt")
mart <- useMart("ENSEMBL_MART_ENSEMBL")
mart <- useDataset("hsapiens_gene_ensembl", mart)

devtools::install_github(c("GuangchuangYu/DOSE", "GuangchuangYu/enrichplot", "GuangchuangYu/GOSemSim", "eliocamp/ggnewscale

BiocManager::install("clusterProfiler")
library(clusterProfiler)

organism = "org.Hs.eg.db"
BiocManager::install(organism, character.only = TRUE)
library(organism, character.only = TRUE)

BiocManager::install("DOSE")
library(DOSE)

#BiocManager::install("ReactomePA")
#library(ReactomePA)

```

```
'''
%%R -i con_fin,background_uni

#____________________________________________________________________________ I. GO (ENTREZ through biomart)
                                                                            #(Plot communities one-by-one, manually)
df = as.data.frame(org.Hs.egGO)
go_gene_list = unique(sort(df$gene_id))

require("biomaRt")
mart <- useMart("ENSEMBL_MART_ENSEMBL")
mart <- useDataset("hsapiens_gene_ensembl", mart)

ensLookup_1 = list()
annotLookup = list()

geneList <- background_uni
geneList <- unlist(geneList)
geneList <- geneList[order(nchar(geneList), geneList)]

df = as.data.frame(org.Hs.egGO)
go_gene_list = unique(sort(df$gene_id))
go_gene_list.df <- bitr(go_gene_list, fromType = "ENTREZID",
        toType = c("ENSEMBL", "SYMBOL"),
        OrgDb = org.Hs.eg.db)

geneList.df <- bitr(as.character(unique(sort(as.character(geneList)))), fromType = "ENTREZID",
        toType = c("ENSEMBL", "SYMBOL"),
        OrgDb = org.Hs.eg.db)

dfk = as.data.frame(org.Hs.egPATH)
kegg_gene_list = unique(sort(dfk$gene_id))

xp = list()
ego1 = list()
ego2 = list()
ego3 = list()
x = list()
kk = list()
gg01 = list()
gg02 = list()
gene.df = list()

#if (file.exists(fn)) {
#  file.remove(fn)
#}

for (i in 1:length(con_fin1)) {
ensLookup_1[[i]] <- unlist(con_fin1[i])

if (length(unlist(con_fin1[i]))>2){
annotLookup[[i]] <- getBM(
  mart=mart,
  attributes=c("ensembl_transcript_id", "ensembl_gene_id",
```

```
    "gene_biotype", "external_gene_name", "entrezgene_id"),
  filter="ensembl_gene_id",
  values=ensLookup_1[[i]],
  uniqueRows=TRUE)
annotLookup[[i]] <- data.frame(ensLookup_1[[i]][match(annotLookup[[i]]$ensembl_gene_id, ensLookup_1[[i]])],
  annotLookup[[i]])
annotLookup[[i]] <- subset(annotLookup[[i]], (!is.na(annotLookup[[i]]['entrezgene_id'])))
annotLookup[[i]] <-annotLookup[[i]][annotLookup[[i]]['gene_biotype'] == "protein_coding", ]
}
#else {annotLookup[[i]] == 0
#      }
#colnames(annotLookup[[i]]) <- c(
#  "original_id",
#  c("ensembl_transcript_id", "ensembl_gene_id",
#    "gene_biotype", "external_gene_name", "EntrezID"))

#________________________________________________________________________________ Using bitr for translating between "ENSEM
}

for(i in 1:length(annotLookup)) {

print("___________________________Ia. enrichGO (BP) _______________________________________________")
l1 <- enrichGO(gene         = unique(sort(annotLookup[[i]]$ensembl_gene_id)),
                 universe      = go_gene_list.df$ENSEMBL,                       #org.Hs.egGO
                 #universe      = geneList.df$ENSEMBL,                         #background genes
                 OrgDb         = org.Hs.eg.db,
                #keyType       = "ENTREZID",
                 keyType       = "ENSEMBL",
                 ont           = "BP",
                 pAdjustMethod = "BH",                                          #pAdjustMethod = "fdr"
                 pvalueCutoff  = 0.05,
                 qvalueCutoff  = 0.1,
                 readable      = TRUE)
if (length(l1$ID) == 0) {
ego1[[i]] <- "NULL"
}
else{
ego1[[i]] <- data.frame(
 i,
 l1$ID,
 l1$Description,
 l1$GeneRatio,
 l1$BgRatio,
 l1$pvalue,
 l1$p.adjust,
 l1$qvalue,
 l1$geneID,
 l1$Count
)
colnames(ego1[[i]]) <- c("Community#", "ID", "Description", "GeneRatio",
                           "BgRatio", "pvalue", "p.adjust", "qvalue",
                           "geneID","Count")
options(scipen=999)
}

print("___________________________Ib. enrichGO (CC) _______________________________________________")
l2 <- enrichGO(gene         = unique(sort(annotLookup[[i]]$ensembl_gene_id)),
                 universe      = go_gene_list.df$ENSEMBL,                       #org.Hs.egGO
                 #universe      = geneList.df$ENSEMBL,                         #background genes
                 OrgDb         = org.Hs.eg.db,
                #keyType       = "ENTREZID",
                 keyType       = "ENSEMBL",
                 ont           = "CC",
                 pAdjustMethod = "BH",                                          #pAdjustMethod = "fdr"
                 pvalueCutoff  = 0.05,
                 qvalueCutoff  = 0.1,
                 readable      = TRUE)
if (length(l2) == 0){
ego2[[i]] <- "NULL"
}
else{
ego2[[i]] <- l2
}
#head(summary(as.data.frame(ego2[[i]])))
#print(ego2[[i]])
cat('\n')
print("___________________________Ic. enrichGO (MF) _______________________________________________")
```

```
l3 <- enrichGO(gene         = unique(sort(annotLookup[[i]]$ensembl_gene_id)),
                 universe      = go_gene_list.df$ENSEMBL,                       #org.Hs.egGO
                 #universe      = geneList.df$ENSEMBL,                         #background genes
                 OrgDb         = org.Hs.eg.db,
                #keyType       = "ENTREZID",
                 keyType       = "ENSEMBL",
                 ont           = "MF",
                 pAdjustMethod = "BH",                                          #pAdjustMethod = "fdr"
                 pvalueCutoff  = 0.05,
                 qvalueCutoff  = 0.1,
                 readable      = TRUE)
if (length(l3) == 0){
ego3[[i]] <- "NULL"
}
else{
ego3[[i]] <- l3
}
#head(summary(as.data.frame(ego3[[i]])))
#print(ego3[[i]])
cat('\n')
print("___________________________II. enrichDO_______________________________________________")
l4 <- enrichDO(gene         = as.character(unique(sort(annotLookup[[i]]$entrezgene_id))),
              ont           = "DO",
              pvalueCutoff  = 0.05,
              pAdjustMethod = "BH",
              universe      = go_gene_list,                                     #org.Hs.egGO
              #universe      = geneList.df$ENTREZID,                            #background genes
              minGSSize     = 10,
              maxGSSize     = 1000,
              qvalueCutoff  = 0.1,
              readable      = TRUE)
if (length(l4$ID) == 0) {
x[[i]] <- "NULL"
}
else{
x[[i]] <- data.frame(
 i,
 l4$ID,
 l4$Description,
 l4$GeneRatio,
 l4$BgRatio,
 l4$pvalue,
 l4$p.adjust,
 l4$qvalue,
 l4$geneID,
 l4$Count
)
colnames(x[[i]]) <- c("Community#", "ID", "Description", "GeneRatio",
                           "BgRatio", "pvalue", "p.adjust", "qvalue",
                           "geneID","Count")
options(scipen=999)
}

print("___________________________III. enrichKEGG_______________________________________________")
l5 <- enrichKEGG(gene         = as.character(unique(sort(annotLookup[[i]]$entrezgene_id))),
                 organism     = 'hsa',
                 pvalueCutoff = 0.05,
                 universe  = kegg_gene_list,                                    #org.Hs.egPATH
                 #universe = geneList.df$ENTREZID,                              #background genes
                 qvalueCutoff  = 0.1)
if (length(l5$ID) == 0) {
kk[[i]] <- "NULL"
}
else{
kk[[i]] <- data.frame(
 i,
 l5$ID,
 l5$Description,
 l5$GeneRatio,
 l5$BgRatio,
 l5$pvalue,
 l5$p.adjust,
 l5$qvalue,
 l5$geneID,
 l5$Count
)
colnames(kk[[i]]) <- c("Community#", "ID", "Description", "GeneRatio",
```

```
                            "BgRatio", "pvalue", "p.adjust", "qvalue",
                            "geneID","Count")
options(scipen=999)
}
cat('\n')
cat('\n')
cat('\n')
cat('\n')
cat('\n')
print("########################################################################################################")
}
'''
```

```
'''
#_______________________________________________________________________________ Print all of the above results to Tables
%R -o kk,x,ego1


for i in range(len(ego1)):
  if (len(ego1[i])!=1):
    df1 = pd.DataFrame(ego1[i]).T
    df1.rename(columns={0: 'Community#', 1: 'ID',
          2: 'Description', 3: 'GeneRatio',
          4: 'BgRatio', 5: 'pvalue',
          6: 'p.adjust', 7: 'qvalue',
          8: 'geneID', 9: 'Count'},inplace=True)
    print(df1.to_string())
    print("################################################################################################################
print("__________________________________________________________________________________________________________________")
print("__________________________________________________________________________________________________________________")
print("__________________________________________________________________________________________________________________")


for i in range(len(x)):
  if (len(x[i])!=1):
    df4 = pd.DataFrame(x[i]).T
    df4.rename(columns={0: 'Community#', 1: 'ID',
          2: 'Description', 3: 'GeneRatio',
          4: 'BgRatio', 5: 'pvalue',
          6: 'p.adjust', 7: 'qvalue',
          8: 'geneID', 9: 'Count'},inplace=True)
    print(df4.to_string())
    print("################################################################################################################
print("__________________________________________________________________________________________________________________")
print("__________________________________________________________________________________________________________________")
print("__________________________________________________________________________________________________________________")


for i in range(len(kk)):
  if (len(kk[i])!=1):
    df5 = pd.DataFrame(kk[i]).T
    df5.rename(columns={0: 'Community#', 1: 'ID',
          2: 'Description', 3: 'GeneRatio',
          4: 'BgRatio', 5: 'pvalue',
          6: 'p.adjust', 7: 'qvalue',
          8: 'geneID', 9: 'Count'},inplace=True)
    print(df5.to_string())
    print("################################################################################################################
'''
```

```

fin_tmpar = []
fin_tmpbr = []
fin_tmpcr = []
fin_tmpdr = []
con_fin1t = []

for i in range(18) :
  con_fin = []
  commSum2_subg_tmp1 = pd.DataFrame(commSum2_subg_to[i])

  commSum2_subg_tmp2a = pd.DataFrame(np.transpose(commSum2_subg_tmp1.values[1,:]))
  commSum2_subg_tmp2b = pd.DataFrame(np.transpose(commSum2_subg_tmp1.values[2,:]))
  commSum2_subg_tmp2c = pd.DataFrame(np.transpose(commSum2_subg_tmp1.values[3,:]))
  commSum2_subg_tmp2d = pd.DataFrame(np.transpose(commSum2_subg_tmp1.values[4,:]))

```

```
commSum2_subg_tmp2a.columns = ['Closeness']
commSum2_subg_tmp2b.columns = ['Betweenness']
commSum2_subg_tmp2c.columns = ['Degree']
commSum2_subg_tmp2d.columns = ['EigenVector']

commSum2_subg_tmp1 = pd.DataFrame(np.transpose(commSum2_subg_tmp1.values[0,:]))
commSum2_subg_tmp1.columns = ['Gene']

commSum2_subg_tmp = pd.concat([commSum2_subg_tmp1.reset_index(drop=True),
                               commSum2_subg_tmp2a, commSum2_subg_tmp2b,
                               commSum2_subg_tmp2c, commSum2_subg_tmp2d], axis=1)

fin_tmpar.append(commSum2_subg_tmp['Closeness'].astype(float).nlargest(int(commSum2_subg_tmp.shape[0]*1)).reset_index())
fin_tmpbr.append(commSum2_subg_tmp['Betweenness'].astype(float).nlargest(int(commSum2_subg_tmp.shape[0]*1)).reset_index()
fin_tmpcr.append(commSum2_subg_tmp['Degree'].astype(float).nlargest(int(commSum2_subg_tmp.shape[0]*1)).reset_index())
fin_tmpdr.append(commSum2_subg_tmp['EigenVector'].astype(float).nlargest(int(commSum2_subg_tmp.shape[0]*1)).reset_index()


con_fin.append(commSum2_subg_tmp1.values[fin_tmpar[i]['index']])
con_fin.append(commSum2_subg_tmp1.values[fin_tmpbr[i]['index']])
con_fin.append(commSum2_subg_tmp1.values[fin_tmpcr[i]['index']])
con_fin.append(commSum2_subg_tmp1.values[fin_tmpdr[i]['index']])
flat_con_fin = [item for sublist in con_fin for item in sublist]
con_finx = [i[0] for i in flat_con_fin]
con_fin1t.append(list(set(con_finx)))
print(len(con_fin1t[i]))
%R -i con_fin1t

```

```
%%R                                   #__________________________________________ I. EnrichGO

ego1r = list()
annotLookup = list()
ensLookup_tmpr = list()

df = as.data.frame(org.Hs.egGO)
go_gene_list = unique(sort(df$gene_id))
go_gene_list.df <- bitr(go_gene_list, fromType = "ENTREZID",
        toType = c("ENSEMBL", "SYMBOL"),
        OrgDb = org.Hs.eg.db)

for (i in 1:18) {
ensLookup_tmpr[[i]] <- unlist(con_fin1t[i])
#print(head(ensLookup_tmpr[[i]]))

if (length(unlist(con_fin1t[i]))>2){
annotLookup[[i]] <- getBM(
  mart=mart,
  attributes=c("ensembl_transcript_id", "ensembl_gene_id",
    "gene_biotype", "external_gene_name", "entrezgene_id"),
  filter="ensembl_gene_id",
  values=ensLookup_tmpr[[i]],
  uniqueRows=TRUE)
annotLookup[[i]] <- data.frame(ensLookup_tmpr[[i]][match(annotLookup[[i]]$ensembl_gene_id, ensLookup_tmpr[[i]])],
  annotLookup[[i]])
annotLookup[[i]] <- subset(annotLookup[[i]], (!is.na(annotLookup[[i]]['entrezgene_id'])))
annotLookup[[i]] <-annotLookup[[i]][annotLookup[[i]]['gene_biotype'] == "protein_coding", ]
}

print("__________________________Ia. enrichGO (BP) ______________________________________________")
l1 <- enrichGO(gene         = unique(sort(ensLookup_tmpr[[i]])),
                 universe      = go_gene_list.df$ENSEMBL,                      #org.Hs.egGO
                 #universe      = geneList.df$ENSEMBL,                         #background genes
                 OrgDb         = org.Hs.eg.db,
                #keyType       = "ENTREZID",
                 keyType       = "ENSEMBL",
                 ont           = "BP",
                 pAdjustMethod = "BH",                                         #pAdjustMethod = "fdr"
                 pvalueCutoff  = 0.05,
                 qvalueCutoff  = 0.1,
                 readable      = TRUE)
if (length(l1$ID) == 0) {
ego1r[[i]] <- "NULL"
}
else{
ego1r[[i]] <- data.frame(
```

```
 i,
 l1$ID,
 l1$Description,
 l1$GeneRatio,
 l1$BgRatio,
 l1$pvalue,
 l1$p.adjust,
 l1$qvalue,
 l1$geneID,
 l1$Count
)
colnames(ego1r[[i]]) <- c("Community#", "ID", "Description", "GeneRatio",
                           "BgRatio", "pvalue", "p.adjust", "qvalue",
                           "geneID","Count")
options(scipen=999)
}
}
```

```
%R -o ego1r

for i in range(len(ego1r)):
  if (len(ego1r[i])!=1):
    df1 = pd.DataFrame(ego1r[i]).T
    df1.rename(columns={0: 'Community#', 1: 'ID',
          2: 'Description', 3: 'GeneRatio',
          4: 'BgRatio', 5: 'pvalue',
          6: 'p.adjust', 7: 'qvalue',
          8: 'geneID', 9: 'Count'},inplace=True)
    print(df1.to_string())
    print("################################################################################################################

```

```
%%R                                  #_________________________________________ II. KEGG

dfk = as.data.frame(org.Hs.egPATH)
kegg_gene_list = unique(sort(dfk$gene_id))

ensLookup_tmpr = list()
kk = list()

for (i in 1:18)
{
ensLookup_tmpr[[i]] <- unlist(con_fin1t[i])
#print(head(ensLookup_tmpr[[i]]))

if (length(unlist(con_fin1t[i]))>2){
annotLookup[[i]] <- getBM(
  mart=mart,
  attributes=c("ensembl_transcript_id", "ensembl_gene_id",
    "gene_biotype", "external_gene_name", "entrezgene_id"),
  filter="ensembl_gene_id",
  values=ensLookup_tmpr[[i]],
  uniqueRows=TRUE)
annotLookup[[i]] <- data.frame(ensLookup_tmpr[[i]][match(annotLookup[[i]]$ensembl_gene_id, ensLookup_tmpr[[i]])],
  annotLookup[[i]])
annotLookup[[i]] <- subset(annotLookup[[i]], (!is.na(annotLookup[[i]]['entrezgene_id'])))
annotLookup[[i]] <-annotLookup[[i]][annotLookup[[i]]['gene_biotype'] == "protein_coding", ]
}

print("___________________________III. enrichKEGG_______________________________________________")
l5 <- enrichKEGG(gene         = as.character(unique(sort(annotLookup[[i]]$entrezgene_id))),
                 organism     = 'hsa',
                 pvalueCutoff = 0.05,
                 universe  = kegg_gene_list,                                 #org.Hs.egPATH
                 #universe = geneList.df$ENTREZID,                           #background genes
                 qvalueCutoff  = 0.1)
if (length(l5$ID) == 0) {
kk[[i]] <- "NULL"
}
else{
kk[[i]] <- data.frame(
 i,
 l5$ID,
 l5$Description,
 l5$GeneRatio,
 l5$BgRatio,
```

```
 l5$pvalue,
 l5$p.adjust,
 l5$qvalue,
 l5$geneID,
 l5$Count
)
colnames(kk[[i]]) <- c("Community#", "ID", "Description", "GeneRatio",
                           "BgRatio", "pvalue", "p.adjust", "qvalue",
                           "geneID","Count")
options(scipen=999)
}
print("#########################################################################################################")
}

```

```
%R -o kk

for i in range(len(kk)):
  if (len(kk[i])!=1):
    df5 = pd.DataFrame(kk[i]).T
    df5.rename(columns={0: 'Community#', 1: 'ID',
          2: 'Description', 3: 'GeneRatio',
          4: 'BgRatio', 5: 'pvalue',
          6: 'p.adjust', 7: 'qvalue',
          8: 'geneID', 9: 'Count'},inplace=True)
    print(df5.to_string())
    print("###############################################################################################################

```

```
%%R                          #__________________________________________________ Community numbers and how many genes they

lop1 = list()
lop2 = list()

for(i in 1:length(commSum2_subg_to)) {
subg1 <- induced.subgraph(M2.subgraph, which(membership(M2.subgraph.communities) == i))
#print(length(V(subg1)$name))
lop1[i] <- length(V(subg1)$name)
lop2[i] <- i
}

gene_number = c(unlist(lop1))
community_number = c(unlist(lop2))
df = data.frame(gene_number, community_number)
head(df[order(df$gene_number, decreasing = TRUE),],100 )

```

```
%%R

#_____________________________________________________________________________________
#for(i in 1:10) {
#subg1 <- induced.subgraph(M2.subgraph, which(membership(M2.subgraph.communities) == i))
#print(length(V(subg1)$name))
#}

     #number of genes     #community
#[1]    741                 1
#[1]    6                   2
#[1]    1360                3
#[1]    364                 4
#[1]    307                 5
#[1]    4                   6
#[1]    2                   7
#[1]    462                 8
#[1]    143                 9
#[1]    2                   10

#_______________________________________________
#for i in range(10) :
#  print(pd.DataFrame(commSum2_subg_to[i]).shape)


#number of genes      #community
#(5, 1360)              3
#(5, 741)               1
```

```
#(5, 462)               8
#(5, 364)               4
#(5, 307)               5
#(5, 143)               9
#(5, 32)
#(5, 28)
#(5, 12)
#(5, 10)

#__________________________________________
#ensLookup_tmpr = list()
#for (i in 1:10) {
#ensLookup_tmpr[[i]] <- unlist(con_fin1t[i])
#print(length(ensLookup_tmpr[[i]]))
#}

#keep genes
#from each community:    all     top 30%   community    Table column
#                        ___________________________________________
#[1]                     1360      955         3            1
#[1]                     741       519         1            2
#[1]                     462       324         8            3
#[1]                     364       242         4            4
#[1]                     307       216         5            5
#[1]                     143       92          9            6
#[1]                     32        19                       7
#[1]                     28        13                       8
#[1]                     12        4                        9
#[1]                     10        4

```
