# Supplementary material for: Exploration of schizophrenia-associated gene modules using graph theory, co-expression networks, and dimensionality reduction
Source: PLoS One. 2026 Apr 15;21(4):e0346663. doi: 10.1371/journal.pone.0346663 (PMC13082716; doi:10.1371/journal.pone.0346663)
Supplement: S1 File — (PDF) [file pone.0346663.s009.pdf]

Table 1. Several significant module eigengene-trait associations (correlations and their corresponding p-values) are being displayed, with the most important of them being the first column (diagnosis). Negative correlations are color-coded by green shades and positive correlations by red shades.

Module-Trait relationships

MEthistle	−0.042(0.4)	−0.091(0.08)	0.04(0.4)	−0.074(0.2)	0.024(0.6)
MEsaddlebrown	−0.043(0.4)	0.016(0.8)	0.043(0.4)	0.033(0.5)	−0.013(0.8)
MEcoral3	−0.032(0.5)	0.012(0.8)	0.055(0.3)	0.028(0.6)	0.029(0.6)
MElightcoral	0.062(0.2)	0.017(0.7)	0.035(0.5)	0.0075(0.9)	0.023(0.7)
MEantiquewhite2	0.052(0.3)	0.024(0.6)	0.044(0.4)	−0.0029(1)	0.0077(0.9)
MEmagenta4	−0.035(0.5)	0.032(0.5)	−0.053(0.3)	−0.018(0.7)	0.038(0.5)
MEblue4	−0.042(0.4)	0.026(0.6)	−0.061(0.2)	−0.041(0.4)	0.051(0.3)
MEskyblue2	−0.042(0.4)	0.02(0.7)	0.041(0.4)	−0.095(0.06)	0.044(0.4)
MEdarkturquoise	0.07(0.2)	−0.088(0.08)	0.047(0.4)	0.00062(1)	0.012(0.8)
MEdarkorange	−0.043(0.4)	0.029(0.6)	−0.058(0.3)	0.047(0.4)	−0.0071(0.9)
MEwhite	−0.035(0.5)	−0.095(0.06)	0.043(0.4)	−0.025(0.6)	0.13(0.01)
MEmediumpurple4	−0.034(0.5)	0.015(0.8)	−0.068(0.2)	−0.019(0.7)	0.069(0.2)
MEpalevioletred2	0.067(0.2)	0.022(0.7)	−0.061(0.2)	−0.033(0.5)	0.033(0.5)
MEfloralwhite	−0.037(0.5)	0.021(0.7)	0.048(0.4)	0.013(0.8)	−0.022(0.7)
MEnavajowhite2	0.064(0.2)	0.035(0.5)	−0.052(0.3)	−0.025(0.6)	−0.053(0.3)
MElightpink4	−0.038(0.5)	0.025(0.6)	0.06(0.2)	0.024(0.6)	−0.055(0.3)
MEpalevioletred3	0.074(0.1)	0.024(0.6)	0.048(0.3)	0.022(0.7)	−0.049(0.3)
MEthistle4	0.083(0.1)	0.027(0.6)	0.043(0.4)	−0.052(0.3)	0.032(0.5)
MEdarkolivegreen2	0.085(0.09)	−0.096(0.06)	0.054(0.3)	−0.058(0.3)	0.16(0.001)
MElightpink2	−0.04(0.4)	0.011(0.8)	0.053(0.3)	−0.044(0.4)	0.058(0.3)
MEdarkolivegreen	−0.29(1e−08)	0.066(0.2)	0.15(0.002)	0.17(0.001)	−0.28(4e−08)
MElightsteelblue1	−0.063(0.2)	0.0064(0.9)	0.11(0.03)	0.045(0.4)	−0.055(0.3)
MEmagenta2	0.087(0.09)	−0.09(0.08)	−0.04(0.4)	−0.063(0.2)	0.076(0.1)
MEgreen4	0.056(0.3)	−0.079(0.1)	0.047(0.4)	0.018(0.7)	−0.039(0.4)
MEviolet	0.077(0.1)	−0.095(0.06)	−0.065(0.2)	0.034(0.5)	0.028(0.6)
MEfirebrick2	0.067(0.2)	−0.079(0.1)	0.052(0.3)	−0.052(0.3)	0.011(0.8)
MEchocolate4	0.056(0.3)	−0.091(0.08)	−0.061(0.2)	−0.022(0.7)	0.08(0.1)
MEskyblue4	0.067(0.2)	−0.075(0.1)	0.05(0.3)	−0.056(0.3)	0.059(0.2)
MElightpink1	0.075(0.1)	0.029(0.6)	0.038(0.5)	−0.062(0.2)	0.053(0.3)
MEplum2	−0.03(0.6)	−0.091(0.07)	−0.053(0.3)	−0.0025(1)	0.044(0.4)
MEmediumpurple2	0.073(0.2)	−0.097(0.06)	−0.072(0.2)	−0.037(0.5)	0.069(0.2)
MEplum3	−0.032(0.5)	−0.11(0.04)	−0.059(0.3)	−0.077(0.1)	0.015(0.8)
MEtan3	0.075(0.1)	−0.091(0.07)	0.055(0.3)	−0.025(0.6)	0.16(0.001)
MEbrown1	0.072(0.2)	−0.094(0.07)	−0.056(0.3)	−0.15(0.004)	0.068(0.2)
MEcoral	0.065(0.2)	0.017(0.7)	0.04(0.4)	−0.08(0.1)	0.023(0.7)
MEfirebrick4	0.077(0.1)	−0.097(0.06)	0.043(0.4)	−0.059(0.2)	0.036(0.5)
MElightblue3	0.081(0.1)	−0.095(0.06)	0.044(0.4)	−0.13(0.008)	0.022(0.7)
MElavenderblush2	0.055(0.3)	0.015(0.8)	−0.066(0.2)	−0.077(0.1)	0.04(0.4)
MElightskyblue4	0.076(0.1)	0.021(0.7)	0.067(0.2)	−0.028(0.6)	0.046(0.4)
MEmagenta	0.13(0.01)	−0.11(0.02)	−0.097(0.06)	−0.13(0.01)	0.33(3e−11)
MEred	0.17(9e−04)	−0.071(0.2)	−0.0015(1)	−0.079(0.1)	0.33(6e−11)
MEgreen	0.29(4e−09)	−0.048(0.3)	0.13(0.01)	−0.19(1e−04)	0.31(9e−10)
MEturquoise	0.25(6e−07)	0.044(0.4)	0.16(0.001)	−0.15(0.003)	0.17(9e−04)
MEpink	0.13(0.01)	0.12(0.02)	0.14(0.007)	0.025(0.6)	0.12(0.02)
MEblack	0.19(2e−04)	−0.011(0.8)	0.15(0.003)	−0.081(0.1)	0.16(0.002)
MElightcyan	0.11(0.03)	0.0052(0.9)	0.024(0.6)	0.077(0.1)	0.21(4e−05)

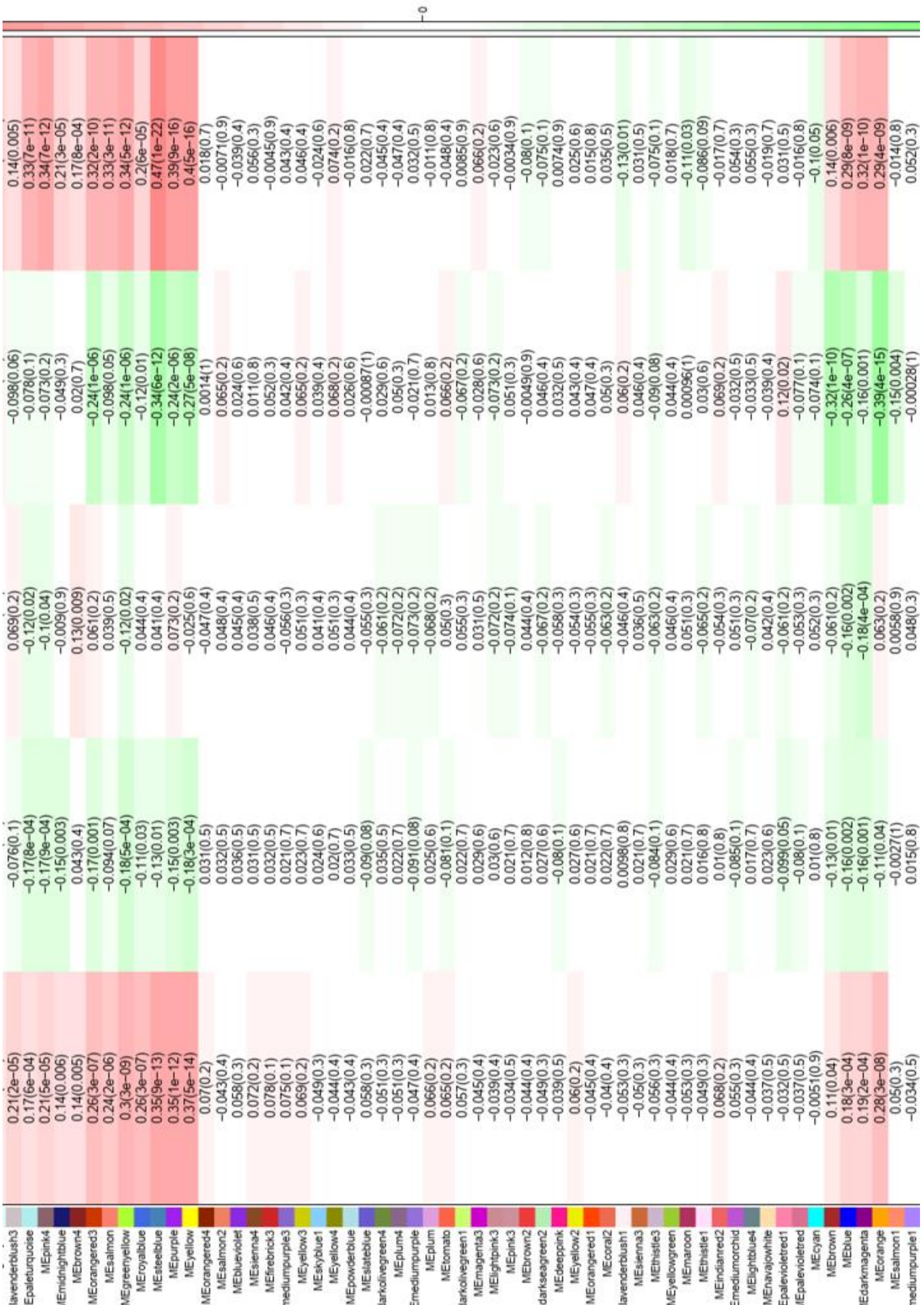

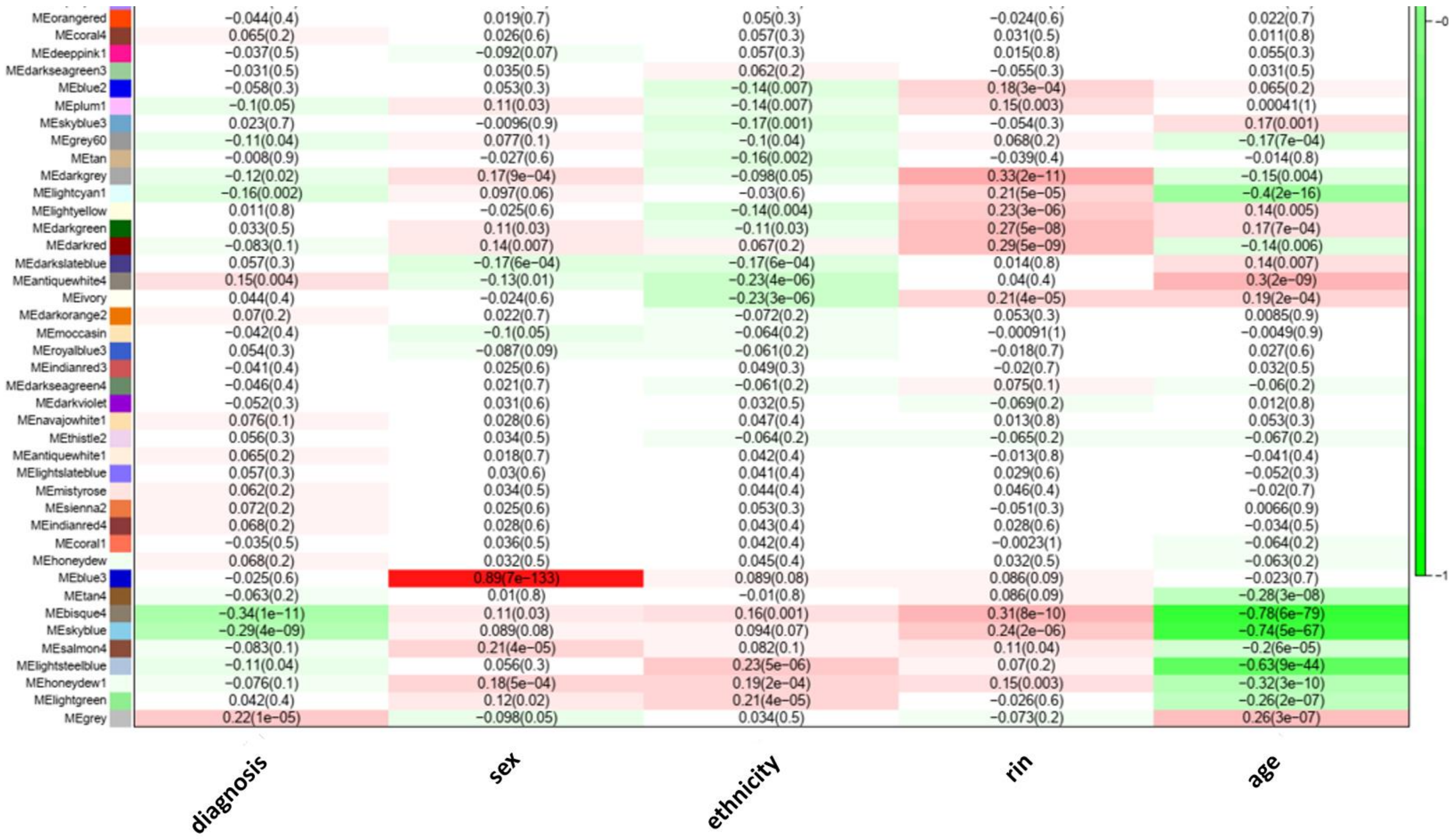
MEorangered −0.044(0.4) 0.019(0.7) 0.05(0.3) −0.024(0.6) 0.022(0.7)
MEcoral4 0.065(0.2) 0.026(0.6) 0.057(0.3) 0.031(0.5) 0.011(0.8)
MEdeeppink1 −0.037(0.5) −0.092(0.07) 0.057(0.3) 0.015(0.8) 0.055(0.3)
MEdarkseagreen3 −0.031(0.5) 0.035(0.5) 0.062(0.2) −0.055(0.3) 0.031(0.5)
MEblue2 −0.058(0.3) 0.053(0.3) −0.14(0.007) 0.18(3e−04) 0.065(0.2)
MEplum1 −0.1(0.05) 0.11(0.03) −0.14(0.007) 0.15(0.003) 0.00041(1)
MEskyblue3 0.023(0.7) −0.0096(0.9) −0.17(0.001) −0.054(0.3) 0.17(0.001)
MEgrey60 −0.11(0.04) 0.077(0.1) −0.1(0.04) 0.068(0.2) −0.17(7e−04)
MEtan −0.008(0.9) −0.027(0.6) −0.16(0.002) −0.039(0.4) −0.014(0.8)
MEdarkgrey −0.12(0.02) 0.17(9e−04) −0.098(0.05) 0.33(2e−11) −0.15(0.004)
MElightcyan1 −0.16(0.002) 0.097(0.06) −0.03(0.6) 0.21(5e−05) −0.4(2e−16)
MElightyellow 0.011(0.8) −0.025(0.6) −0.14(0.004) 0.23(3e−06) 0.14(0.005)
MEdarkgreen 0.033(0.5) 0.11(0.03) −0.11(0.03) 0.27(5e−08) 0.17(7e−04)
MEdarkred −0.083(0.1) 0.14(0.007) 0.067(0.2) 0.29(5e−09) −0.14(0.006)
MEdarkslateblue 0.057(0.3) −0.17(6e−04) −0.17(6e−04) 0.014(0.8) 0.14(0.007)
MEantiquewhite4 0.15(0.004) −0.13(0.01) −0.23(4e−06) 0.04(0.4) 0.3(2e−09)
MEivory 0.044(0.4) −0.024(0.6) −0.23(3e−06) 0.21(4e−05) 0.19(2e−04)
MEdarkorange2 0.07(0.2) 0.022(0.7) −0.072(0.2) 0.053(0.3) 0.0085(0.9)
MEmoccasin −0.042(0.4) −0.1(0.05) −0.064(0.2) −0.00091(1) −0.0049(0.9)
MEroyalblue3 0.054(0.3) −0.087(0.09) −0.061(0.2) −0.018(0.7) 0.027(0.6)
MEindianred3 −0.041(0.4) 0.025(0.6) 0.049(0.3) −0.02(0.7) 0.032(0.5)
MEdarkseagreen4 −0.046(0.4) 0.021(0.7) −0.061(0.2) 0.075(0.1) −0.06(0.2)
MEdarkviolet −0.052(0.3) 0.031(0.6) 0.032(0.5) −0.069(0.2) 0.012(0.8)
MEnavajowhite1 0.076(0.1) 0.028(0.6) 0.047(0.4) 0.013(0.8) 0.053(0.3)
MEthistle2 0.056(0.3) 0.034(0.5) −0.064(0.2) −0.065(0.2) −0.067(0.2)
MEantiquewhite1 0.065(0.2) 0.018(0.7) 0.042(0.4) −0.013(0.8) −0.041(0.4)
MElightslateblue 0.057(0.3) 0.03(0.6) 0.041(0.4) 0.029(0.6) −0.052(0.3)
MEmistyrose 0.062(0.2) 0.034(0.5) 0.044(0.4) 0.046(0.4) −0.02(0.7)
MEsienna2 0.072(0.2) 0.025(0.6) 0.053(0.3) −0.051(0.3) 0.0066(0.9)
MEindianred4 0.068(0.2) 0.028(0.6) 0.043(0.4) 0.028(0.6) −0.034(0.5)
MEcoral1 −0.035(0.5) 0.036(0.5) 0.042(0.4) −0.0023(1) −0.064(0.2)
MEhoneydew 0.068(0.2) 0.032(0.5) 0.045(0.4) 0.032(0.5) −0.063(0.2)
MEblue3 −0.025(0.6) 0.89(7e−133) 0.089(0.08) 0.086(0.09) −0.023(0.7)
MEtan4 −0.063(0.2) 0.01(0.8) −0.01(0.8) 0.086(0.09) −0.28(3e−08)
MEbisque4 −0.34(1e−11) 0.11(0.03) 0.16(0.001) 0.31(8e−10) −0.78(6e−79)
MEskyblue −0.29(4e−09) 0.089(0.08) 0.094(0.07) 0.24(2e−06) −0.74(5e−67)
MEsalmon4 −0.083(0.1) 0.21(4e−05) 0.082(0.1) 0.11(0.04) −0.2(6e−05)
MElightsteelblue −0.11(0.04) 0.056(0.3) 0.23(5e−06) 0.07(0.2) −0.63(9e−44)
MEhoneydew1 −0.076(0.1) 0.18(5e−04) 0.19(2e−04) 0.15(0.003) −0.32(3e−10)
MElightgreen 0.042(0.4) 0.12(0.02) 0.21(4e−05) −0.026(0.6) −0.26(2e−07)
MEgrey 0.22(1e−05) −0.098(0.05) 0.034(0.5) −0.073(0.2) 0.26(3e−07)
diagnosis
sex
ethnicity
rin
age
0
−1

Table 2. Below every module number we show the number of genes they contain. The module 0 contains 15314 genes which belong to the grey module that contains the total genes that couldn't be assigned to any other module. The grey module does not participate to any further analysis.

modules	0	1	2	3	4	5	6	7	8	9	10	11	12
Number of genes	15314	1863	1793	1709	1267	1181	960	877	797	661	659	607	603
	13	14	15	16	17	18	19	20	21	22	23	24	25
	487	487	460	442	434	391	385	373	325	298	281	259	258
	26	27	28	29	30	31	32	33	34	35	36	37	38
	256	234	228	201	199	194	190	185	179	175	169	168	167
	39	40	41	42	43	44	45	46	47	48	49	50	51
	167	151	145	139	138	137	136	133	130	122	120	120	116
	52	53	54	55	56	57	58	59	60	61	62	63	64
	116	116	115	114	114	112	111	111	110	107	107	106	106
	65	66	67	68	69	70	71	72	73	74	75	76	77
	105	105	103	102	102	101	101	100	99	99	98	97	96
	78	79	80	81	82	83	84	85	86	87	88	89	90
	93	93	93	92	92	90	90	89	88	88	87	86	86
	91	92	93	94	95	96	97	98	99	100	101	102	103
	86	85	84	81	80	79	79	79	78	77	77	77	76
	104	105	106	107	108	109	110	111	112	113	114	115	116
	76	76	75	75	74	74	73	72	72	71	70	69	68
	117	118	119	120	121	122	123	124	125	126	127	128	129
	68	67	67	65	65	64	63	63	63	61	61	60	58
	130	131	132	133	134	135	136	137	138	139	140	141	142
	58	57	57	57	55	55	54	54	53	53	53	53	52
	143												
	50												

A

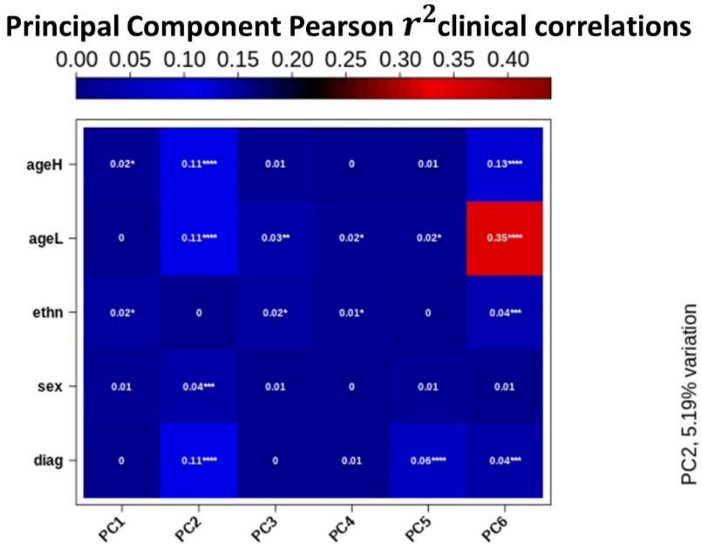

	PC#	ID	Description	GeneRatio
1	1	R-HSA-417957	P2Y receptors	2/4
2	1	R-HSA-418038	Nucleotide-like (purinergic) receptors	2/4

	BgRatio	pvalue	p.adjust	qvalue	geneID
1	12/10867	0.000006699042	0.00007304457	0.00001922225	P2RY13/P2RY12
2	16/10867	0.000012174095	0.00007304457	0.00001922225	P2RY13/P2RY12

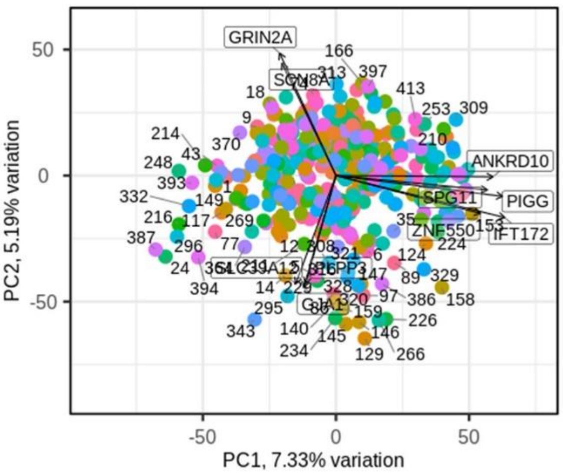

- **Number of significant surrogate variables: 1**
- **Removing the lower 0% of variables based on variance**
- **scale = False**

B

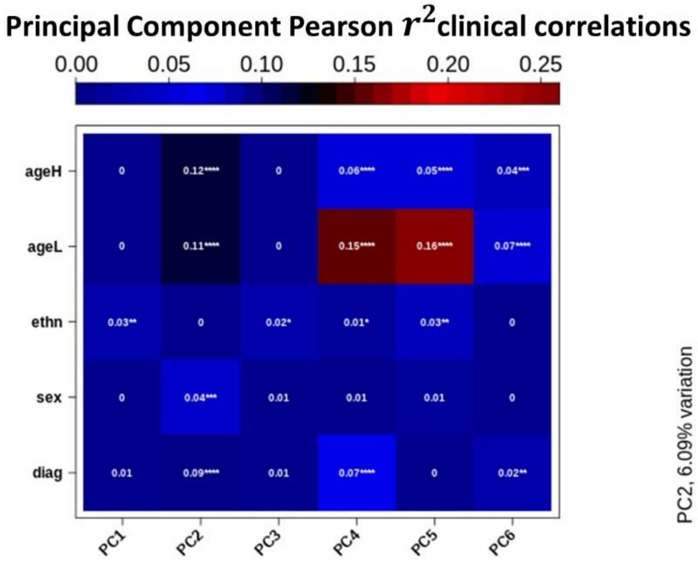

	PC#	ID
1	1	R-HSA-9614399
2	1	R-HSA-75035
3	1	R-HSA-111447
4	1	R-HSA-392517

	Description
1	Regulation of localization of FOXO transcription factors
2	Chk1/Chk2(Cds1) mediated inactivation of Cyclin B:Cdk1 complex
3	Activation of BAD and translocation to mitochondria
4	Rap1 signalling

	GeneRatio	BgRatio	pvalue	p.adjust	qvalue	geneID
1	2/6	11/10867	0.00001394264	0.000592468	0.0001679059	YWHAB/YWHAZ
2	2/6	13/10867	0.00001976349	0.000592468	0.0001679059	YWHAB/YWHAZ
3	2/6	15/10867	0.00002659163	0.000592468	0.0001679059	YWHAB/YWHAZ
4	2/6	16/10867	0.00003038297	0.000592468	0.0001679059	YWHAB/YWHAZ

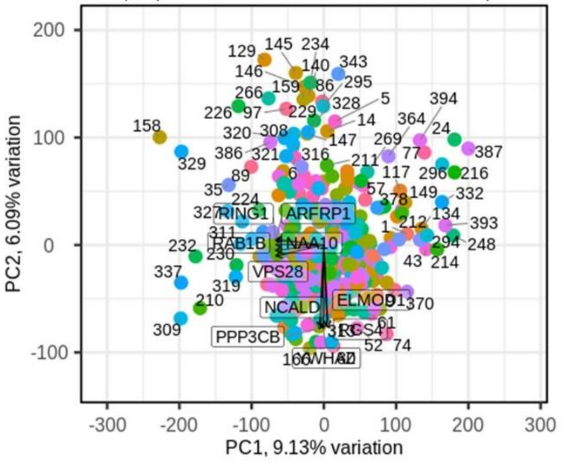

- **Number of significant surrogate variables: 1**
- **Removing the lower 10% of variables based on variance**
- **scale = True**

*Figure 1. Representing genes and samples in a 2-D space. The genes that contribute the most to each of the top 5 PCs (positive/negative) from the loading plots were used for Reactome pathway gene set enrichment analysis (see Tables in **A** and **B**).*
