# Supplementary material for: Exploration of schizophrenia-associated gene modules using graph theory, co-expression networks, and dimensionality reduction
Source: PLoS One. 2026 Apr 15;21(4):e0346663. doi: 10.1371/journal.pone.0346663 (PMC13082716; doi:10.1371/journal.pone.0346663)
Supplement: S2 File — (PDF) [file pone.0346663.s010.pdf]

Supplementary Figures

A

```
mod0 = model.matrix(~as.factor(ethn) + as.factor(sex) +
as.factor(age) + as.factor(region), data=datTraits)
(NULL model)

mod = model.matrix(~as.factor(diag) + as.factor(ethn) +
as.factor(sex) + as.factor(age) + as.factor(region),
data=datTraits)
(FULL model)
```

```
mod0 = model.matrix(~as.factor(ethn) + as.factor(sex) +
as.factor(age), data=datTraits)
(NULL model)

mod = model.matrix(~as.factor(diag) + as.factor(ethn) +
as.factor(sex) + as.factor(age), data=datTraits)
(FULL model)
```

B

```
Call:
lm(formula = Y3 ~ datTraits$diag)
Residuals:
    Min       1Q   Median       3Q      Max
-0.11038 -0.03425 -0.00597  0.03375  0.15983
Coefficients:
                 Estimate   Std. Error   t value Pr(>|t|)
(Intercept)     -0.009628   0.003157   -3.050  0.00245 **
datTraits$diag1  0.026792   0.005266    5.088  5.7e-07 ***
---
Signif. codes:  0 '***' 0.001 '**' 0.01 '*' 0.05 '.' 0.1 ' ' 1
Residual standard error: 0.04951 on 382 degrees of freedom
Multiple R-squared:  0.06346,   Adjusted R-squared:  0.06101
F-statistic: 25.88 on 1 and 382 DF, p-value: 5.695e-07
```

```
Call:
lm(formula = svobj$sv ~ datTraits$diag)
Residuals:
    Min       1Q   Median       3Q      Max
-0.09461 -0.06206  0.02571  0.04066  0.06141
Coefficients:
                Estimate Std. Error t value Pr(>|t|)
(Intercept)      0.004097   0.003243   1.263   0.2073
datTraits$diag1 -0.011401   0.005410  -2.107   0.0357 *
---
Signif. codes:  0 '***' 0.001 '**' 0.01 '*' 0.05 '.' 0.1 ' ' 1
Residual standard error: 0.05087 on 382 degrees of freedom
Multiple R-squared:  0.01149,   Adjusted R-squared:  0.008904
F-statistic: 4.441 on 1 and 382 DF, p-value: 0.03574
```

Figure S1: (Supplementary Fig. S1) Surrogate variable analysis (SVA) selection and design matrices used for SV estimation. Full code and model matrices are available in the Supplementary R scripts.

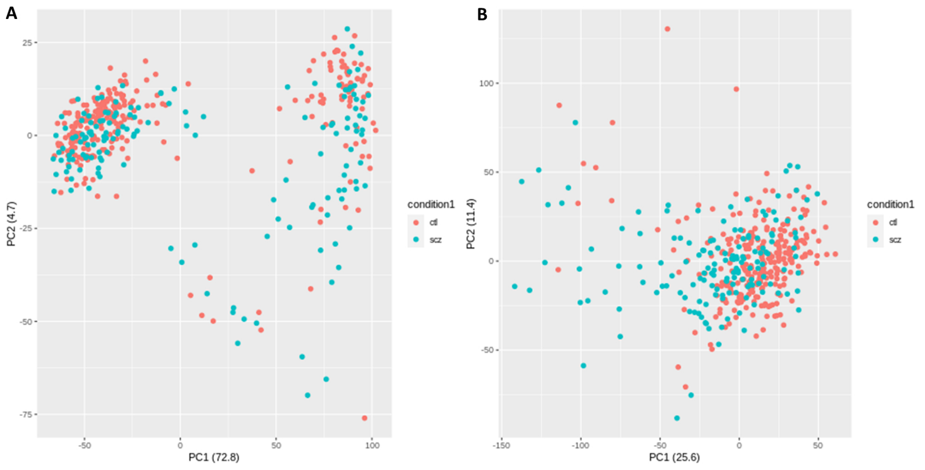

Figure S2: (Supplementary Fig. S2) PCA of differentially expressed genes before (left) and after (right) covariate correction.

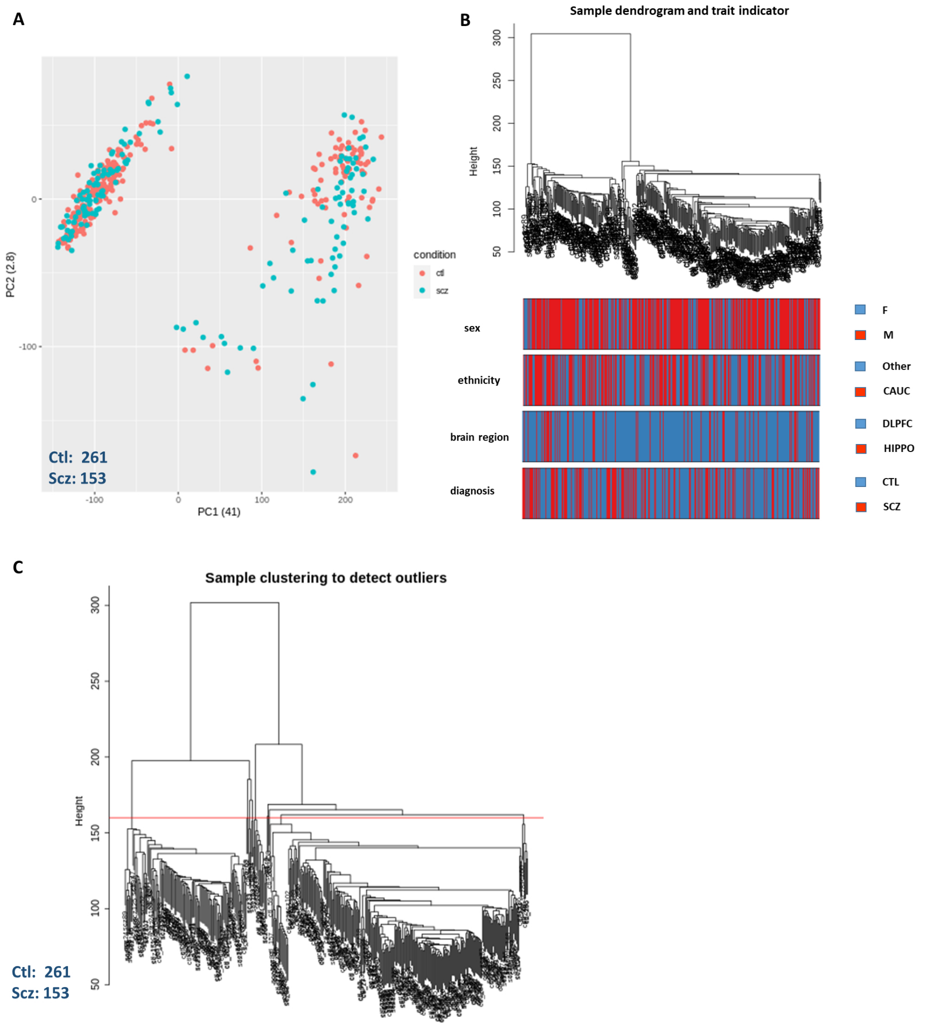

Figure S3: (Supplementary Fig. S3) Sample-level PCA and hierarchical clustering dendrogram used for outlier detection (red cut line indicates retained cluster).

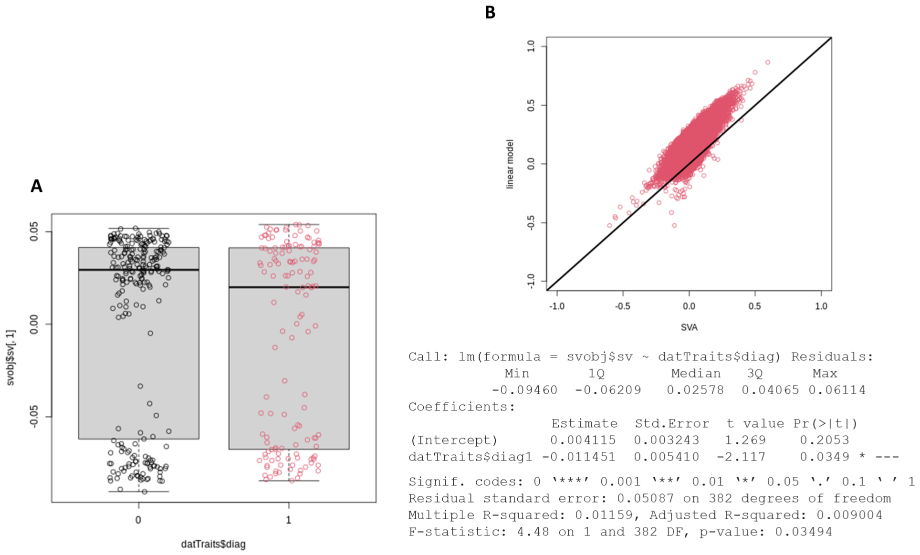


```
Call: lm(formula = svobj$sv ~ datTraits$diag) Residuals:
           Min       1Q        Median    3Q       Max
         -0.09460  -0.06209   0.02578  0.04065 0.06114
Coefficients:
                    Estimate  Std.Error  t value Pr(>|t|)
(Intercept)        0.004115  0.003243   1.269    0.2053
datTraits$diag1 -0.011451  0.005410  -2.117    0.0349 * ---

Signif. codes: 0 '***' 0.001 '**' 0.01 '*' 0.05 '.' 0.1 ' ' 1
Residual standard error: 0.05087 on 382 degrees of freedom
Multiple R-squared: 0.01159, Adjusted R-squared: 0.009004
F-statistic: 4.48 on 1 and 382 DF, p-value: 0.03494
```

Figure S4: (Supplementary Fig. S4) SVA diagnostics: boxplots of retained SV values by diagnosis and coefficient estimates testing SV association with diagnosis.

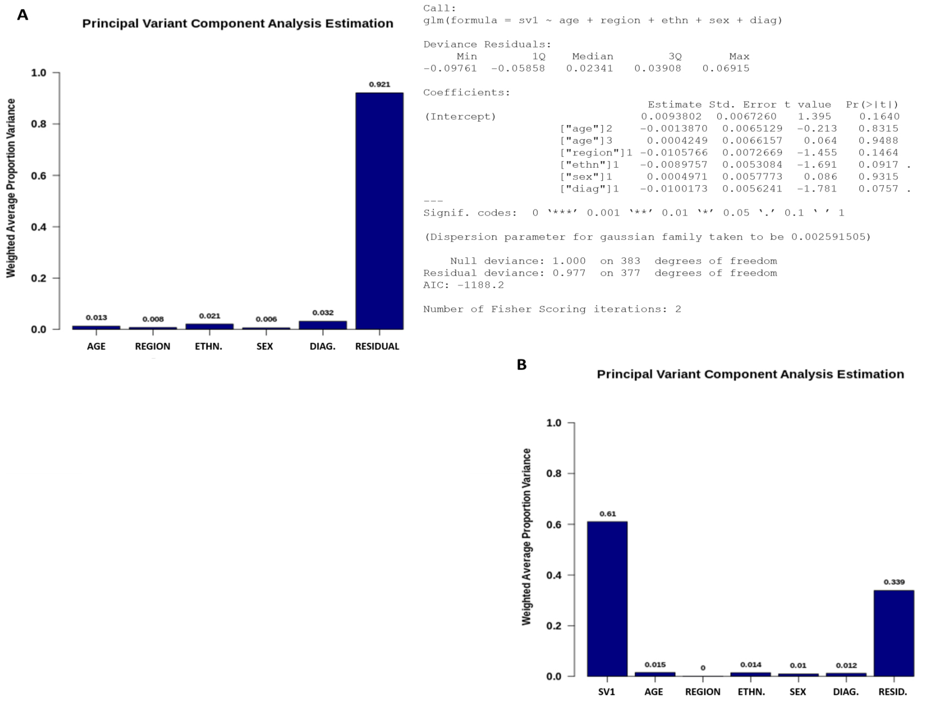


```
Call:
glm(formula = sv1 ~ age + region + ethn + sex + diag)

Deviance Residuals:
     Min        1Q     Median        3Q       Max
-0.09761  -0.05858   0.02341   0.03908   0.06915

Coefficients:
                                 Estimate Std. Error t value  Pr(>|t|)
(Intercept)                     0.0093802  0.0067260   1.395    0.1640
                   ["age"]2    -0.0013870  0.0065129  -0.213    0.8315
                   ["age"]3     0.0004249  0.0066157   0.064    0.9488
                   ["region"]1 -0.0105766  0.0072669  -1.455    0.1464
                   ["ethn"]1   -0.0089757  0.0053084  -1.691    0.0917 .
                   ["sex"]1     0.0004971  0.0057773   0.086    0.9315
                   ["diag"]1   -0.0100173  0.0056241  -1.781    0.0757 .
---
Signif. codes:  0 '***' 0.001 '**' 0.01 '*' 0.05 '.' 0.1 ' ' 1

(Dispersion parameter for gaussian family taken to be 0.002591505)

    Null deviance: 1.000  on 383  degrees of freedom
Residual deviance: 0.977  on 377  degrees of freedom
AIC: -1188.2

Number of Fisher Scoring iterations: 2
```

Figure S5: (Supplementary Fig. S5) PVCA variance decomposition summary showing proportions explained by covariates, SVs, and residuals.

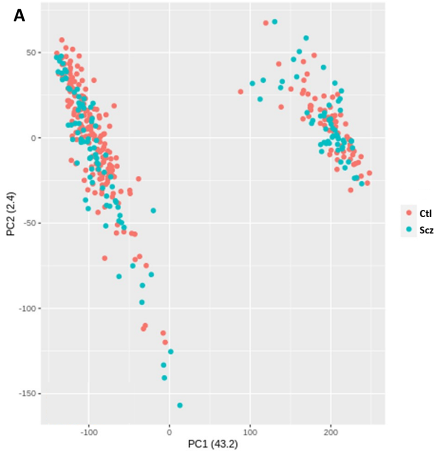

Figure S6: (Supplementary Fig. S6) Protocol heterogeneity diagnostics: PCA colored by rRNA depletion kit and post-adjustment PCA.

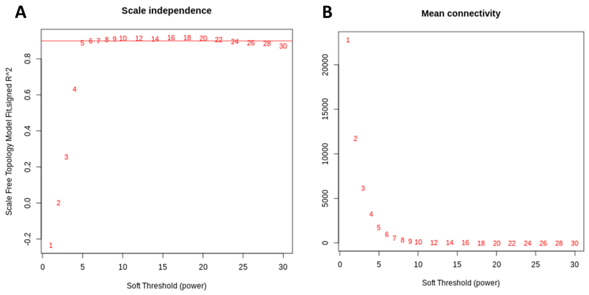

	Power	SFT.R.sq	slope	truncated.R.sq	mean.k.	median.k.	max.k.
1	1	2.34e-01	14.7000	0.925	22800.00	2.27e+04	24700.0
2	2	1.39e-05	-0.0499	0.842	11700.00	1.15e+04	14200.0
3	3	2.57e-01	-4.3600	0.722	6140.00	5.92e+03	8650.0
4	4	6.32e-01	-5.0200	0.787	3270.00	3.06e+03	5520.0
5	5	8.89e-01	-4.9700	0.931	1780.00	1.60e+03	3740.0
6	6	9.01e-01	-4.2400	0.931	991.00	8.46e+02	2660.0
7	7	9.01e-01	-3.5600	0.931	568.00	4.51e+02	1970.0
8	8	9.07e-01	-3.0500	0.938	336.00	2.44e+02	1510.0
9	9	9.13e-01	-2.6800	0.949	206.00	1.34e+02	1200.0
10	10	9.14e-01	-2.4100	0.958	131.00	7.43e+01	968.0
**11**	**12**	**9.13e-01**	**-2.0600**	**0.970**	**59.50**	**2.45e+01**	**668.0**
12	14	9.13e-01	-1.8500	0.979	31.20	8.67e+00	485.0
13	16	9.18e-01	-1.7400	0.988	18.40	3.33e+00	364.0
14	18	9.16e-01	-1.6900	0.991	11.80	1.41e+00	282.0
15	20	9.16e-01	-1.6800	0.993	8.02	6.28e-01	224.0
16	22	9.07e-01	-1.6800	0.992	5.74	2.92e-01	181.0
17	24	8.98e-01	-1.6800	0.993	4.26	1.42e-01	148.0
18	26	8.91e-01	-1.6700	0.996	3.27	7.17e-02	122.0
19	28	8.85e-01	-1.6500	0.995	2.58	3.69e-02	101.0
20	30	8.70e-01	-1.6200	0.987	2.10	1.94e-02	84.8

Figure S7: (Supplementary Fig. S7) Soft-threshold selection (pickSoftThreshold output) and permutation-based PC scree diagnostics used to select WGCNA power and PCs retained for t-SNE.
