# Supplementary material for: Exploration of schizophrenia-associated gene modules using graph theory, co-expression networks, and dimensionality reduction
Source: PLoS One. 2026 Apr 15;21(4):e0346663. doi: 10.1371/journal.pone.0346663 (PMC13082716; doi:10.1371/journal.pone.0346663)
Supplement: Attachment — Submitted filename: PLOS_ONE_responses_to_reviewers.pdf [file pone.0346663.s011.pdf]

Responses to reviewers' comments

Reviewer #1:

Weaknesses and recommendations:

1. **Improving clarity and organization:** the reviewer requests a single comprehensive diagram/flowchart linking the three pipelines; re-render figures; clearer labels and focused captions.

   Reply: Please refer to figure 1. We present a schematic of the integrated three-pipeline architecture. Also, the labels of all figures have been re-written for brevity.

2. **Statistical justification of methodological choices:** specifically, justification for using a correlation threshold of 0.8 for the igraph network; the marginal p-value (p = 0.04) for a key community should be interpreted cautiously.

   Reply: The manuscript reports using absolute Spearman correlations and applying a threshold of $|\rho| \geq 0.8$ when constructing the DEG-based graph and reports Community 2 significance as approximately $p = 0.04$.

   The correlation threshold of 0.8 requires explicit justification grounded in network topology principles. In our case, after many trials the threshold of 0.8 was selected to create a sparse, interpretable network that emphasizes strong, biologically meaningful correlations while filtering noise from weak correlations. The practical cut-off was also cross-referenced in the literature: Recent methodological work by Toubiana et al. (2021) on correlation thresholding in biological networks demonstrates that "the selection of the correlation coefficient threshold...depends on the network topology rather than on the strength of the correlation coefficient itself." Their analysis of correlation networks showed that thresholds between 0.41 and 0.84 were appropriate depending on network properties, and they identified optimal thresholds by analyzing the distribution of network edges at varying correlation levels. This threshold is conservative (high), which aligns with recommendations in the literature for dense gene co-expression matrices where thousands of genes may be highly interconnected.

   About the significance of Community 2, this concern is addressed directly in the manuscript. In the Results section we state: "the significance of Community 2, despite being the largest in the pathogenic network, was marginal ($p \approx 0.04$), raising concerns about the robustness and reproducibility of this module. Therefore, interpretations must be made with caution, and further validation is warranted to confirm its biological relevance." This marginal p-value ($p = 0.04$) falls below the conventional threshold of $p < 0.05$, but should be interpreted in the context of multiple-testing correction and the network-permutation procedures applied in our analysis.

3. **Explicitly state the new scientific contribution:** immune/synaptic/epigenetic involvement in schizophrenia is established; clarify novelty.

   Reply:

   We acknowledge that immune, synaptic, and epigenetic dysregulation in schizophrenia are established pathogenic pathways. Our contribution is neither to discover these pathways (which are well-documented) nor to identify a single novel gene. Rather, our innovation lies in three complementary dimensions. More specifically:

   (a) Orthogonal integration of three independent computational methods revealing non-redundant biological signatures supported by the literature: We acknowledge this critical

point regarding the distinction and complementarity of methods. The key innovation lies not in applying individual methods, but in the integrative analysis demonstrating how three orthogonal approaches reveal non-redundant, biologically complementary signatures of schizophrenia—each capturing distinct aspects of disease pathology that would be missed by any single method alone.

(b) Discovery and transparent handling of previously undocumented rRNA-depletion protocol heterogeneity in PsychENCODE (obtained via personal communication with data curators): Explicit identification, characterization, and correction of previously unrecognized technical confounding in PsychENCODE data—namely, heterogeneous rRNA-depletion protocols that cascade into artifactual batch effects masquerading as biological signal.

(c) Multi-dimensional ranking system combining network topology, co-expression membership, phenotypic correlation, and druggability: we evaluate each therapeutic target gene across four independent criteria, rather than relying on just one. Their importance and influence within the disease-specific gene regulatory network topology, their membership and relevance within disease-associated co-expression gene modules, their direct correlation with clinical phenotypes such as diagnosis status, and their potential druggability—meaning they possess biological features or existing pharmacological evidence making them feasible targets for therapeutic intervention. This is a systematic, rigorous approach to therapeutic target prioritization which represents good practice in precision medicine.

Please refer to the main manuscript, Section 'Novelty and contribution'.

Reviewer #2: Full comments (excerpt and specific points)

Reviewer comment (overview): The authors present results from three different methods to analyze gene expression data from PsychENCODE: a custom graph analysis, WGCNA, and PCA. The specifics of the exact PsychENCODE data used are not clear; clarify data acquisition. Multiple analytic choices in the custom graph method require justification, including Spearman scaling, MST-only backbone, sample categorization and origin of multiple edges. The reviewer questions whether the custom graph method provides novel insights beyond WGCNA/PCA and requests justification.

Specific points and replies:

1. **Data specifics / Data acquisition:** The reviewer requests focus on the exact dataset used (which PsychENCODE release/file), explanation of sources of batch effects, and a suggestion to add a Data acquisition subsection.

   Reply: Please refer to section Data Acquisition and Preprocessing. We have expand the description of the PsychEncode Consortium with information on the exact datasets we have processed and analyzed, along with a description of the pre-processing pipeline we have developed. For implementation details and reproducibility, see also the attached code.

2. **Why scale Spearman between [-1,1]?** Spearman is already bounded; clarify.

   Reply: Regarding Spearman correlation scaling: Spearman's rank correlation coefficients naturally range from -1 to 1, and our statement that we "scale" these values within this

range refers to normalization across datasets and samples to ensure comparability. The precise language has been improved in the revision to avoid ambiguity.

Please refer to Section 2.1.2 (Methods): The data are then log2-transformed, and we compute the correlation matrix using Spearman correlation coefficients. We use the correlation matrix to generate the adjacency matrix.

The choice of Spearman correlation rather than Pearson is justified because our gene expression data exhibit non-normal distributions, as demonstrated in Figure 3. Recent methodological studies comparing correlation metrics for RNA-seq data show that Spearman correlation has superior performance for non-normal, count-based sequencing data compared to Pearson correlation. Specifically, Siska et al. (2017) demonstrated that "Spearman correlation is rank-based and a non-parametric alternative that can handle non-normal data and capture monotonic and linear relationships," and their comprehensive benchmarking found that Spearman had greater area under the curve in both ROC and sensitivity curves compared to Pearson, BWMC, and SparCC methods. Our Q-Q plots (Figure 3) explicitly show deviation from normality, supporting this choice.

3. **Why only the MST? Might we discard information?**

   Reply: The use of a minimum spanning tree is not merely a computational convenience but reflects a biologically motivated network reduction strategy. The MST preserves the minimal set of strongest correlations necessary to maintain network connectivity while eliminating redundant edges. This approach has biological precedent: Rahmatallah et al. (2014) demonstrated that "the union of the first and second MST, constructed from using correlation distances, gives the minimal set of essential links among genes, which we interpret as a network of functional interactions". The authors further showed that "genes in the center of MST tend to have large weights, and hub genes—genes with the largest weight in the pathways—correspond in real data frequently to pathway regulators." By retaining only essential connections, the MST approach reduces noise while preserving the topological structure that identifies functionally relevant hub genes. This is particularly important in high-dimensional gene expression data where spurious correlations can obscure biologically meaningful relationships. The alternative—retaining all edges above a correlation threshold—would create a dense, highly interconnected network that violates scale-free assumptions and obscures identification of true regulatory hubs.

   Please see addition in section "Constructing gene co-expression networks"

4. **How samples/data were categorized and origin of multiple edges between nodes?**

   Reply: The PDF states that two separate trees were generated (one for 261 controls and one for 153 schizophrenia samples) and that graph simplification eliminated multiple edges and small disjoint components prior to analysis. This indicates that correlations were computed within each diagnostic group and that any multiple edges encountered during intermediate graph-building were collapsed before final community detection. The submitted manuscript therefore documents these points in Methods 2.1.2.

   We generated two separate minimum spanning trees—not one combined network—to facilitate disease-condition comparison: one tree constructed from the 261 control samples and a separate tree constructed from the 153 schizophrenia patients. This separation allows for comparative analysis of network topology between healthy and diseased states, which is essential for identifying disease-specific hub genes and modules. Each tree is constructed independently

from gene expression data within each diagnostic group, and edges represent pairwise Spearman correlations between genes within that group. There are not "multiple edges between two nodes" within a single MST; rather, each MST is acyclic and spans all genes with minimal total edge weight. The differences observed between the control and schizophrenia trees reflect genuine differences in co-expression patterns between these biological conditions, not redundancy. The distinction between these two networks is critical for the biological findings: as noted in the Results, "the pathogenic network exhibited a more concentrated distribution, with a few nodes assuming highly prominent roles," whereas the control network showed more even centrality distribution. This comparative network topology analysis enabled identification of genes like AKT3, which are central in the disease but not in the control network, representing potential therapeutic targets with disease specificity.

5. **Innovation / Why compare PCA separately from WGCNA; could WGCNA capture PCA components?**

   Reply: Regarding the relationship between WGCNA and PCA: WGCNA uses PCA for dimensionality reduction but differs fundamentally in its analytical objective. WGCNA identifies modules of co-expressed genes (clusters of genes sharing correlated expression) and relates these modules to clinical traits through eigengene analysis. In contrast, our PCA analysis identifies individual genes whose expression contributes most significantly to variance along principal components associated with diagnosis. DiLeo et al. (2011) explicitly state: "While PCA and BL-SOM are appropriate and commonly used methods, WGCNA holds several advantages in the analysis of highly multivariate, complex data...PCA separated the two major genetic backgrounds..., but provided little further information. Both BL-SOM and WGCNA clustered metabolites by expression, but WGCNA additionally defined 'modules' of co-expressed metabolites explicitly and provided additional network statistics that described the systems properties". Our PCA-based enrichment analysis complements WGCNA by identifying diagnostic variance-driven genes without imposing module structure assumptions, capturing genes that may not cluster within co-expression modules but whose individual expression patterns distinguish cases from controls.

   Why the igraph method provides non-redundant insights: The igraph approach prioritizes network topology (centrality measures) and is specifically designed to identify hub genes whose connectivity patterns distinguish disease from control networks. Unlike WGCNA, which clusters genes into modules, igraph examines topological properties across the entire network. Critically, we observed that AKT3, identified as central in the disease network's largest community (Community 2), is enriched in specific pathways (mRNA export) that were not prominently identified by WGCNA's module structure. The igraph finding that AKT3 is "central but non-core" (highly connected but not formally within its own detected community) suggests regulatory hub function distinct from co-expression clustering. This distinction has biological implications: recent literature identifies AKT3 as a schizophrenia-associated gene and therapeutic target through converging GWAS, network analysis, and in vivo evidence. The igraph method successfully prioritized this gene through topological centrality, whereas module-based methods might miss such regulatory hubs operating at network peripheries.

   Method-specific insights summarized:

   - igraph: Identifies disease-specific hub genes through topology; highlights transporters, synaptic transmission, and ion channels.
   - WGCNA: Identifies co-expression modules enriched in immune, epigenetic, and mRNA

metabolism pathways; provides module-trait associations and eigengene-based summarization.

- PCA: Isolates variance-driving genes; emphasizes neurotransmitter release and cytokine signaling without imposing module assumptions.

  Each method captures a distinct biological layer of schizophrenia pathology. This multi-method integration represents the study's innovation: demonstrating convergent but non-redundant evidence for synaptic, immune, and epigenetic dysregulation through independent analytical frameworks. This convergence across independent analytical frameworks strengthens biological confidence in the findings. The findings of immune, synaptic, and epigenetic dysregulation in schizophrenia are supported by extensive postmortem omics literature. Recent large-scale studies—including those analyzing PsychENCODE data—consistently report enrichment of these pathways. Darby et al. (2016) performed RNA-seq in multiple brain regions from hundreds of schizophrenia and control brains and found "strong enrichment of RNA-related processes: messenger RNA processing/export and protein synthesis pathways...among the most consistently altered across regions and disorders". Similarly, Choudhury et al. (2023) analyzing PsychENCODE data identified "global reduction in A-to-I RNA editing in SCZ, especially at 3-UTRs of mitochondria-related genes," indicating "widespread disruption of RNA metabolism in SCZ cortex". Our multi-method approach provides orthogonal validation of these established findings while demonstrating their robustness across distinct computational frameworks.

References and resources cited in the response

[1] https://pubmed.ncbi.nlm.nih.gov/33691629/ — Toubiana D, Maruenda H. Guidelines for correlation coefficient threshold settings in metabolite correlation networks exemplified on a potato association panel. BMC Bioinformatics. 2021;22(1):116. doi:10.1186/s12859-021-03994-z.

[2] https://pmc.ncbi.nlm.nih.gov/articles/PMC5244536/ — Siska, C. and Kechris, K. (2017). Differential correlation for sequencing data. BMC Research Notes, 10, 54. doi:10.1186/s13104-016-2331-9.

[3] https://academic.oup.com/bioinformatics/article/30/3/360/228442 — Rahmatallah, Y., Emmert-Streib, F., and Glazko, G. (2014). Gene Sets Net Correlations Analysis (GSNCA): a multivariate differential coexpression test for gene sets. Bioinformatics, 30(3), 360–368.

[4] https://pmc.ncbi.nlm.nih.gov/articles/PMC3198806/ — DiLeo, M. V., Strahan, G. D., den Bakker, M., and Hoekenga, O. A. (2011). Weighted correlation network analysis (WGCNA) applied to the tomato fruit metabolome.* PLoS ONE, 6(10), e26683.

[5] https://pmc.ncbi.nlm.nih.gov/articles/PMC10081846/ — Choudhury, M., Fu, T., Amoah, K., Jun, H.-I., Chan, T. W., Park, S., Walker, D. W., Bahn, J. H., and Xiao, X. (2023). Widespread RNA hypoediting in schizophrenia and its relevance to mitochondrial function. Science Advances, 9(14), eade9997.

[6] https://www.ncbi.nlm.nih.gov/pmc/articles/PMC5048210/ — Darby, M. M., Yolken, R. H., and Sabunciyan, S. (2016). Consistently altered expression of gene sets in post-

mortem brains of individuals with major psychiatric disorders. Transl Psychiatry, 6(9), e890. doi:10.1038/tp.2016.173.

[7] PsychENCODE Consortium. PsychENCODE Resource Portal.Available at: `http://resource.psychencode.org`.
