# Supplementary material for: Exploration of schizophrenia-associated gene modules using graph theory, co-expression networks, and dimensionality reduction
Source: PLoS One. 2026 Apr 15;21(4):e0346663. doi: 10.1371/journal.pone.0346663 (PMC13082716; doi:10.1371/journal.pone.0346663)
Supplement: Attachment — Submitted filename: Response to Reviewers.pdf [file pone.0346663.s012.pdf]

Revision Comments to the Reviewers

Reviewer #3:

- **This manuscript seeks to leverage existing PsychENCODE data to characterize transcriptomic signatures in schizophrenia postmortem brain samples. The authors state that they employ three primary analytical frameworks: igraph, WGCNA, and dimensionality reduction. However, upon reading, igraph and dimensionality reduction approaches appear to be used largely for quality control, whereas WGCNA serves as the primary analytical method to derive biological conclusions. Despite this, the WGCNA results are discussed largely at a descriptive level and do not provide clear new biological insight into schizophrenia biology.**

We must respectfully clarify the distinct, parallel roles of our three analytical pipelines. igraph and dimensionality reduction (PCA) were not used merely for quality control; rather, they were deployed as independent, primary analytical frameworks that generated biological conclusions entirely orthogonal to the WGCNA results. As illustrated in our integrated architecture (Figure 1), all three pipelines acted as primary engines that converged on functional enrichment analysis.

While PCA was indeed utilized in our shared preprocessing layer for outlier detection , it subsequently served as a primary variance-driven analytical lens. We systematically extracted the top 1% and 10% of variance-driving genes from the principal components most significantly correlated with clinical diagnosis (e.g., PC1). This direct dimensionality-reduction approach yielded independent biological insights, such as the identification of SNAP25 and its specific involvement in neurotransmitter release cycles—findings derived strictly from PCA gene loadings, not WGCNA.

Similarly, the igraph pipeline was not a QC step. It was utilized to construct topological gene co-expression networks and compute formal centrality measures (degree, eigenvector, closeness, betweenness). As noted in our previous responses regarding our dynamic thresholding and MST construction, this pipeline was explicitly calibrated to extract the structural and repressive backbones of the disease state. This allowed us to identify topological bottlenecks and hub genes with high centrality (such as AKT3 and its link to mRNA export from the nucleus) that are functionally influential despite not being core components of WGCNA's dense correlation modules.

Regarding the biological insights derived from these methods, we explicitly acknowledge in the manuscript that immune, synaptic, and epigenetic dysregulation are well-documented pathogenic pathways in schizophrenia. Our contribution is not to claim the discovery of a single, unprecedented causal gene or to re-discover these broad pathways. Rather, our primary biological and translational insight is the orthogonal integration of these three independent computational methods to overcome the high dimensionality and batch effects inherent

in brain tissue data. Because no single method can capture the full molecular complexity of schizophrenia, we designed a multi-dimensional ranking system. We evaluate therapeutic targets based on their structural bottleneck centrality (igraph), their co-expression membership (WGCNA), their variance-driven phenotypic correlation (PCA), and their pharmacological druggability.

- **While the authors primarily rely on PsychENCODE data, none of the core PsychENCODE publications are cited. At a minimum, the following two capstone papers should be cited, as they established the foundational analyses and resources used in this study: Wang et al., Science (2018) and Gandal et al., Science (2018).**

The PsychENCODE resources and integrative analyses that made the datasets mentioned publicly available are described in Wang et al., Science (2018) and Gandal et al., Science (2018), and these foundational works are cited accordingly.

- **The analyses and results presented here appear largely redundant with prior PsychENCODE work. For example, Gandal et al. employed SVA to correct for confounding factors and used WGCNA to identify disease-associated co-expression modules and eigengenes. Key biological pathways highlighted in the current manuscript, including synaptic and immune pathways, have already been extensively implicated in schizophrenia by the earlier studies.**

The observation that SVA/WGCNA and synaptic/immune pathway findings overlap with prior PsychENCODE results is correct and expected given shared data, but we present new analytical axes that represent methodological additions to the published PEC work: The PEC studies created integrated constructs with no interpretability as to which method weighted more on the results. Our study aims to define if there is an overlap between different methods or each one focuses on different aspects of the disease.

In the PsychENCODE study by Gandal et al., SVA functions as a standardized preprocessing component of consortium-level data normalization. In our submitted manuscript, SVA is used as part of a study-specific correction pipeline applied before network construction (igraph MST, WGCNA, PCA). Here, the emphasis is on detecting and adjusting specific latent structure in the working dataset (e.g., technical heterogeneity such as rRNA-depletion differences) to stabilize network topology and downstream module interpretation.

Also, Wang et al. used WGCNA to produce gene communities used solely as a separate module in the integrated neural network; Gandal et al., applied WGCNA at both gene- and isoform-level modules to explicitly prove that isoform modules gave finer resolution and higher GWAS enrichment. On the other hand, we used WGCNA only at the gene level as one of three orthogonal analyses to nominate disease-associated modules for downstream target prioritization.

- **Given that the novel scientific contribution to schizophrenia biology is currently unclear, the authors should provide additional validation**

of their results (e.g., hub genes or WGCNA co-expression modules) by relating them to the known genetic architecture of schizophrenia. For example, they can test whether their prioritized genes or gene sets overlap with targets of schizophrenia GWAS variants or SCHEMA genes, leveraging existing resources such as McAfee et al., Cell Genomics (2023) for GWAS target genes and Singh et al., Nature (2022) for SCHEMA genes.

We have added a paragraph named "Validation against genetic and functional-genomic resources". To assess whether our prioritized hub genes and disease-associated modules overlap

established genetic risk sets, we compared our top hub genes to:

(i) the SCHEMA exome sequencing results (Singh et al., 2022),

(ii) GWAS target genes predicted and experimentally supported by McAfee et al.'s MPRA/Hi-C framework (Cell Genomics 2023), and

(iii) large transcriptome resources such as Fromer et al. (Nat Neurosci 2016) and the recent single-cell multi-cohort analysis by Ruzicka et al. (Science 2024).

But please take into account that transcriptomic hub-genes and genetic hit lists come from different types of evidence, so lack of gene-level overlap does not contradict shared biology:

1) Rare-variant exome studies (e.g. Singh et al.) identify genes carrying high-impact coding mutations,

2) MPRA/Hi-C experiments (e.g. McAfee et al.) nominate regulatory target genes often in a developmental context,

3) Bulk or single-cell expression studies (e.g. Fromer et al.; Ruzicka et al.) measure steady-state RNA in heterogeneous tissue.

The whole idea of Network Medicine is to reflect transcriptional centrality rather than being a direct readout of mutation burden or variant-to-gene mapping.

- **Finally, the overall organization of the manuscript is confusing. WGCNA analyses are spread across multiple sections, and the respective contributions of igraph, SVA, and PCA to major findings (schizophrenia biology) is unclear. A substantial portion of the results should be described in the Methods section. As currently structured, the manuscript appears underdeveloped.**

LaTeX source to (1) consolidate the WGCNA material into Methods, (2) make the role of igraph / SVA / PCA explicit up front, (3) slim the Results so it reports outcomes and not methods, and (4) point long parameter sweeps and plots to Supplementary.

Reviewer #4:

- Clarify the rationale for the specific correlation thresholds and variance cut-offs adopted across the different pipelines, and to discuss how alternative parameter choices might influence network topology and module stability.

Our rationale for the specific thresholds and cut-offs is rooted in our goal to ensure the two network pipelines remain strictly orthogonal. We did not want to use igraph to simply construct a standard co-expression network with lower resolution than WGCNA. Because the igraph analysis is explicitly designed to produce a sparse, visualizable backbone emphasizing topology and to identify topologically central hub genes or potential bottlenecks, its construction functions as a strict structural filter rather than a standard quantitative co-expression model. By applying a dynamic threshold and executing Prim's algorithm on raw signed weights, the code aggressively prunes the dense correlation matrix into a rigid, cycle-free skeleton built from regulatory extremes. Subsequently stripping the edge weights during community detection forces the algorithm to evaluate this minimum spanning tree strictly as a binary wiring diagram; this unweighted approach ensures that centrality metrics highlight true structural bridges connecting distinct biological domains, preventing these vital topological control points from being mathematically overshadowed by the dense, highly correlated cliques that the orthogonal WGCNA pipeline already captures.

Regarding the variance cut-offs, this was a data-driven preprocessing choice used to remove quasi-constant genes that do not contribute meaningful co-expression signals. Varying this cut-off primarily alters overall network size rather than the relative rank-ordering of high-variance, strongly co-expressed genes that drive the underlying network structure.

In contrast, we used soft-thresholding and module detection on dense weighted networks produced by WGCNA, where parameter choices (e.g., soft-threshold power, minimum module size) influence module granularity rather than the presence or absence of individual strong correlations. As expected, alternative parameter settings modify module boundaries and sizes but preserve the overall module structure and enrichment patterns, consistent with prior reports using similar data. Because exhaustive exploration of all parameter combinations would be dataset-specific and not generalizable, we focused on representative, literature-supported values and assessed robustness by comparing MST neighborhoods with WGCNA modules and by validating key findings through functional enrichment and external biological datasets. This complementary use of a sparse backbone (MST) and a dense weighted framework (WGCNA) ensures that reported biological interpretations are not driven by a single arbitrary parameter choice.

- Consider providing additional justification for the choice of Prim's algorithm over alternative spanning tree or network pruning strategies, possibly supported by sensitivity or robustness analyses.

Our choice of Prim's algorithm to construct the Minimum Spanning Tree (MST),

rather than alternative pruning strategies, was driven by both the computational nature of transcriptomic data and our specific biological objectives.

First, from a computational and graph-theory perspective, Prim's algorithm is mathematically optimized for dense networks. A gene co-expression matrix initially forms a highly interconnected, dense graph. As supported by the literature, Prim's algorithm is highly suitable and frequently invoked for building transcriptomic MSTs (e.g., Bai et al., 2023), whereas Kruskal's algorithm is generally preferred for networks that are already sparse, such as human brain structural connectomes (van Wijk et al., 2018; Yoshino et al., 2022).

Second, regarding alternative network pruning strategies (such as standard hard-thresholding), simple thresholding often leaves highly connected "hairball" cliques and disconnected fragments, obscuring true regulatory bottlenecks. Extracting an MST uniquely enforces a rigid, fully connected, cycle-free $n-1$ backbone. Crucially, as detailed in our previous responses, applying Prim's algorithm to our raw, signed correlation matrix allowed the algorithm to deterministically seek out mathematical minimums. This deliberately prioritized the strongest anti-correlations, thereby extracting the restrictive, inhibitory regulatory backbone of the disease state—a feature that alternative pruning strategies would miss. Regarding sensitivity and robustness, because the MST is a deterministic structural filter, altering network parameters primarily influences the inclusion of weaker, peripheral edges rather than the dominant regulatory backbone. Therefore, rather than performing traditional parameter-based sensitivity analyses on the MST alone, we established robustness orthogonally. We validated the structural findings of the igraph pipeline against the dense, weighted modules produced by our parallel WGCNA pipeline, and confirmed the biological validity of these topological hubs through rigorous functional enrichment that aligns with established schizophrenia literature.

- Elaborate on the biological interpretation and reproducibility of the key pathogenic modules, particularly those showing marginal statistical significance, and to clarify how these findings compare with prior PsychENCODE-based studies.

A few WGCNA communities in our manuscript fall just below strict multiple-testing cutoffs (Community 2: p.adjust=0.04966; Community 30: p.adjust=0.04345; Community 11: p.adjust 0.03416), but each maps to biology (AKT3/AKT-mTOR signaling; glutamatergic synaptic regulation; kinase/phospho-signaling) that is independently supported by PsychENCODE and recent isoform-level and review literature, so the borderline module p-values do not undermine the biological plausibility or concordance with prior large-scale resources.

- Expand the discussion on potential confounding by age, brain region, and technical heterogeneity, and to clarify how residual variance might affect the generalisability of the results.

We have included the relevant subsection "Limitations: confounding, residual

variance, and generalization":

A high residual variance fraction implies limited signal-to-noise for disease effect sizes and increases the risk that findings are cohort-specific. Practically, this affects different results unevenly: pathway-level enrichment is generally more robust to distributed residual noise than node-level, centrality/hub identification because enrichment aggregates signals across many genes; by contrast, single-gene centrality measures (degree, betweenness, eigenvector) are highly sensitive to small changes in the underlying correlation matrix and can be unstable when residual technical or cellular-composition effects remain. Consequently, claims about broad affected pathways (immune, synaptic, epigenetic) are more likely to generalize across independent cohorts than claims that a particular gene is the top therapeutic priority.

- Consider strengthening the translational perspective by more explicitly discussing how the proposed multi-dimensional ranking framework could inform experimental validation or therapeutic prioritisation.

We have included a supplemented subsection "Criteria for Prioritizing Therapeutic Targets in Schizophrenia".

Reviewer #5:

The background/introduction lacks citations.

Citations added.

Journal requirements:

1.Please ensure that your manuscript meets PLOS ONE's style requirements, including those for file naming.

The proper PLOS ONE template has been used for style requirements. Figures are not included in the main manuscript.

We have updated and cleaned all code produced for this study. Following PLOS ONE guidelines, the code archive has been uploaded as a Supporting Information file (S1_Code.zip and S1_Code_pdf.zip), and references to this archive have been mentioned in the Supporting Information section of the manuscript.
